# Cryo-EM structure and rRNA modification sites of a plant ribosome

**DOI:** 10.1016/j.xplc.2022.100342

**Published:** 2022-05-27

**Authors:** Patrick Cottilli, Yuzuru Itoh, Yuko Nobe, Anton S. Petrov, Purificación Lisón, Masato Taoka, Alexey Amunts

**Affiliations:** 1 Science for Life Laboratory, Department of Biochemistry and Biophysics, Stockholm University, 17165 Solna, Sweden; 2 Department of Chemistry, Graduate School of Science, Tokyo Metropolitan University, Minami-osawa 1-1, Hachioji-shi, Tokyo 192-0397, Japan; 3 School of Chemistry and Biochemistry, Georgia Institute of Technology, Atlanta, GA, USA; 4 Instituto de Biología Molecular y Celular de Plantas, Universitat Politècnica de València (UPV) – Consejo Superior de Investigaciones Científicas (CSIC), Ciudad Politécnica de la Innovación (CPI), Valencia 46022, Spain

**Keywords:** plant, tomato, ribosome, RNA, structure

## Abstract

Protein synthesis in crop plants contributes to the balance of food and fuel on our planet, which influences human metabolic activity and lifespan. Protein synthesis can be regulated with respect to changing environmental cues via the deposition of chemical modifications into rRNA. Here, we present the structure of a plant ribosome from tomato and a quantitative mass spectrometry analysis of its rRNAs. The study reveals fine features of the ribosomal proteins and 71 plant-specific rRNA modifications, and it re-annotates 30 rRNA residues in the available sequence. At the protein level, isoAsp is found in position 137 of uS11, and a zinc finger previously believed to be universal is missing from eL34, suggesting a lower effect of zinc deficiency on protein synthesis in plants. At the rRNA level, the plant ribosome differs markedly from its human counterpart with respect to the spatial distribution of modifications. Thus, it represents an additional layer of gene expression regulation, highlighting the molecular signature of a plant ribosome. The results provide a reference model of a plant ribosome for structural studies and an accurate marker for molecular ecology.

## Teaser

This study describes the structure of a plant ribosome and presents a quantitative mass spectrometry analysis of its rRNAs. It reveals fine features of ribosomal proteins, plant-specific rRNA modifications, and re-annotations of 30 rRNA residues. The results provide a reference model of a plant ribosome for structural studies and an accurate marker for molecular ecology.

## Introduction

Ribosomes are fundamental to all forms of life on earth. Their activity is regulated via chemical modifications of the four rRNA species: 25S, 18S, 5.8S, and 5S. The identities and locations of these modifications have been reported for human ribosomes, and their links to disease have been established (Gilles et al., 2020). In plants, numerous small nucleolar RNAs have been characterized, suggesting plant-specific sites of rRNA 2′-*O*-ribose methylation that may contribute to the translational control of gene expression (Barneche et al., 2001). Biochemical studies further identified plant-specific mechanisms involved in ribosome assembly, localization, stress response, and stalling, with implications for antiviral immunity (Palm et al., 2019; Cheong et al., 2021). In addition, stress-triggered ribosome heterogeneity has been reported in rice (Moin et al., 2017), suggesting the functional specialization of ribosomes and the potential importance of their future engineering in crops for food security. Therefore, understanding the structure of a plant ribosome and identifying specific post-transcriptional modifications of its rRNAs may assist in biotechnological studies that aim to develop plants with a higher nutrition dose, improved fruit development, and longer-lasting quality. In particular, the tomato *Solanum lycopersicum* serves as a model for such studies (The Tomato Genome Consortium, 2012). However, despite the central role of ribosomal function, the translation apparatus in the cytosol has not been structurally determined for the Plantae kingdom (*Viridiplantae*), and the data are limited to homology models based on low-resolution reconstructions (Armache et al., 2010), although plastid (Bieri et al., 2017; Boerema et al., 2018) and mitochondrial (Tobiasson et al., 2022; Waltz et al., 2020, 2021) ribosomes have been determined.

Here, we report the structure of a plant cytosolic ribosome and a quantitative mass spectrometry analysis of its rRNAs. The study reveals plant-specific modifications, re-annotates rRNA residues, and provides a reference model for structural studies and molecular ecology.

## Results and discussion

### Structure and features of the plant ribosome

**Figure 1:**
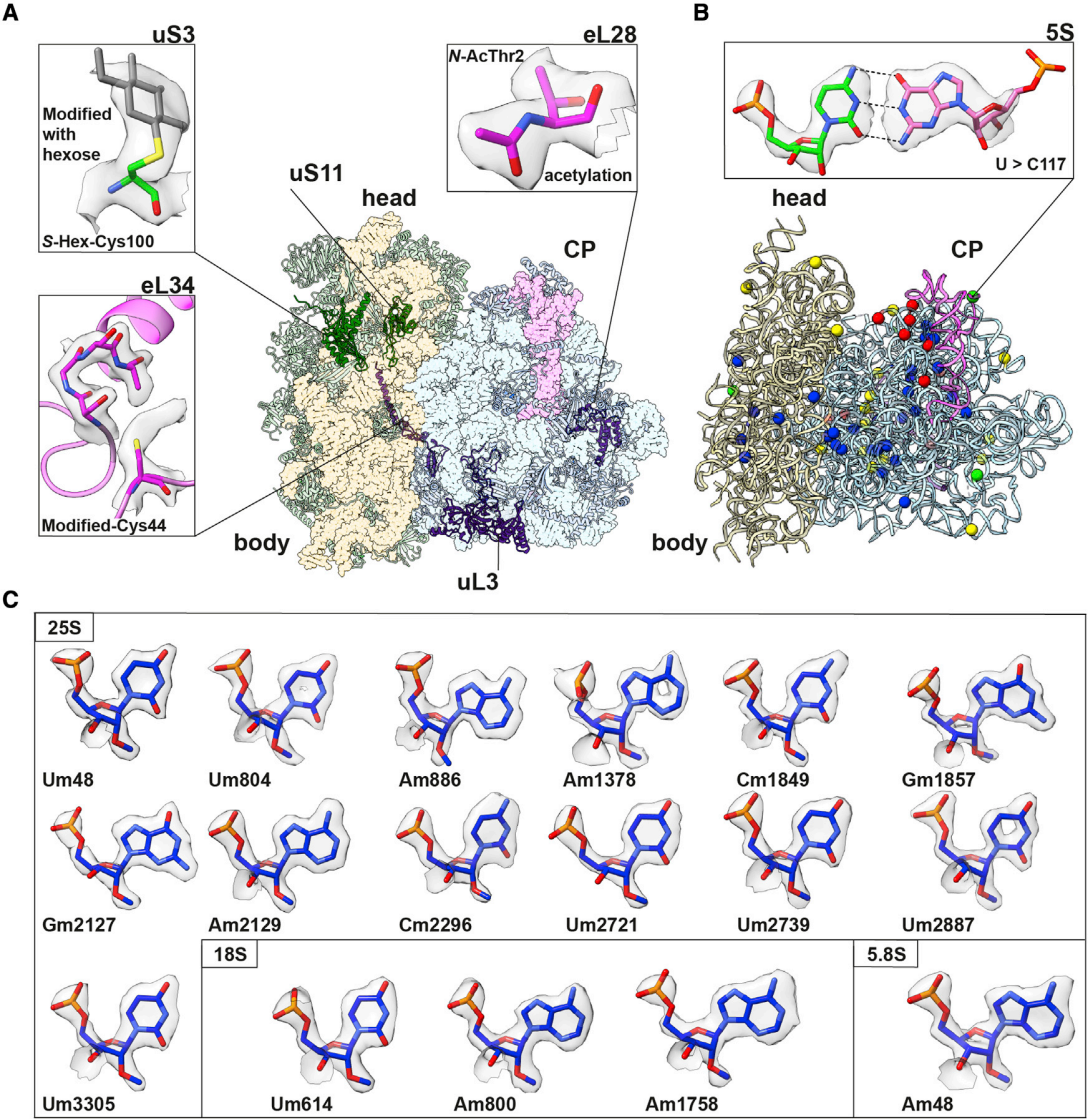
Structure and features of the plant ribosome. (A) Overall structure, featuring examples of post-translational modifications. (B) rRNA re-annotations mapped onto the 3D structure: point mutation (green), deletion (red), insertions (yellow), and plant-specific modifications (blue). One example is shown in the zoomed-in panel. (C) Representative modifications supported by the cryo-EM density. Am, Um, Gm, and Cm are 2′-*O*-methylation modifications of the respective bases.

To determine the specific features and methylation sites of a plant ribosome, we investigated *S*. *lycopersicum* ribosomes by a combination of cryo-electron microscopy (cryo-EM) and mass spectrometry–based quantitative RNA analysis. Using the structural approach, we obtained a 2.38-Å resolution cryo-EM reconstruction (Figure 1A; Supplemental Figure 1) that allowed us to build an accurate atomic model with a minimal clash score of 2.02 (Supplemental Table 1) (Amunts 2022). The model included the elucidation of plant-specific structural features and the re-annotation of 30 rRNA residues (Supplemental Tables 2 and 3), and the correct sequence has now been deposited to GenBank with accession codes OK073662–5. Using the mass spectrometry approach, we identified 71 post-transcriptional modifications (Supplemental Tables 4–7) and mapped them onto the structure (Figure 1B). With these data, we then constructed an accurate structure-based rRNA diagram by extracting the base pairs directly from the model and mapping them onto the corrected nucleotide sequence (Supplemental Figures 2 and 3). The resulting model-based diagrams map the sequence re-annotations, newly identified non-canonical base pairing, plant expansion segments, and all experimentally detected post-transcriptional modifications.

**Figure 2:**
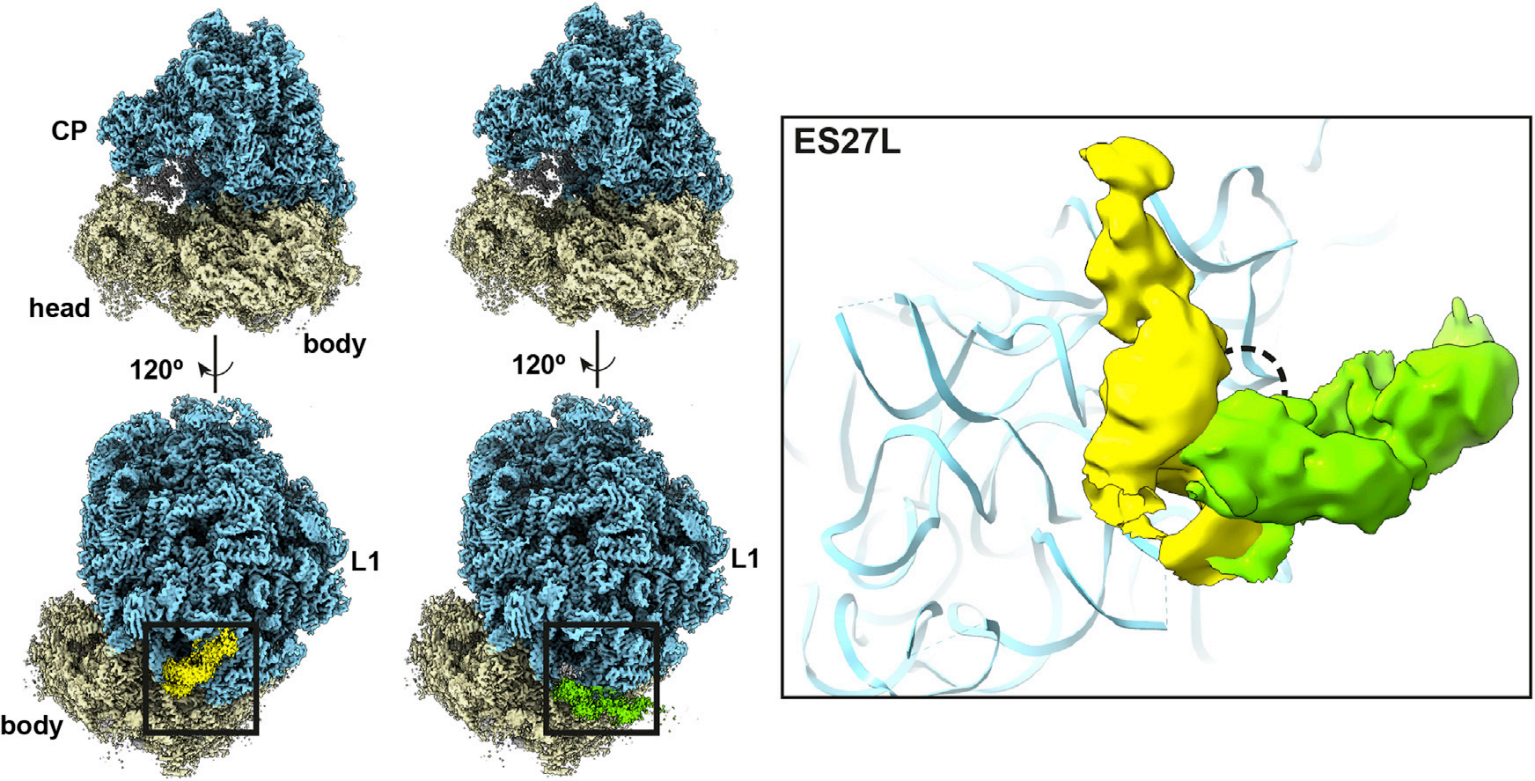
ES27L conformational change. The tomato ribosome is colored by subunit. Signal subtraction and 3D classification of the peripheral regions resulted in two defined conformations of ES27L (yellow and green). The zoomed-in panel on the right shows that the conformations in different classes are related by approximately 90°.

The overall structure of a plant ribosome is generally conserved relative to its eukaryotic counterparts, including in regions that are responsible for the binding of mRNA and tRNAs. Of the total mass of approximately 3 MDa, 10% represents rRNA expansion segments (ESs) that occur on the surface. We systematically analyzed the ESs by applying signal subtraction and three-dimensional (3D) classification to the peripheral regions. We found that, although most of the ESs are intrinsically flexible, an approximately 138-nucleotide ES27L adopts two defined conformations related by approximately 90° (Figure 2). The yeast counterpart has been shown to act as an RNA scaffold that recruits the N-terminal processing enzyme MetAP, which controls the accuracy of ribosome decoding in translation fidelity (Fujii et al., 2018). Because enzymatic activities adapt to light fluctuations in plants (Martinez-Seidel et al., 2021), the defined conformations of ES27L are likely to serve a regulatory role.

### Protein modifications

**Figure 3:**
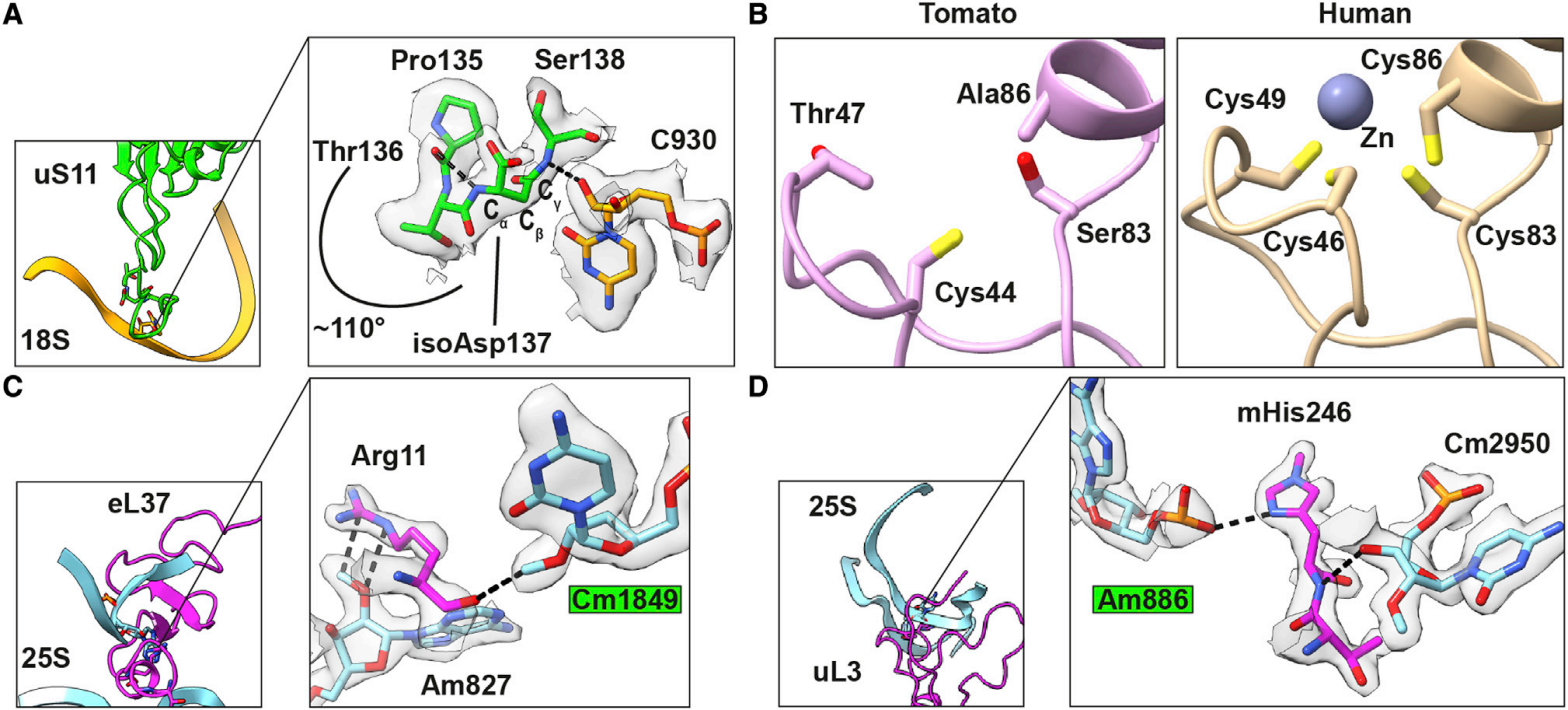
High-resolution features. (A) View of uS11 shows interactions of isoAsp137, Pro135, and Ser138 with rRNA C930 that stabilize the turn in the protein backbone. (B) Comparison of the eL34 zinc finger structure between tomato and human. (C) View of eL37 shows the modified Cm1849 with its methyl group forming a repulsive interaction with the backbone carbonyl group of Arg11, and the modified Gm1857 interacting hydrophobically with Gly6 and Thr6, which stabilizes the helical structure. (D) View of uL3 shows hydrogen bond interactions between the modified Am886, Cm2952, and His236.

As the structure of the ribosome is generally conserved, we focused the analysis on high-resolution features detected in the density. On the protein level, for ribosomal protein uS11, we found an extra density corresponding with a methylene group between C_α_ and the backbone carbonyl group in Asp137, indicating that the γ carboxyl group instead of the α carboxyl group participates in the backbone peptide bond (Watson et al., 2020) (Figure 3A). Hence, the aspartate is converted to isoaspartate (isoAsp) via dehydration and followed by hydrolysis. In the structure, the Ser138 backbone NH hydrogen bonds with the sugar of rRNA C930, and the isoAsp backbone NH hydrogen bonds with the Pro135 backbone carbonyl group, resulting in an approximately 110° turn. A potential role for isoAsp in this position could present during the assembly, as isoAsp residues have previously been proposed to regulate protein activity by a time-dependent molecular switch (Ritz-Timme and Collins, 2002). In *E*. *coli*, the corresponding deamidation of asparagine in uS11 was reported during the logarithmic growth phase on the basis of its ability to serve as an exceptional methyl-accepting protein in cell extracts (David et al., 1999). This post-translational modification can be involved in spontaneously damaged proteins in plants, affecting seed viability, and because studies in *Arabidopsis* showed increased deamidation in response to stress conditions, the reactivity of residues is an important regulator (Peña et al., 2016). As deamidation occurs rapidly *in vitro*, it is difficult to detect under physiological conditions; therefore, the structural approach is informative.

For zinc finger protein eL34, whose aberrant expression in humans is associated with malignancies (Fan et al., 2017), no density corresponding to the zinc ion is found in the plant ribosome structure, and three of four cysteines that form the binding site are absent (Figure 3B; Supplemental Figure 4). In addition, we found a modification in the remaining cysteine 44 (Figure 1A), thus eliminating the zinc finger motif. A phylogenetic analysis showed that the structure-derived observation is conserved in *Viridiplantae* (Supplemental Figure 4). This suggests a lower effect of zinc deficiency on protein synthesis in plants. Our structural data are consistent with the observation that ribosome content remains unchanged in cultured tobacco plant cells with 0.09 ppm zinc in the medium (Obata and Umebayashi, 1988), whereas zinc content in rich soil is estimated at 10–30 ppm. Therefore, the zinc finger previously believed to be universal is absent from plant ribosomes, contributing to physiological functioning of plant metabolism at lower nutrient concentrations.

### rRNA modifications

On the rRNA level, we identified multiple discrepancies between the cryo-EM density map and available sequences (Supplemental Figure 5). Through a combination of analysis of the density map, sequencing, and alignments, we detected 9 deletions, 18 insertions, and 4 mutations in the *S*. *lycopersicum* rRNA (Figure 1B; Supplemental Table 2 and 3), which allowed us to correct the sequence for the model organism (Supplemental Figure 6). To confirm that discrepancies are independent of the plant cultivar, we performed multiple sequence analysis, and only position 120 in the 5S rRNA was found to vary. Next, we analyzed rRNA modifications by the quantitative stable isotope-labeled ribonucleic acid as an internal standard (SILNAS) method (Taoka et al., 2015). 2′-*O*-methylations can change in response to upstream signaling pathways (Jansson et al., 2021). Therefore, to confirm that the identified modifications represent a fundamental feature of the plant ribosome and not an intrinsic modulation, we extracted material from young establishment stages and stressed leaves in two separate experiments (Supplemental Figure 7). The consistent stoichiometry between the experiments suggests that the primary and secondary veins exhibit rRNA modifications similar to those of the lamina. Overall, 216 modifications could be assigned, 71 of which are plant specific, meaning that they are not found in other organisms (Figure 1; Supplemental Tables 4–7). Among all the assigned modifications, 89 are strongly supported by the cryo-EM density map. The position and stoichiometry of all modifications are presented in Supplemental Table 8, and a role could be proposed for some of them. For example, 2′-*O*-methylguanosine 1857 and 2′-*O*-methylcytidine 1849, together with the conserved modification 2′-*O*-methyladenosine 827, shape the N-terminal region of eL37 through hydrophobic interactions (Figure 3C). This region is involved in constructing the peptide exit tunnel. Protein eL37 is required for recruiting Nsa2 and Nog2 for 27SB pre-rRNA processing, and its repression would cause null synthesis of 25S relative to 18S rRNA (Gamalinda et al., 2013). In a similar way, 2′-*O*-methyladenosine 886 and conserved 2′-*O*-methylcytosine 2920 directly interact with the modified residue 3-methylhistidine 246 of uL3 through hydrogen bonding (Figure 3D). This modification is crucial for proper pre-rRNA processing, polysome formation, and correct ribosomal function (Malecki et al., 2021).

**Figure 4:**
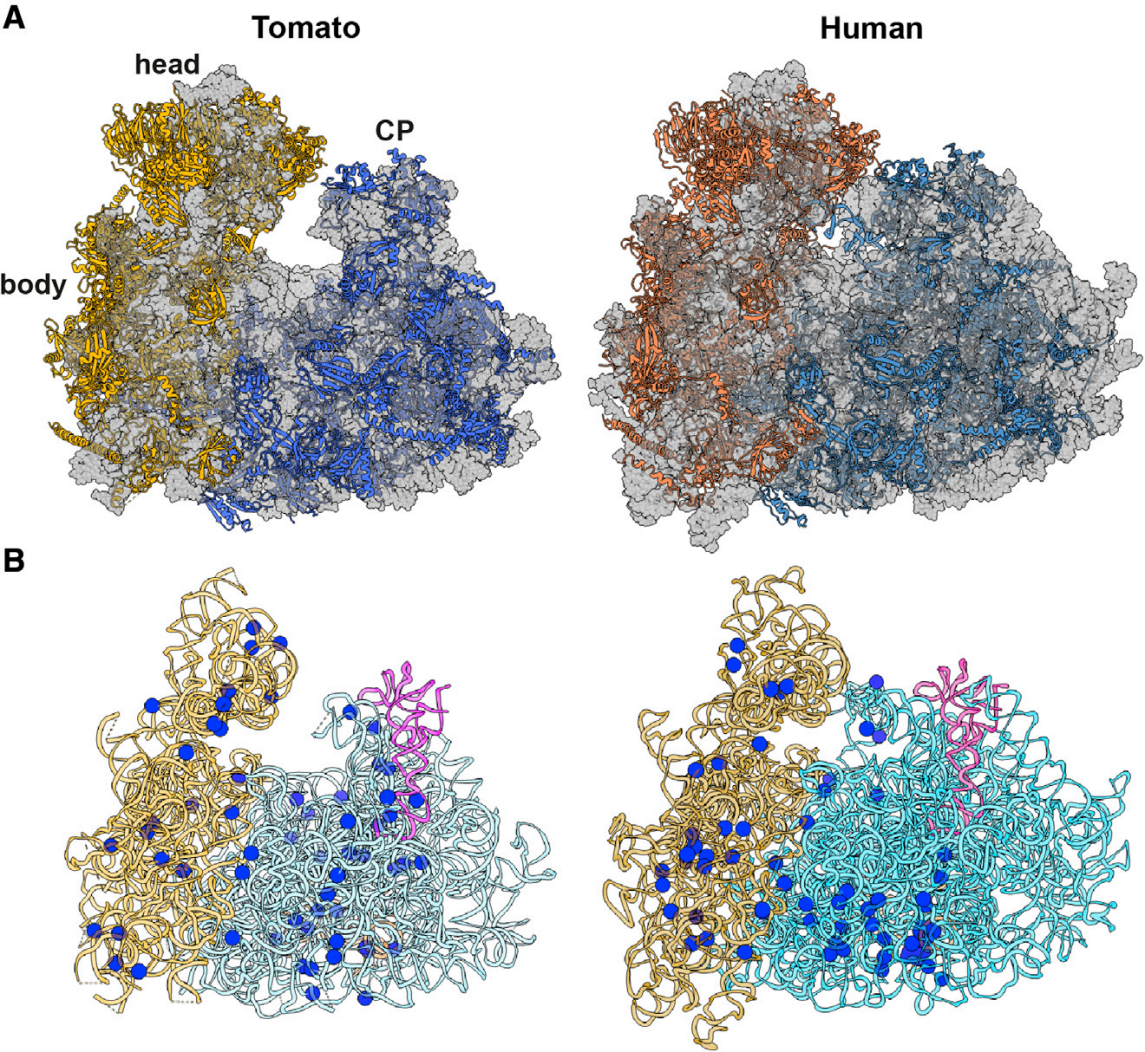
Side-by-side comparison of tomato and human cytosolic ribosomes. (A) The overall structure of tomato (current work) and human (PDB 6QZP; Natchiar et al., 2017) cytosolic ribosomes. (B) The 3D mapping specific modifications.

Because a similar analysis has also been performed for a human ribosome (Taoka et al., 2018), we compared the data (Figure 4, Supplemental Figures 2 and 3, and Supplemental Table 8). The major difference between the ribosomal structures of the two species resides in the length of their rRNA ESs. Because no structure-based 2D rRNA diagrams are available in the literature, we constructed them to expand on the comparison and mapped the modifications (Figure 4B; Supplemental Figures 2 and 3). The plant and human ribosome have a similar number of species-specific rRNA modifications in each of the ribosomal subunits. However, the two subunits differ markedly in the spatial distribution of their species-specific modifications. In the large subunit, most human-specific modifications are concentrated at the bottom, in proximity to the two largest ESs—ES3 and ES6. By contrast, in the small subunit, both plant- and human-specific modifications are scattered all over its surface.

The deposition of chemical modifications into RNA has recently emerged as a source of ribosome heterogeneity and constitutes a mechanism for rapid adaptation to changing environmental cues (Gay et al., 2021). Such adaptation is crucial for cellular homeostasis, and dysregulated RNA modification pathways in humans have been linked to tumorigenesis (Kumari et al., 2021). A recently constructed rRNA 2′O-methylation landscape of primary human breast tumors uncovered the existence of stable and variable modification sites (Marcel et al., 2020). These affect the dynamics of rRNA, resulting in a change in the balance between different conformational states of the ribosomes required for translation (Khoshnevis et al., 2022). Thus, chemical modifications of ribosomal RNA represent a level of gene expression regulation. In this respect, our plant ribosome profiling that maps specific 2′O-methylations provides an additional layer of information to capture ribosomal heterogeneity in plants and offers a new molecular signature of a plant ribosome.

In conclusion, analyses of the plant ribosome provide validated information on the revised sequence of rRNA, ribosomal modifications, and high-resolution structural details with implications for protein synthesis, plant genomics, and ribosome evolution. This work also shows that high-resolution cryo-EM in combination with mass spectrometry can be used as a tool for detecting the formation of physiologically induced chemical modifications and for uncovering plant-specific features of translation. Together, these data provide a reference model of a plant ribosome for use in structural studies and an accurate source of information for biodiversity researchers.

## Materials and methods

### Sample preparation for cryo-EM

Tomato plants (*S*. *lycopersicum*, variety Rutgers) were cultivated in a growth chamber with 16 h fluorescent light at 30°C and 8 h darkness at 25°C. To stimulate stress conditions, plants were infected with citrus exocortis viroid by puncture (Bellés et al., 1991). Leaf tissue was collected from the apexes and frozen in liquid nitrogen. The material was then milled with a RESTCH Mixer Mill and stored at –80°C. Upon thawing, 2 mL of buffer containing 0.3 M mannitol, 20 mM HEPES-KOH (pH 7.5), 50 mM KCl, 5.0 mM Mg(OAc)_2_, 1.0 mM DTT, EDTA-free Protease Inhibitor Cocktail (Roche), and RNaseOUT (Invitrogen) was added to 1 g of the material, and the sample was pipetted 20 times to extract the cytoplasmic fraction. After centrifugation at 1000×*g* for 2 min at 4°C, the supernatant was collected and re-centrifuged at 18,000×*g* for 10 min at 4°C to pellet debris and organelles. The supernatant was then carefully loaded onto a sucrose cushion (0.6 M sucrose, 0.50% Triton X-100, 10 mM HEPES-KOH [pH 7.5], 50 mM KOAc, 5.0 mM Mg(OAc)_2_, 1.0 mM DTT) and centrifuged at 355,040×*g* with a TLA 120.2 rotor (Beckman) for 1 h at 4°C. The pellet was washed with resuspension buffer containing 10 mM HEPES-KOH (pH 7.5), 50 mM KOAc, 5.0 mM Mg(OAc)_2_, and 1.0 mM DTT before resuspension. An additional centrifugation step at 18,000×*g* for 10 min at 4°C was performed to pellet aggregates and debris. The supernatant was loaded onto a 15%–30% sucrose gradient and centrifuged at 101,104×*g* with a TLS-55 rotor (Beckman) for 2.25 h at 4°C. The gradient was fractionated, the peak of the 80S ribosome was pooled, and the sample was transferred to resuspension buffer and concentrated by ultrafiltration with a Vivaspin MWCO 30,000 (Sartorius). Ribosome concentration was adjusted using A_260_ to approximately 10–20 units for cryo-EM. Grids (Quantifoil R2/1, 300 mesh, gold) were manually coated with a continuous carbon film of 3-nm thickness. Glow discharge was performed for 30 s at 20-mA intensity. Sample vitrification was performed with a Vitrobot MKIV (FEI/Thermo Fisher) with 100% humidity at 4°C with a 30-s wait time and a 3.5-s blotting time. We loaded 3 μL of the sample onto the grids.

### Data collection and processing

Data were collected on a Titan Krios microscope (Thermo Fisher Scientific) operated at 300 kV with a Quantum K2 Summit camera (Gatan) using EPU software at a pixel size of 0.82–0.83 Å. Movies with 20 frames were collected with a total dose of 30.2 e^−^/Å^2^ and an exposure time of 4 s at a defocus range of 1–2.8 μm with a 0.2-μm step. Three datasets were collected, and each was processed separately in RELION 3.0.8 (Zivanov et al., 2018). Motion correction was performed with MotionCor2 (Zheng et al., 2017), and the CTF parameters were estimated by Gctf (Zhang, 2016). The particles were initially picked either by Gaussian-based auto-picking in RELION or Gautomatch v0.55 and subjected to two-dimensional (2D) classification. Selected 2D class averages were used as templates for reference-based picking, followed by 2D classification in RELION 3.0.8. After several rounds of optimizations, the last picking was performed with gautomatch v0.55, obtaining 386,768 particles from dataset 1; 298,277 from dataset 2; and 329,530 from dataset 3. The final picked particles were extracted with four-time binning for the 2D classification, which was performed iteratively; the best classes were retained, and the ribosome-looking classes were further 2D classified to obtain the best classes and eventually merge them together. The merged particles were re-extracted with twice binning for 3D auto-refinement by RELION 3.0.8, followed by 3D classification to remove broken particles. We selected 106,303 good particles from dataset 1, 48,545 from dataset 2, and 31,991 from dataset 3. Selected particles were re-extracted without binning, and 3D auto-refinement was performed to create a solvent mask. We performed 3D auto-refinement using the solvent mask for the final reconstruction of each dataset; namely, 3 Å of resolution from dataset 1, 3.3 Å from dataset 2, and 3.45 Å from dataset 3. At this point, the datasets were merged, and the processing continued in RELION 3.1 (Zivanov et al., 2020). The merged particles were 3D auto-refined using the map that resulted from dataset 1 as a reference; RELION 3.1 therefore resampled the processed particles according to the reference map used (i.e., 0.83 Å/pixel), followed by CTF refinement and Bayesian polishing. Finally, an additional step of CTF refinement was performed, followed by a 3D auto-refine and post-processing, obtaining 2.38-Å resolution with a sharpening *B*-factor of –47.277.

To improve local resolution, local masks covering 60S, 40S-body, and 40S-head were prepared. First, local-masked 3D auto-refine covering 60S was performed for the best map of 60S. Particle subtraction applying a loose mask around the 40S was performed to subtract signals outside of the mask, followed by local-masked 3D auto-refine using the 40S-body mask to obtain the best map of the 40S body. Finally, further particle subtraction applying the 40S-head mask to retain only the signal of the head was performed, followed by local-masked 3D auto-refine covering the head for its best map. To separate tRNA-bound states, an unmasked 3D classification with local-angular search from the overall 3D auto-refine was performed to separate the rotated and non-rotated conformations. A mask covering the tRNA binding sites was prepared. For each rotational state, signal subtraction applying the tRNA mask was performed to subtract outside the mask, followed by a focused 3D classification without alignment using the tRNA mask. Two tRNA-bound elongation states, A/A-P/P-E/E (36,183 particles) and A/P-P/E (66,681 particles), were obtained.

To classify the rRNA ES ES27, particles from the local-masked 60S alignment were further signal subtracted, retaining the signals inside a mask englobing the three different possible conformations found. Then, a focused 3D classification in that region with optimized parameters, τ value 150, E-step 12 Å, and 25 iterations gave three distinct classes. The signal was reverted to obtain the complete ribosome, and a 3D auto-refine was performed to create a proper solvent mask for each class, followed by a final 3D auto-refine using the solvent mask giving the best map of each class. For each of the maps, local resolution filtering using RELION 3.1 was performed, applying the sharpening *B*-factor estimated by post-processing.

### Model building and refinement

Model building was performed in Coot (Emsley and Cowtan, 2004). The starting model used as a reference was the *H*. *sapiens* ribosome (PDB: 6EK0) (Natchiar et al., 2017). For every protein, a BLASTp search was performed at both the NCBI and Sol Genomics Network databases in case any incongruences were found. Then, each protein chain in 6EK0 PDB was used as a template in SWISS-MODEL (Waterhouse et al., 2018) to obtain the homology model for tomato. Protein and RNA models were fitted and manually adjusted to the cryo-EM maps. Ligands, metal ions, waters, and modifications were placed based on the density. Hydrogens were generated to have better clash scores, and stereochemical and *B*-factor refinements were performed using phenix.real_space_refine in the PHENIX suite. The final models were validated using MolProbity (Williams et al., 2018). Refinement statistics are given in Supplemental Table 1.

### Total RNA extraction

Leaf tissue was collected, frozen, and milled as mentioned above. Total RNA was purified by adding 5 volumes, relative to the sample volume, of home-made Trizol (38% phenol saturated [pH 4.3], 0.8 M guanidine thiocyanate, 0.8 M ammonium thiocyanate, 0.1 M sodium acetate [pH 5.0], 5% glycerol) and vigorously vortexed for 10–30 s. Incubation for 5 min at room temperature was followed by the addition of 1 volume of chloroform, relative to the original sample volume, and the contents were mixed by inverting the tube 10–20 times. The mixture was then centrifuged at 12,000×*g* for 15 min at 4°C, and the aqueous phase was transferred to a new tube. At this point, 1.1 volumes, relative to the aqueous phase, of cold isopropanol were added and mixed by gently inverting the tube. After incubating for 2 h at −20°C, the RNA was pelleted at 12,000×*g* for 45 min at 4°C. After the supernatant was discarded, 1 volume, relative to the aqueous phase, of cold 70% ethanol was added to gently clean the RNA. After a 10-min incubation at 4°C, the tube was centrifuged at 12,000×*g* for 10 min at 4°C, and the supernatant was discarded. Finally, the pellet was allowed to dry at 4°C and resuspended with 35 μL of RNase-free water.

### Multiple sequence alignment, phylogenetic tree, and figures

The multiple sequence alignments were performed with ClustalW and the phylogenetic tree reconstruction with MEGA7 (maximum likelihood method with 1000 bootstrap replications, gap opening penalty of 2) (Kumar et al., 2016). All the sequences were retrieved from the first BLASTp hit at NCBI, except for that of *Picea abies*, which was only possible to find at “congenie.org” (Nystedt et al., 2013). The sequences used for multiple sequence alignment and phylogenetic tree construction are found under their respective figures. All figures were prepared with ChimeraX (Goddard et al., 2018) and Coot (Emsley and Cowtan, 2004).

### Sequencing of 18S and 25S rRNAs

Single-stranded cDNA was synthesized from the total RNA using a cDNA synthesis kit (SuperScript III First-Strand Synthesis System, Thermo Fisher Scientific) with the primer Tmt18S_R1764 or Tmt25S_R3367 (Supplemental Table 9). The cDNA was amplified by PCR using the single-stranded cDNA as a template and synthetic oligonucleotides as primers (Supplemental Table 9). The PCR product was directly sequenced by the Sanger method using the sequencing primers (Supplemental Table 9).

### rRNA purification

The total RNA was applied to a reversed-phase LC column (PLRP-S 4000 Å, 4.6 × 150 mm, 10 μm, Agilent Technologies), and the rRNAs were eluted with a 120-min linear gradient of 10.8%–13.2% (v/v) acetonitrile in 100 mM TEAA (pH 7.0) and 0.1 mM diammonium phosphate at a flow rate of 200 μL/min at 60°C while measuring the eluate at A260 (Yamauchi et al., 2013). The 5.8S, 18S, and 25S rRNA fractions of the eluate were used directly for the liquid chromatography/tandem mass spectrometry (LC-MS) analysis. The 5S rRNA fraction was further purified by reversed-phase LC using a column (PLRP-S 300 Å, 2.1 × 200 mm, 3 μm, Agilent Technologies) with a 120-min linear gradient of 11.8%–14.2% (v/v) acetonitrile in 100 mM TEAA (pH 7.0) and 0.1 mM diammonium phosphate at a flow rate of 100 μL/min at 60°C.

### Sequence-specific RNase H cleavage of rRNA

The purified rRNA (1 pmol) was digested with 5 U RNase H (Takara Bio) at 42°C for 1 h, guided by synthetic RNA/DNA hybrids complementary to the duplex cleavage sites (5 pmol, Supplemental Table 9) in 20 μL of 40 mM Tris–HCl (pH 7.7), 0.25 mM MgCl_2_, 1 mM DTT, and 4% glycerol. Before adding the enzyme, the sample was denatured at 65°C for 10 min. The reaction was stopped by adding 0.5 μL of 0.1 M EDTA, and the resulting fragments were separated by polyacrylamide gel containing 8 M urea. The gel was stained with SYBR Gold (Invitrogen) for 1 min, and the gel pieces containing RNA bands were excised from the gel and cut into small pieces. The RNA fragment was extracted by soaking the gel pieces in 80 μL of 20 mM triammonium citrate containing 4 M urea for 1 h. The extraction was carried out two times, and the extracts were combined and passed through a centrifugal filter unit equipped with a polyvinylidene fluoride membrane (Ultrafree-MC, Millipore). The RNAs in the eluate were finally purified by reversed-phase LC on a PLRP-S 300 Å column (2.1 × 100 mm, 3 μm, Agilent Technologies) as described previously (Yamauchi et al., 2013; Taoka et al., 2010).

### LC-MS, MS/MS, and MS/MS/MS analysis and database search of RNA fragments

The rRNA was digested with RNase T1 (Worthington) or A (Sigma-Aldrich) as described previously (Taoka et al., 2010). The nucleolytic RNA fragments were analyzed with a direct nanoflow LC-MS system as described previously (Nakayama et al., 2015). The LC eluate was sprayed online at –1.3 kV with the aid of a spray-assisting device (Nakayama et al., 2019) to a Q Exactive Plus mass spectrometer (Thermo Fisher Scientific) in negative ion mode. Other settings for MS, MS/MS, and MS/MS/MS were as described previously (Yamauchi et al., 2016; Nakayama et al., 2019). Ariadne was used for database searches and assignment of MS/MS RNA spectra (Nakayama et al., 2009). A composite of *S*. *lycopersicum* cytosolic (5S, 5.8S, 18S, and 25S) and chloroplastic (4.5S, 5S, 16S, and 23S) rRNA sequences was used as a database. The following default search parameters for Ariadne were used: maximum number of missed cleavages, 2; variable modification parameters, 3 modifications, including monomethylation, dimethylation, acetylation, and methylaminocarboxypropylation per RNA fragment for any residue; RNA mass tolerance, ±5 ppm; and MS/MS tolerance, ±20 ppm.

### Internal standard RNAs and SILNAS-based quantitation of the stoichiometry of post-transcriptional modification

The plasmids encoding tomato 18S and 25S rRNAs with the T7 promotor were purchased from Twist Bioscience. To synthesize RNA, 2 μg of the plasmid DNA was linearized with NotI and transcribed using a MEGAscript T7 kit (Thermo Fisher Scientific). When RNA was synthesized, guanosine-^13^C_10_ 5′-triphosphate (Sigma-Aldrich), cytidine-^13^C_9_ 5′-triphosphate, or uridine-^13^C_9_ 5′-triphosphate (Santa Cruz Biotechnology) solution was used instead of the respective 5′-triphosphate reagent that contained carbons with a natural isotope distribution. The RNA was precipitated in ethanol, solubilized in nuclease-free water, and purified further by reversed-phase LC as described above. SILNAS-based quantitation was performed as described previously (Taoka et al., 2018; Nakayama et al., 2009). In brief, RNA (approximately 100 fmol) from natural sources with a natural isotope distribution was mixed with an equal amount of synthetic RNA transcribed *in vitro* with ^13^C-labeled guanosine and digested with RNase T1. For the RNA transcribed with ^13^C_9_-labeled cytidine and uridine, RNase A was used as the digestion enzyme. The 1:1 RNA mixing was performed based on measurement of the absorbance at 260 nm and ensured later by a correction factor obtained experimentally. After obtaining the LC-MS spectrum of the digested RNA mixture, the stoichiometry of RNA modification at each site was estimated by the Ariadne program designed for SILNAS (Taoka et al., 2018). The results were confirmed by manual inspection of the original MS spectrum to examine whether the estimates were based on uncontaminated MS signals (Supplemental Table 8). The masses of RNA fragments and a-, c-, w-, and y-series ions were calculated with Ariadne (http://ariadne.riken.jp/). The cyanoethylation method was used for pseudouridine identification in the rRNAs.

### Generation of structure-derived rRNA 2D diagrams

The secondary structure diagrams for *S*. *lycopersicum* was initially generated using the template-based approach implemented in the R2DT webserver (Sweeney et al., 2021). The 60S and 40S rRNAs were templated from layouts of *S*. *cerevisiae* provided by RiboVision (Bernier et al., 2014). The canonical Watson–Crick base pairs were extracted from the experimental 3D structures described in the current study using the DSSR package (Lu et al., 2015). The layouts were manually adjusted, accounting for the 3D derived base pairing, using XRNA-GT https://github.com/LDWLab/XRNA-GT. The secondary structure diagrams for *H*. *sapiens* were obtained from RiboVision. For both *S*. *lycopersicum* and *H*. *sapiens* secondary structure diagrams, the data mapping for the ESs and experimentally detected post-transcriptional modifications was performed in RiboVision. The final adjustments to the layouts (labeling and annotations) were performed in Adobe Illustrator.

## Funding

This work was supported by the Swedish Foundation for Strategic Research (ARC19:0051), the 10.13039/501100004063Knut and Alice Wallenberg Foundation (2018.0080), the EMBO Young Investigator Program, and a 10.13039/100000104NASA award (80NSSC18K1139 to A.S.P.).

## Author contributions

P.C. and Y.I. purified the sample, collected cryo-EM data, and built the model under the supervision of A.A.; Y.N. and M.T. performed mass spectrometry and data analysis; A.S.P. constructed RNA diagrams; P.L. supervised the project; P.C., Y.I., and A.A. wrote the manuscript.

## Data and code availability

All data produced or analyzed in this study are included in the main text or the supplementary materials. The cryo-EM density maps and atomic coordinates have been deposited in the Electron Microscopy Data Bank (EMDB) and Protein Data Bank (PDB) under accession numbers EMD-14001, EMD-14002, EMD-14003, EMD-14004, EMD-32404, EMD-14051, and EMD-14052 and 7QIW, 7QIX, 7QIY, and 7QIZ. The rRNA sequence FASTA files have been deposited in GenBank under accession numbers OK073662 for 25S, OK073663 for 18S, OK073664 for 5.8S, and OK073665 for 5S.

## Supporting citations

Additional references in Supplemental information: Itoh et al., 2022a; Itoh et al., 2022b.

## Supplemental information

Document S1. Supplemental Figures 1–7 and Supplemental Tables 1–7, 9

Supplemental Table 8. Positions and types of PTMs in *S. lycopersicum* rRNAs identified by LC-MS

Document S2. Article plus supplemental information
