# Supplementary material for: Cryo-EM structure and rRNA modification sites of a plant ribosome
Source: Plant Commun. 2022 May 27;3(5):100342. doi: 10.1016/j.xplc.2022.100342 (PMC9483110; doi:10.1016/j.xplc.2022.100342)
Supplement: Document S1. Supplemental Figures 1–7 and Supplemental Tables 1–7, 9 [file mmc1.pdf]

Plant Communications, Volume *3*

Supplemental information

Cryo-EM structure and rRNA modification sites of a plant ribosome

Patrick Cottilli, Yuzuru Itoh, Yuko Nobe, Anton S. Petrov, Purificación Lisón, Masato Taoka, and Alexey Amunts

Supplemental Information:

Cryo-EM structure and rRNA modification sites of a plant ribosome

Patrick Cottilli[1†], Yuzuru Itoh[1†], Yuko Nobe[2†], Anton S. Petrov[3], Purificación Lisón[4], Masato Taoka[2*], Alexey Amunts[1*]

[1] Science for Life Laboratory, Department of Biochemistry and Biophysics, Stockholm University, 17165 Solna, Sweden.

[2] Department of Chemistry, Graduate School of Science, Tokyo Metropolitan University, Minami-osawa 1-1, Hachioji-shi, Tokyo 192-0397, Japan.

[3] School of Chemistry and Biochemistry, Georgia Institute of Technology, Atlanta, GA.

[4] Instituto de Biología Molecular y Celular de Plantas. Universitat Politècnica de València (UPV) – Consejo Superior de Investigaciones Científicas (CSIC). Ciudad Politécnica de la Innovación (CPI), Valencia 46022, Spain.

† These authors contributed equally to this work.

* To whom correspondence should be addressed: mango@tmu.ac.jp, amunts@scilifelab.se

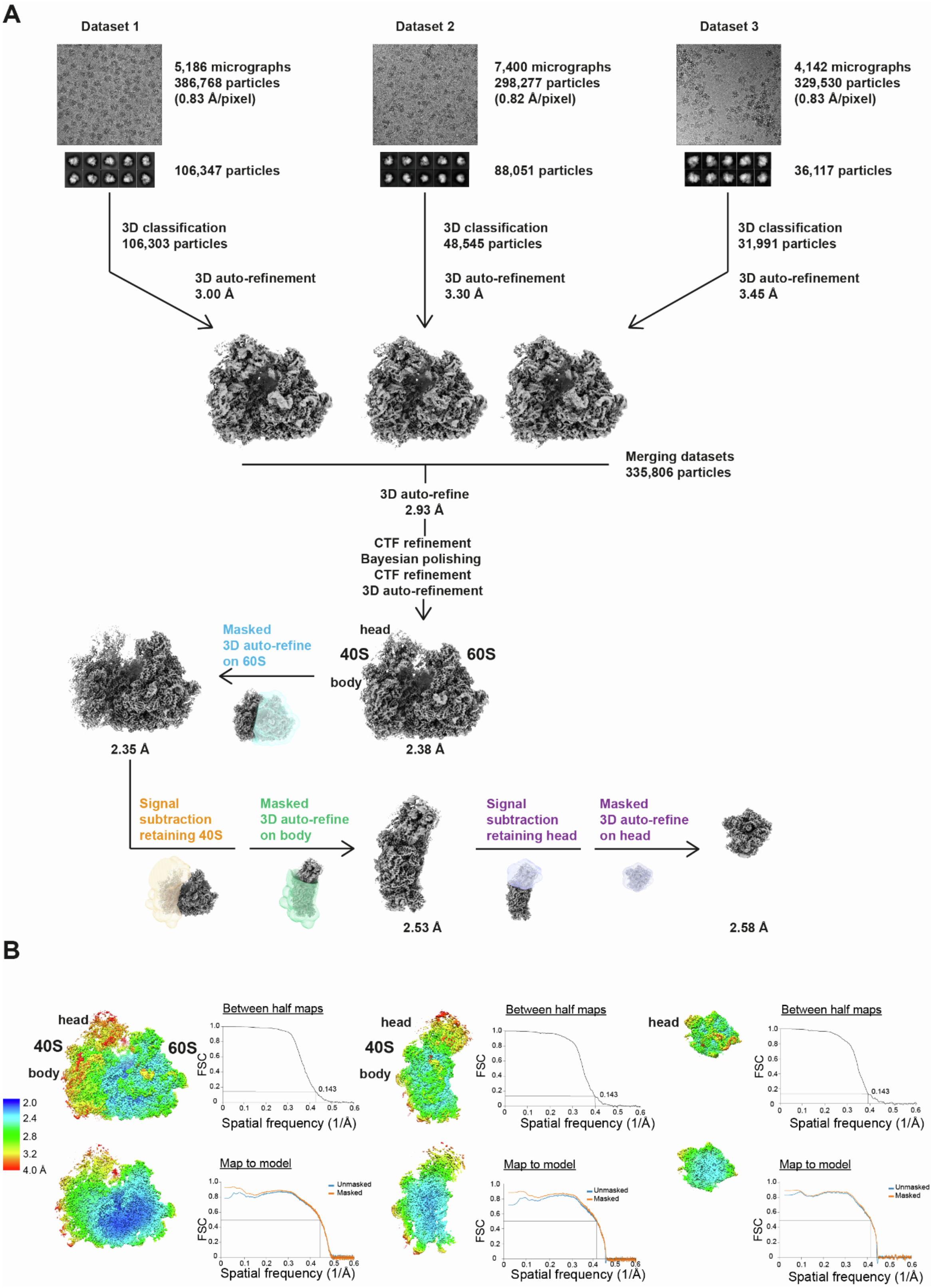

Supplementary Figure S1: Cryo-EM data collection and processing. (a) Processing scheme for the 60S, the 40S body and head. Representative micrographs and 2D class averages are shown. (b) Maps are colored by local resolution, isosurface (upper) and cut through (lower). Fourier shell correlation (FSC) plot between two half maps (threshold 0.143) and that of map-to-model (threshold 0.50) are shown for each map. The representative micrographs reflect prep-to-prep variability.

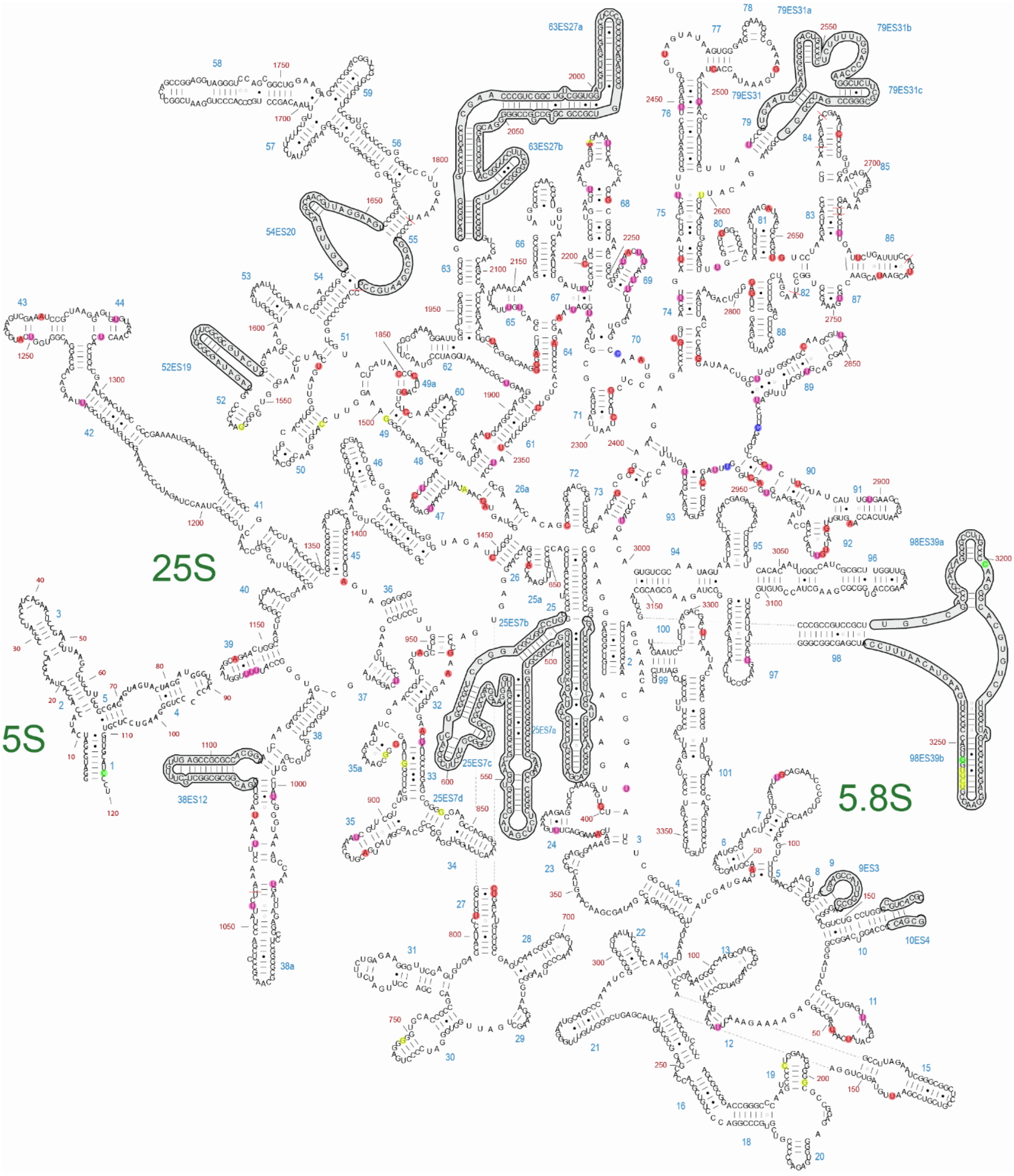

25S
5S
5.8S

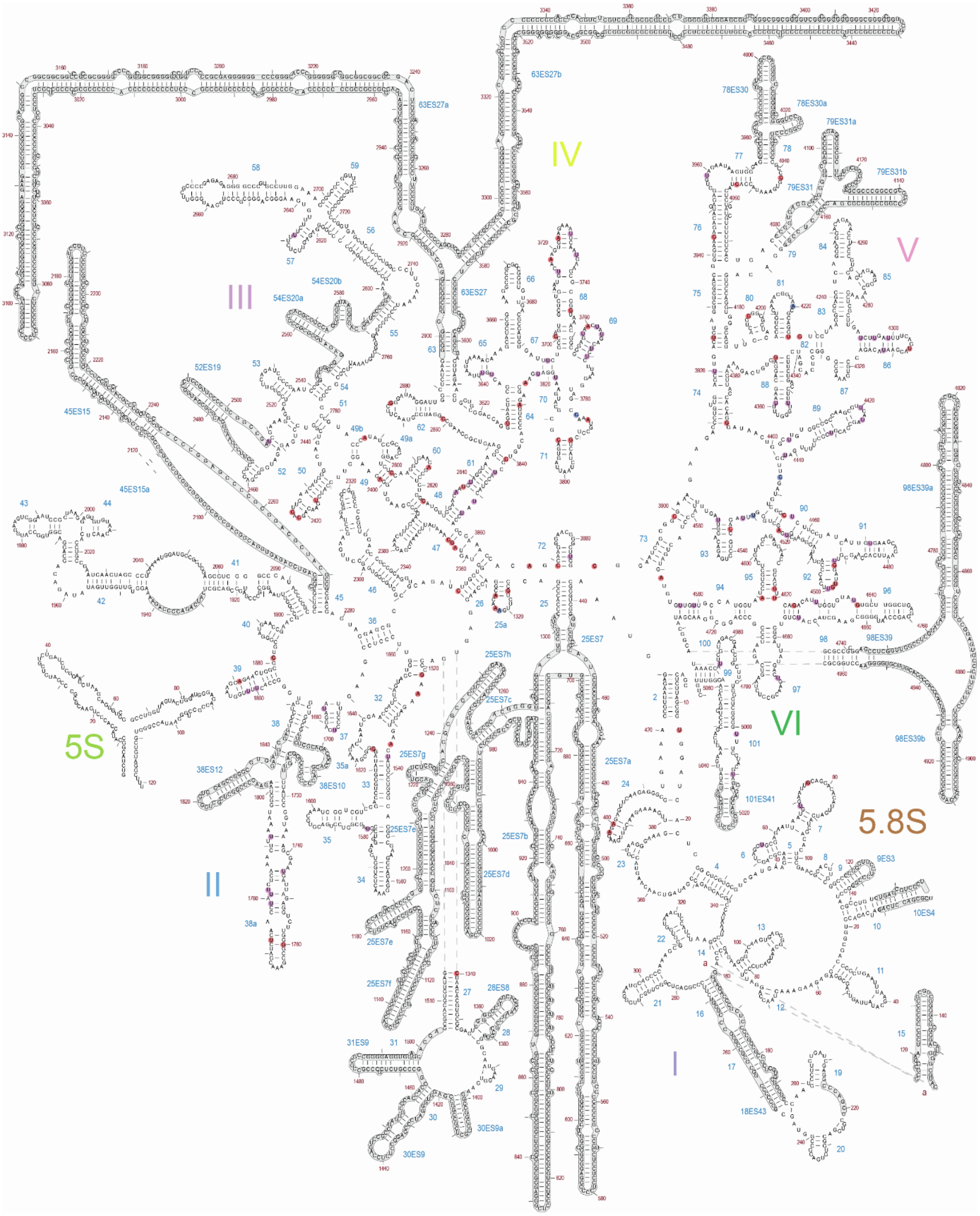

Supplementary Figure S2: ***S. lycopersicum*** **(top) and** ***H. sapiens*** **(bottom) structure-derived rRNA 2D diagrams for the 25S rRNA.** Base-pair interactions of canonical Watson-Crick pairs, standard G-U pairs and other local non-canonical ones are marked as dashes, black bullets and white bullets, respectively. Expansion segments are encircled and highlighted with grey. 2′-*O*-methylations are red, base modification blue, pseudouridines magenta, insertions yellow, point mutations green, and deletions are red lines.

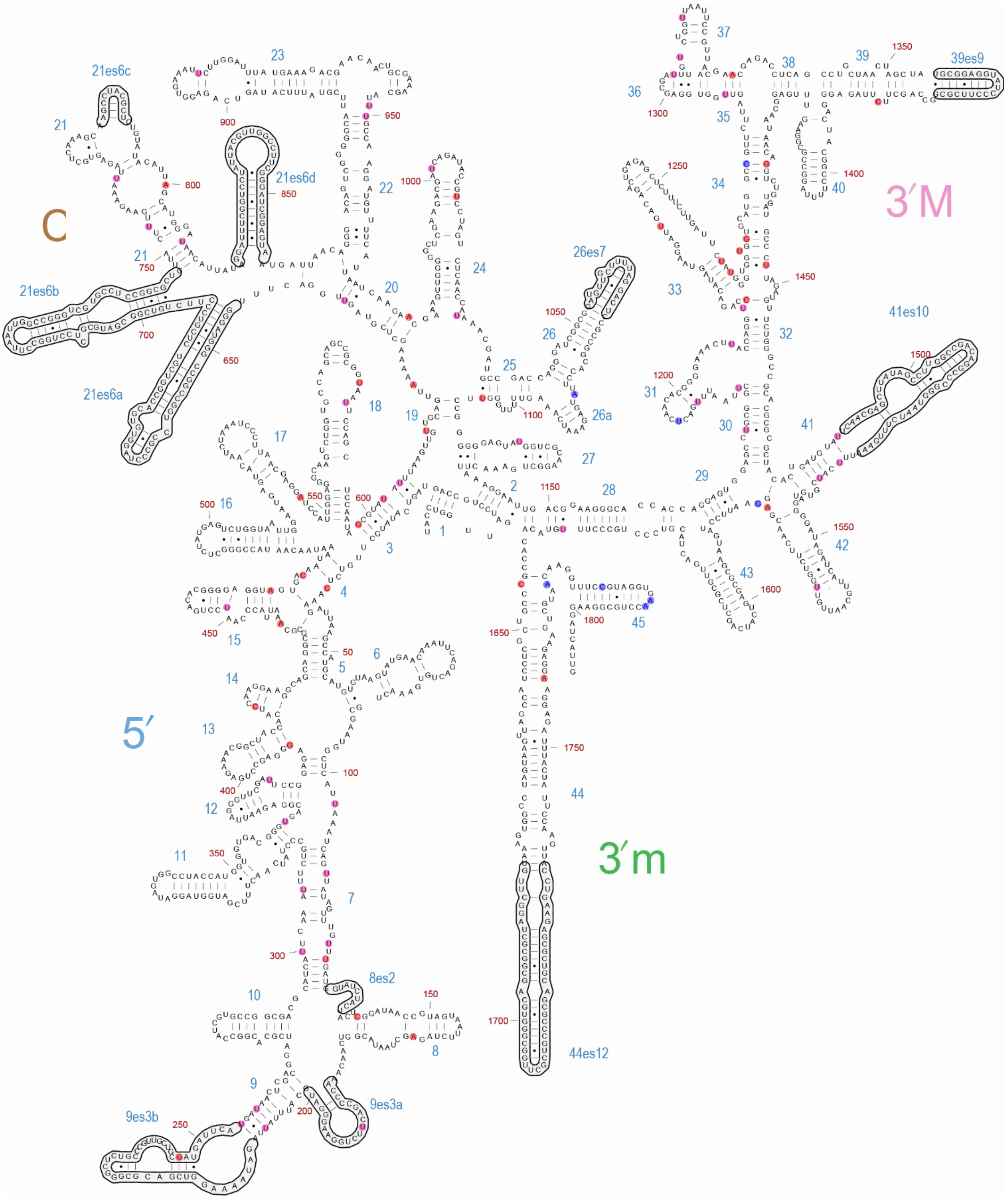

C
5′
3′M
3′m
21es6a
21es6b
21es6c
21es6d
26es7
26a
39es9
41es10
44es12
8es2
9es3a
9es3b

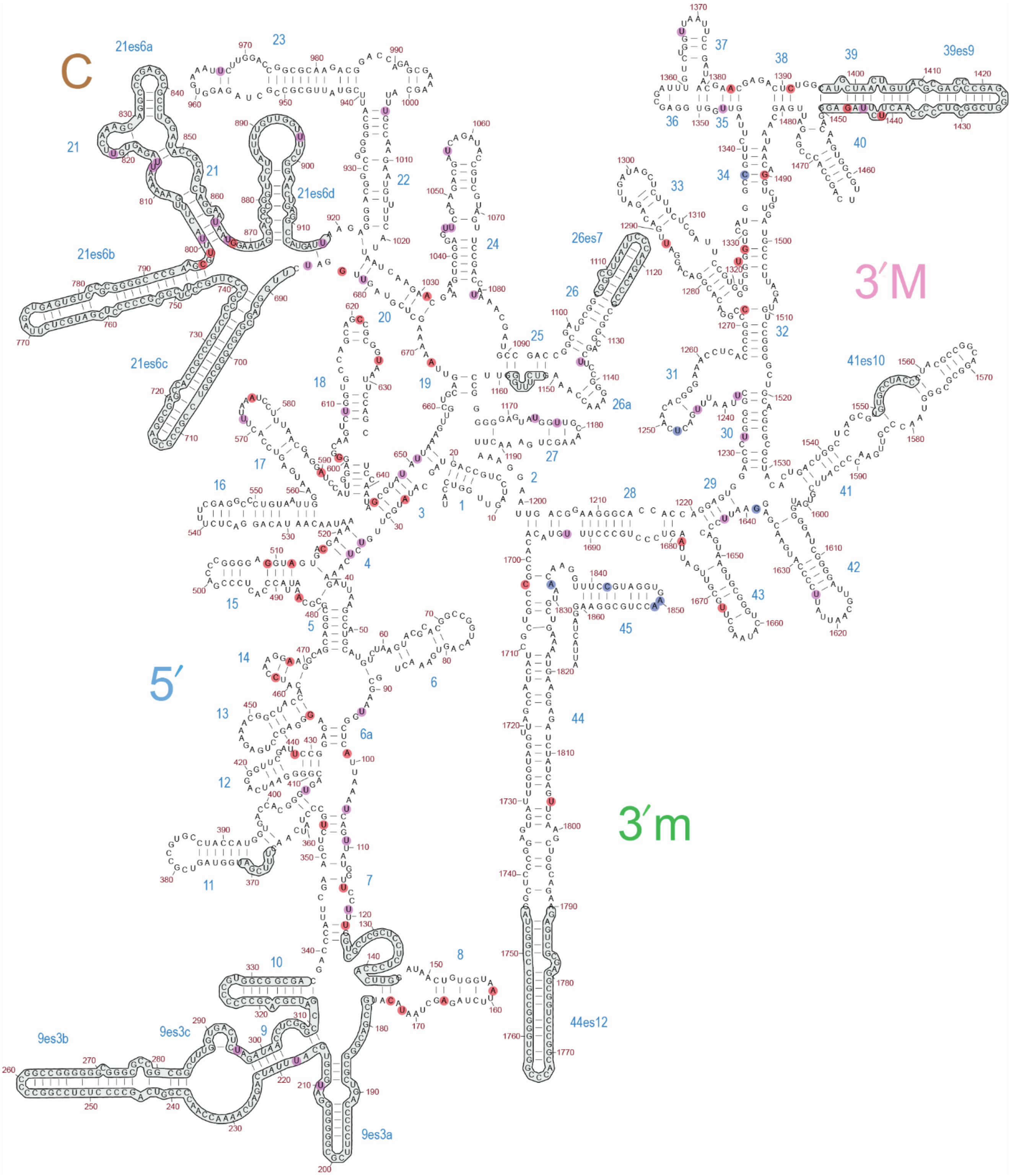

Supplementary Figure S3: ***S. lycopersicum*** **(top) and** ***H. sapiens*** **(bottom) structure-derived rRNA 2D diagrams for the 18S rRNA.** Base-pair interactions of canonical Watson-Crick pairs, standard G-U pairs and other local non-canonical ones are marked as dashes, black bullets and white bullets, respectively. Expansion segments are encircled and highlighted with grey. 2′-*O*-methylations are red, base modification blue, pseudouridines magenta, insertions yellow, point mutations green, and deletions are red line

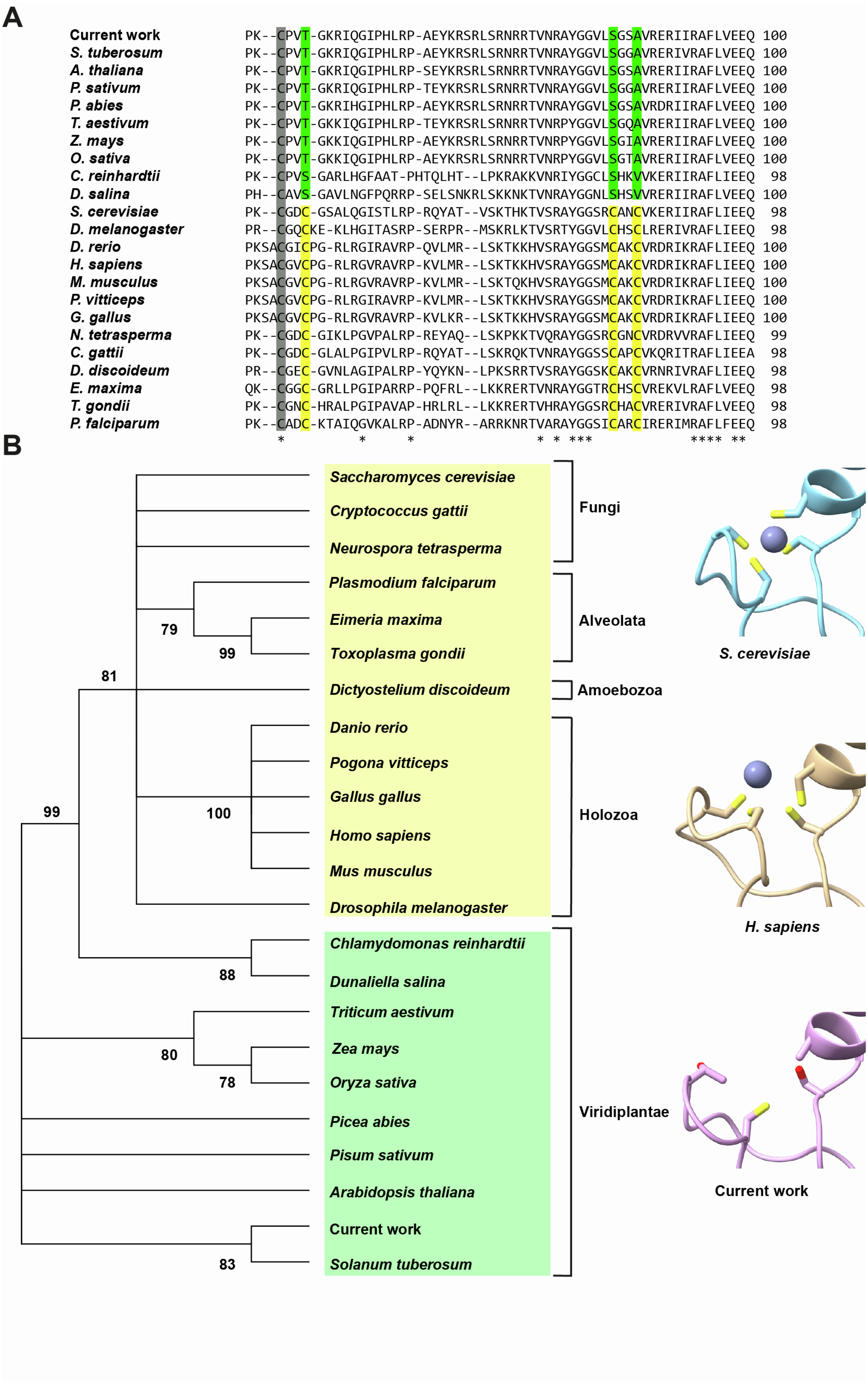
A
Current work PK--CPVT-GKRIQGIPHLRP-AEYKRSRLSRNRRTVNRAYGGVLSGSAVRERIIRAFLVEEQ 100
S. tuberosum PK--CPVT-GKRIQGIPHLRP-AEYKRSRLSRNRRTVNRAYGGVLSGGAVRERIVRAFLVEEQ 100
A. thaliana PK--CPVT-GKRIQGIPHLRP-SEYKRSRLSRNRRTVNRAYGGVLSGSAVRERIIRAFLVEEQ 100
P. sativum PK--CPVT-GKRIQGIPHLRP-TEYKRSRLSRNRRTVNRAYGGVLSGGAVRERIIRAFLVEEQ 100
P. abies PK--CPVT-GKRIHGIPHLRP-AEYKRSRLSRNRRTVNRAYGGVLSGSAVRDRIIRAFLVEEQ 100
T. aestivum PK--CPVT-GKKIQGIPHLRP-TEYKRSRLSRNRRTVNRPYGGVLSGQAVRERIIRAFLVEEQ 100
Z. mays PK--CPVT-GKKIQGIPHLRP-AEYKRSRLSRNRRTVNRPYGGVLSGIAVRERIIRAFLVEEQ 100
O. sativa PK--CPVT-GKKIQGIPHLRP-AEYKRSRLSRNRRTVNRPYGGVLSGTAVRERIIRAFLVEEQ 100
C. reinhardtii PK--CPVS-GARLHGFAAT-PHTQLHT--LPKRAKKVNRIYGGCLSHKVVKERIIRAFLIEEQ 98
D. salina PH--CAVS-GAVLNGFPQRRP-SELSNKRLSKKNKTVNRAYGGNLSHSVVRERIIRAFLVEEQ 100
S. cerevisiae PK--CGDC-GSALQGISTLRP-RQYAT--VSKTHKTVSRAYGGSRCANCVKERIIRAFLIEEQ 98
D. melanogaster PR--CGQCKE-KLHGITASRP-SERPR--MSKRLKTVSRTYGGVLCHSCLRERIVRAFLIEEQ 98
D. rerio PKSACGICPG-RLRGIRAVRP-QVLMR--LSKTKKHVSRAYGGSMCAKCVRDRIKRAFLIEEQ 100
H. sapiens PKSACGVCPG-RLRGVRAVRP-KVLMR--LSKTKKHVSRAYGGSMCAKCVRDRIKRAFLIEEQ 100
M. musculus PKSACGVCPG-RLRGVRAVRP-KVLMR--LSKTQKHVSRAYGGSMCAKCVRDRIKRAFLIEEQ 100
P. vitticeps PKSACGVCPG-RLRGIRAVRP-KVLMR--LSKTKKHVSRAYGGSMCAKCVRDRIKRAFLIEEQ 100
G. gallus PKSACGVCPG-RLRGVRAVRP-KVLKR--LSKTKKHVSRAYGGSMCAKCVRDRIKRAFLIEEQ 100
N. tetrasperma PK--CGDC-GIKLPGVPALRP-REYAQ--LSKPKKTVQRAYGGSRCGNCVRDRVVRAFLIEEQ 99
C. gattii PK--CGDC-GLALPGIPVLRP-RQYAT--LSKRQKTVNRAYGGSSCAPCVKQRITRAFLIEEA 98
D. discoideum PR--CGEC-GVNLAGIPALRP-YQYKN--LPKSRRTVSRAYGGSKCAKCVRNRIVRAFLIEEQ 98
E. maxima QK--CGGC-GRLLPGIPARRP-PQFRL--LKKRERTVNRAYGGTRCHSCVREKVLRAFLVEEQ 98
T. gondii PK--CGNC-HRALPGIPAVAP-HRLRL--LKKRERTVHRAYGGSRCHACVRERIVRAFLVEEQ 98
P. falciparum PK--CADC-KTAIQGVKALRP-ADNYR--ARRKNRTVARAYGGSICARCIRERIMRAFLFEEQ 98
* * * * * *** **** **
B
Saccharomyces cerevisiae
Cryptococcus gattii
Neurospora tetrasperma
Fungi
Plasmodium falciparum
Eimeria maxima
Toxoplasma gondii
Alveolata
79
99
81
Dictyostelium discoideum
Amoebozoa
Danio rerio
Pogona vitticeps
Gallus gallus
Homo sapiens
Mus musculus
Drosophila melanogaster
Holozoa
99
100
Chlamydomonas reinhardtii
Dunaliella salina
88
Triticum aestivum
Zea mays
Oryza sativa
80
78
Picea abies
Pisum sativum
Arabidopsis thaliana
Current work
Solanum tuberosum
83
Viridiplantae
S. cerevisiae
H. sapiens
Current work

Protein eL34 sequences used for the tree reconstruction:

ORGANISM	SEQUENCE
Solanum tuberosum	XP_006354783.1
Arabidopsis thaliana	NP_174010.1
Pisum sativum	P40590.1
Picea abies	MA_175574g0010
Clhamydomonas reinhardtii	XP_001697940.1
Dunaliella salina	KAF5843135.1
Saccharomyces cerevisiae	NP_010977.2
Drosophila melanogaster	AAL49199.1
Danio rerio	NP_957416.1
Homo sapiens	P49207
Mus musculus	NP_001005859.1
Pogona vitticeps	XP_020652019.1
Gallus gallus	NP_001268429.1
Cryptococcus gattii	XP_003191786.1
Dictyostelium discoideum	XP_637722.1
Eimeria máxima	XP_013335039.1
Toxoplasma gondii	XP_002366393.1
Plasmodium falciparum	XP_001349019.1

Supplementary Figure S4: Phylogenetic analysis of eL34 zinc finger. (a) Multiple sequence of the zinc finger region. The conserved cysteine is highlighted in gray, three complementary cysteines forming the zinc finger in yellow, and the corresponding *Viridiplantae* residues in green. (b) Maximum likelihood tree condensed at 70%. The clades with the conserved zinc finger are highlighted in yellow, *Viridiplantae* clade in green. Representative structures of the zinc finger or the corresponding part are shown on the right.

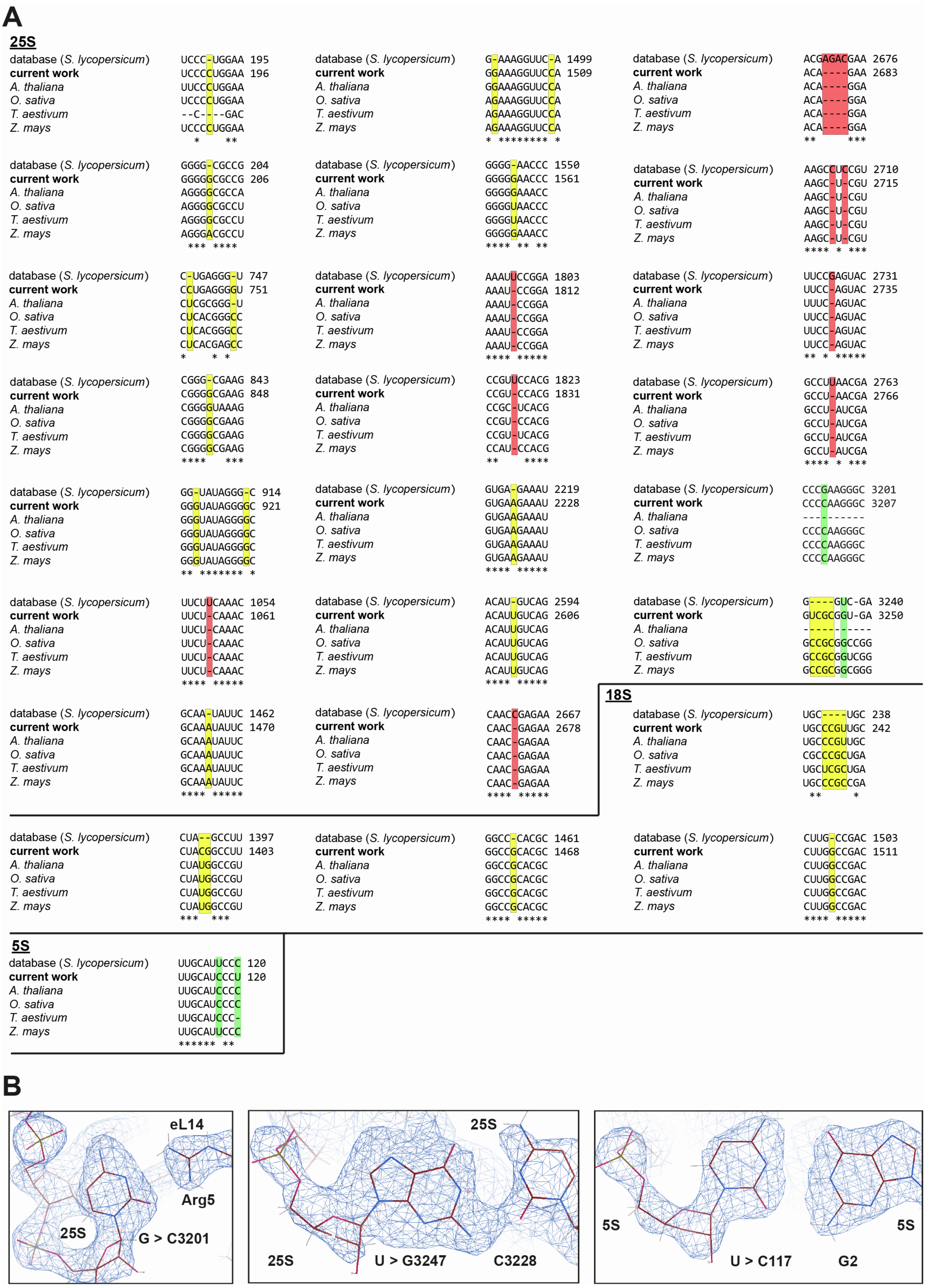
A
25S
database (S. lycopersicum) UCCC-UGGAA 195
current work UCCCCUGGAA 196
A. thaliana UUCCCUGGAA
O. sativa UCCCCUGGAA
T. aestivum --C----GAC
Z. mays UCCCCUGGAA
database (S. lycopersicum) G-AAAGGUUC-A 1499
current work GGAAAGGUUCCA 1509
A. thaliana GGAAAGGUUCCA
O. sativa AGAAAGGUUCCA
T. aestivum AGAAAGGUUCCA
Z. mays AGAAAGGUUCCA
database (S. lycopersicum) ACGAGACGAA 2676
current work ACA----GAA 2683
A. thaliana ACA----GGA
O. sativa ACA----GGA
T. aestivum ACA----GGA
Z. mays ACA----GGA
database (S. lycopersicum) GGGG-CGCCG 204
current work GGGGGCGCCG 206
A. thaliana AGGGGCGCCA
O. sativa AGGGGCGCCU
T. aestivum AGGGGCGCCU
Z. mays AGGGACGCCU
database (S. lycopersicum) GGGG-AACCC 1550
current work GGGGGAACCC 1561
A. thaliana GGGGGAAACC
O. sativa GGGGUAACCC
T. aestivum GGGGUAACCC
Z. mays GGGGGAAACC
database (S. lycopersicum) AAGCCUCCGU 2710
current work AAGC-U-CGU 2715
A. thaliana AAGC-U-CGU
O. sativa AAGC-U-CGU
T. aestivum AAGC-U-CGU
Z. mays AAGC-U-CGU
database (S. lycopersicum) C-UGAGGG-U 747
current work CCUGAGGGGU 751
A. thaliana CUCGCGGG-U
O. sativa CUCACGGGCC
T. aestivum CUCACGGGCC
Z. mays CUCACGAGCC
database (S. lycopersicum) AAAUUCCGGA 1803
current work AAAU-CCGGA 1812
A. thaliana AAAU-CCGGA
O. sativa AAAU-CCGGA
T. aestivum AAAU-CCGGA
Z. mays AAAU-CCGGA
database (S. lycopersicum) UUCCGAGUAC 2731
current work UUCC-AGUAC 2735
A. thaliana UUUC-AGUAC
O. sativa UUCC-AGUAC
T. aestivum UUCC-AGUAC
Z. mays UUCC-AGUAC
database (S. lycopersicum) CGGG-CGAAG 843
current work CGGGGCGAAG 848
A. thaliana CGGGGUAAAG
O. sativa CGGGGCGAAG
T. aestivum CGGGGCGAAG
Z. mays CGGGGCGAAG
database (S. lycopersicum) CCGUUCCACG 1823
current work CCGU-CCACG 1831
A. thaliana CCGC-UCACG
O. sativa CCGU-CCACG
T. aestivum CCGU-UCACG
Z. mays CCAU-CCACG
database (S. lycopersicum) GCCUUAACGA 2763
current work GCCU-AACGA 2766
A. thaliana GCCU-AUCGA
O. sativa GCCU-AUCGA
T. aestivum GCCU-AUCGA
Z. mays GCCU-AUCGA
database (S. lycopersicum) GG-UAUAGGG-C 914
current work GGGUAUAGGGGC 921
A. thaliana GGGUAUAGGGGC
O. sativa GGGUAUAGGGGC
T. aestivum GGGUAUAGGGGC
Z. mays GGGUAUAGGGGC
database (S. lycopersicum) GUGA-GAAAU 2219
current work GUGAAGAAAU 2228
A. thaliana GUGAAGAAAU
O. sativa GUGAAGAAAU
T. aestivum GUGAAGAAAU
Z. mays GUGAAGAAAU
database (S. lycopersicum) CCCGAAGGGC 3201
current work CCCCAAGGGC 3207
A. thaliana ----------
O. sativa CCCCAAGGGC
T. aestivum CCCCAAGGGC
Z. mays CCCCAAGGGC
database (S. lycopersicum) UUCUUCAAAC 1054
current work UUCU-CAAAC 1061
A. thaliana UUCU-CAAAC
O. sativa UUCU-CAAAC
T. aestivum UUCU-CAAAC
Z. mays UUCU-CAAAC
database (S. lycopersicum) ACAU-GUCAG 2594
current work ACAUUGUCAG 2606
A. thaliana ACAUUGUCAG
O. sativa ACAUUGUCAG
T. aestivum ACAUUGUCAG
Z. mays ACAUUGUCAG
database (S. lycopersicum) G----GUC-GA 3240
current work GUCGCGGU-GA 3250
A. thaliana -----------
O. sativa GCCGCGGCCGG
T. aestivum GCCGCGGUCGG
Z. mays GCCGCGGCGGG
database (S. lycopersicum) GCAA-UAUUC 1462
current work GCAAAUAUUC 1470
A. thaliana GCAAAUAUUC
O. sativa GCAAAUAUUC
T. aestivum GCAAAUAUUC
Z. mays GCAAAUAUUC
database (S. lycopersicum) CAACGAGAA 2667
current work CAAC-GAGAA 2678
A. thaliana CAAC-GAGAA
O. sativa CAAC-GAGAA
T. aestivum CAAC-GAGAA
Z. mays CAAC-GAGAA
18S
database (S. lycopersicum) UGC----UGC 238
current work UGCCCGUUGC 242
A. thaliana UGCCCGUUGC
O. sativa CGCCCGCUGA
T. aestivum UGCUCGCUGA
Z. mays UGCCCGCCGA
database (S. lycopersicum) CUA--GCCUU 1397
current work CUACGGCCUU 1403
A. thaliana CUAUGGCCGU
O. sativa CUAUGGCCGU
T. aestivum CUAUGGCCGU
Z. mays CUAUGGCCGU
database (S. lycopersicum) GGCC-CACGC 1461
current work GGCCGCACGC 1468
A. thaliana GGCCGCACGC
O. sativa GGCCGCACGC
T. aestivum GGCCGCACGC
Z. mays GGCCGCACGC
database (S. lycopersicum) CUUG-CCGAC 1503
current work CUUGGCCGAC 1511
A. thaliana CUUGGCCGAC
O. sativa CUUGGCCGAC
T. aestivum CUUGGCCGAC
Z. mays CUUGGCCGAC
5S
database (S. lycopersicum) UUGCAUUCCC 120
current work UUGCAUCCCU 120
A. thaliana UUGCAUCCCC
O. sativa UUGCAUCCCC
T. aestivum UUGCAUCCC-
Z. mays UUGCAUUCCC
B
eL14
Arg5
25S
G > C3201
25S
25S
U > G3247
C3228
5S
5S
U > C117
G2

ORGANISM	SEQUENCE			
	**25S**	**18S**	**5.8S**	**5S**
Database (*S. lycopersicum*)	X13557.1	X51576.1	MH573911.1	X06842.1
Arabidopsis thaliana	LR797810.1 (3150-6000)	LR797808.1 (8000-10000)	LR782543.1 (5680-5860)	LR782545.1 (3150170-3150310)
Oryza sativa	AP014965.1 (29700-33200)	LC086814.1 (1-3000)	XR_003238527.1 (890-1070)	AP014967.1 (12099160-12099300)
Triticum aestivum	3J62_a	3J5Z_d	M10469.1	KY439627.1 (298-417)
Zea mays	NR_028022.2	AF168884.1	MF780726.1 (150-320)	DQ351339.1 (186-315)

rRNA sequences used for the multiple sequence alignments:

Supplementary Figure S5: rRNA sequence discrepancies with the available database. (a) Multiple sequence alignment, showing reannotations (green), deletions (red), insertions (yellow). The density and modifications have been assessed in accordance with previous studies from the lab at a similar resolution level (Itoh et al., 2022a; Itoh et al., 2022b). (b) Examples of point mutations identified from the density in 60S.

>25S
GCGACCCCAGGUCAGGCGGGAUUACCCGCUGAGUUUAAGCAUAUCAAUAAGC
GGAGGAAAAGAAACUUACAAGGAUUCCCCUAGUAACGGCGAGCGAACCGGGA
ACAGCCCAGCCUUAGAAUCGGGCGGCUCCGUCGUCCGAAUUGUAGUCUGGAG
AAGCGUCCUCAGCGGCGGACCGGGCCCAAGUCCCCUGGAAGGGGGCGCCGGA
GAGGGUGAGAGCCCCGUCGUGCCCGGACCCUGUCGCACCACGAGGCGCUGUC
UACGAGUCGGGUUGUUUGGGAAUGCAGCCCAAAUCGGGCGGUGAAUUCCGUC
CAAGGCUAAAUACUGGCGAGAGACCGAUAGCGAACAAGUACCGCGAGGGAAA
GAUGAAAAGGACUUUGAAAAGAGAGUCAAAGAGUGCUUGAAAUUGUCGGGAG
GGAAGCGGAUGGGGGCCGGCGAUGCGCCCCGGUCGGAUGUGGAACGGCGACG
AGCCGGUCCGCCGAUCGACUCGGGGCGUGGACCAGCGUGGAUUGGGGGGCG
GCCAAAGCCCGGGCUCUCGAUACGCCCGUGGAACGCCGUCUCCCCGAUUGUG
GAAGGCAGCGCGCGCCUCCGGCGUGCUUCGGCAUCUGCGCGCUCCGGACGCU
GGCCUGUGGGCUCCCCAUUCGACCCGUCUUGAAACACGGACCAAGGAGUCUG
ACAUGUGUGCGAGUCAACGGGCGAGUAAACCCGUAAGGCGUAAGGAAGCUGA
UUGGUGGGAUCCCCCUGAGGGGUGCACCGCCGACCGACCUUGAUCUUCUGAG
AAGGGUUCGAGUGUGAGCAUACCUGUCGGGACCCGAAAGAUGGUGAACUAUG
CCUGAGCGGGGCGAAGCCAGAGGAAACUCUGGUGGAGGCCCGCAGCGAUACU
GACGUGCAAAUCGUUCGUCUGACUUGGGUAUAGGGGCGAAAGACUAAUCGAA
CCGUCUAGUAGCUGGUUCCCUCCGAAGUUUCCCUCAGGAUAGCUGGAGCUCG
CGUGCGAGUUCUAUCGGGUAAAGCCAAUGAUUAGAGGCCUCGGGGGCGCAAC
GCCCUCGACCUAUUCUCAAACUUUAAAUAGGUAGGACGGCGCGGCUGCUUUG
UUGAGCCGCGCCACGGAAUCAAGAGCUCCAAGUGGGCCAUUUUUGGUAAGCA
GAACUGGCGAUGCGGGAUGAACCGGAAGCCGGGUUACGGUGCCAAACUGCGC
GCUAACCUAGAUCCCACAAAGGGUGUUGGUCGAUUAAGACAGCAGGACGGUG
GUCAUGGAAGUCGAAAUCCGCUAAGGAGUGUGUAACAACUCACCUGCCGAAU
CAACUAGCCCCGAAAAUGGAUGGCGCUUAAGCGCGCGACCUACACCCGGCCG
UCGGGGCAAGUGCCAGGCCCCGAUGAGUAGGAGGGCGCGGCGGUCGCUGCAA
AACCUUGGGCGCGAGCCUGGGCGGAGCGGCCGUCGGUGCAGAUCUUGGUGGU
AGUAGCAAAUAUUCAAAUGAGAACUUUGAAGGCCGAAGAGGGGAAAGGUUCC
AUGUGAACGGCACUUGCACAUGGGUUAGUCGAUCCUAAGGGUCGGGGGAACC
CCGACAGAUAGCGCGUUUCGCGCGUACUCCGAAAGGGAAUCGGGUUAAAAUU
CCUGAACCGGGACGUGGCGGUUGACGGCAACGUUAGGAAGUCCGGAGACGUC
GGCGGGAGCCUCGGGAAGAGUUAUCUUUUCUGUUUAACAGCCUGCCCACCCU
GGAAUCGGCUCAGCCGGAGGUAGGGUCCAGCGGCUGGAAGAGCACCGCACGU
CGCGUGGUGUCCGGUGCGCUCCCGGCGGCCCUUGAAAAUCCGGAGGACCGAA
UGCCGUCCACGCCCGGUCGUACUCAUAACCGCAUCAGGUCUCCAAGGUGAAC
AGCCUCUGGUCGAUGGAACAAUGUAGGCAAGGGAAGUCGGCAAAAUGGAUCC
GUAACUUCGGGAAAAGGAUUGGCUCUGAGGGCUGGGCACGGGGGUCCCAGUC
CCGAACCCGUCGGCUGUCGGUGGACUGCUCGAGCUGCUCCCGCGGCGAGAGC
GGGUCGCCGCGUGCCGGCCGGGGGACGGACUGGGAACGGUUCCUUCGGGGGC
CUUCCCCGGGCGUCGAACAGCCAACUCAGAACUGGUACGGACAAGGGGAAUC
CGACUGUUUAAUUAAAACAAAGCAUUGCGAUGGUCCCAACGGAUGUUUACGC
AAUGUGAUUUCUGCCCAGUGCUCUGAAUGUCAAAGUGAAGAAAUUCAACCAA
GCGCGGGUAAACGGCGGGAGUAACUAUGACUCUCUUAAGGUAGCCAAAUGCC
UCGUCAUCUAAUUAGUGACGCGCAUGAAUGGAUUAACGAGAUUCCCACUGUC
CCUGUCUACUAUCCAGCGAAACCACAGCCAAGGGAACGGGCUUGGCAGAAUC
AGCGGGGAAAGAAGACCCUGUUGAGCUUGACUCUAGUCCGACUUUGUGAAAU
GACUUGAGAGGUGUAGUAUAAGUGGGAGCCGAAAGGCGAAAGUGAAAUACCA
CUACUUUUAACGUUAUUUUACUUAUUCCGUGAAUCGGAAGCGGGGCACUGCC
CCUCUUUUUGGACCCAAGGCUCGCUUCGCGGGCCGAUCCGGGCGGAAGACAU
UGUCAGGUGGGGAGUUUGGCUGGGGCGGCACAUCUGUUAAAAGAUAACGCAG
GUGUCCUAAGAUGAGCUCAACGAGAACAGAAAUCUCGUGUGGAACAGAAGGG
UAAAAGCUCGUUUGAUUCUGAUUUCCAGUACGAAUACGAACCGUGAAAGCGU
GGCCUAACGAUCCUUUAGACCUUCGGAAUUCGAAGCUAGAGGUGUCAGAAAA
GUUACCACAGGGAUAACUGGCUUGUGGCAGCCAAGCGUUCAUAGCGACGUUG
CUUUUUGAUCCUUCGAUGUCGGCUCUUCCUAUCAUUGUGAAGCAGAAUUCAC
CAAGUGUUGGAUUGUUCACCCACCAAUAGGGAACGUGAGCUGGGUUUAGACC
GUCGUGAGACAGGUUAGUUUUACCCUACUGAUGACAGUGUCGCAAUAGUAAU
UCAACCUAGUACGAGAGGAACCGUUGAUUCACACAAUUGGCCAUCGCGCUUG
GUUGAAAAGCCAGUGGCGCGAAGCUACCGUGUGCUGGAUUAUGACUGAACGC
CUCUAAGUCAGAAUCCGGGCUAGAAGCGACGCAUGCGCCCGCCGUCCGCUUG
CCGACCCGCAGUAGGGGCCUUUGGCCCCCAAGGGCACGUGUCGUUGGCUAAG
UCGCCGCGACGGAAGCGUCGCGGUGACCGCCUUGAAGUACAAUUUCCAUCGA
GCGGCGGGUAGAAUCCUUUGCAGACGACUUAAAUACGCGACGGGGUAUUGUA
AGUGGCAGAGUGGCCUUGCUGCCACGAUCCACUGAGAUUCAGCCCUUUGUCG
CUCCGAUUCGU
>18
UACCUGGUUGAUCCUGCCAGUAGUCAUAUGCUUGUCUCAAAGAUUAAGCCAU
GCAUGUGUAAGUAUGAACAAAUUCAGACUGUGAAACUGCGAAUGGCUCAUUA
AAUCAGUUAUAGUUUGUUUGAUGGUAUCUACUACUCGGAUAACCGUAGUAAU
UCUAGAGCUAAUACGUGCAACAAACCCCGACUUCUGGAAGGGAUGCAUUUAU
UAGAUAAAAGGUCGACGCGGGCUCUGCCCGUUGCUGCGAUGAUUCAUGAUAA
CUCGACGGAUCGCACGGCCAUCGUGCCGGCGACGCAUCAUUCAAAUUUCUGC
CCUAUCAACUUUCGAUGGUAGGAUAGUGGCCUACCAUGGUGGUGACGGGUGA
CGGAGAAUUAGGGUUCGAUUCCGGAGAGGGAGCCUGAGAAACGGCUACCACA
UCCAAGGAAGGCAGCAGGCGCGCAAAUUACCCAAUCCUGACACGGGGAGGUA
GUGACAAUAAAUAACAAUACCGGGCUCUAUGAGUCUGGUAAUUGGAAUGAGU
ACAAUCUAAAUCCCUUAACGAGGAUCCAUUGGAGGGCAAGUCUGGUGCCAGC
AGCCGCGGUAAUUCCAGCUCCAAUAGCGUAUAUUUAAGUUGUUGCAGUUAAA
AAGCUCGUAGUUGGACUUUGGGAUGGGCCGGCCGGUCCGCCCUAGGUGUGCA
CCGGUCGUCUCGUCCCUUCUGUCGGCGAUGCGCUCCUGGCCUUAAUUGGCCG
GGUCGUGCCUCCGGCGCUGUUACUUUGAAGAAAUUAGAGUGCUCAAAGCAAG
CCUACGCUCUGUAUACAUUAGCAUGGGAUAACAUUAUAGGAUUUCGGUCCUA
UUACGUUGGCCUUCGGGAUCGGAGUAAUGAUUAACAGGGACAGUCGGGGGCA
UUCGUAUUUCAUAGUCAGAGGUGAAAUUCUUGGAUUUAUGAAAGACGAACAA
CUGCGAAAGCAUUUGCCAAGGAUGUUUUCAUUAAUCAAGAACGAAAGUUGGG
GGCUCGAAGACGAUCAGAUACCGUCCUAGUCUCAACCAUAAACGAUGCCGAC
CAGGGAUCGGCGGAUGUUGCUUUUAGGACUCCGCCGGCACCUUAUGAGAAAU
CAAAGUUUUUGGGUUCCGGGGGGAGUAUGGUCGCAAGGCUGAAACUUAAAGG
AAUUGACGGAAGGGCACCACCAGGAGUGGAGCCUGCGGCUUAAUUUGACUCA
ACACGGGGAAACUUACCAGGUCCAGACAUAGUAAGGAUUGACAGACUGAGAG
CUCUUUCUUGAUUCUAUGGGUGGUGGUGCAUGGCCGUUCUUAGUUGGUGGAG
CGAUUUGUCUGGUUAAUUCCGUUAACGAACGAGACCUCAGCCUGCUAACUAG
CUAUGCGGAGGUAUCCCUUCGCGGCCAGCUUCUUAGAGGGACUACGGCCUUU
UAGGCCGCGGAAGUUUGAGGCAAUAACAGGUCUGUGAUGCCCUUAGAUGUUC
UGGGCCGCACGCGCGCUACACUGAUGUAUUCAACGAGCUUAUAGCCUUGGCC
GACAGGCCCGGGUAAUCUUUGAAAUUUCAUCGUGAUGGGGAUAGAUCAUUGC
AAUUGUUGGUCUUCAACGAGGAAUUCCUAGUAAGCGCGAGUCAUCAGCUCGC
GUUGACUACGUCCCUGCCCUUUGUACACACCGCCCGUCGCUCCUACCGAUUG
AAUGAUCCGGUGAAAUGUUCGGAUCGCGGCGACGUGGGCGGUUCGCUGCCCG
CGACGUCGCGAGAAGUCCAUUGAACCUUAUCAUUUAGAGGAAGGAGAAGUCG
UAACAAGGUUUCCGUAGGUGAACCUGCGGAAGGAUCAUUG
>5.8S
ACAAACGACUCUCGGCAACGGAUAUCUCGGCUCUCGCAUCGAUGAAGAACGU
AGCGAAAUGCGAUACUUGGUGUGAAUUGCAGAAUCCCGUGAACCAUCGAGUC
UUUGAACGCAAGUUGCGCCCGAAGCCAUUUGGCCGAGGGCACGUCUGCCUGG
GCGUCACGC
>5S
GGAUGCGAUCAUACCAGCACUAACGCACCGGAUCCCAUCAGAACUCCGAA
GUUAAGCGUGCUUGGGCGAGAGUAGUACUAGGAUGGGUGACCCCCUGGGA
AGUCCUCGUGUUGCAUCCCU

Supplementary Figure S6: Corrected *S. lycopersicum* rRNA sequences.

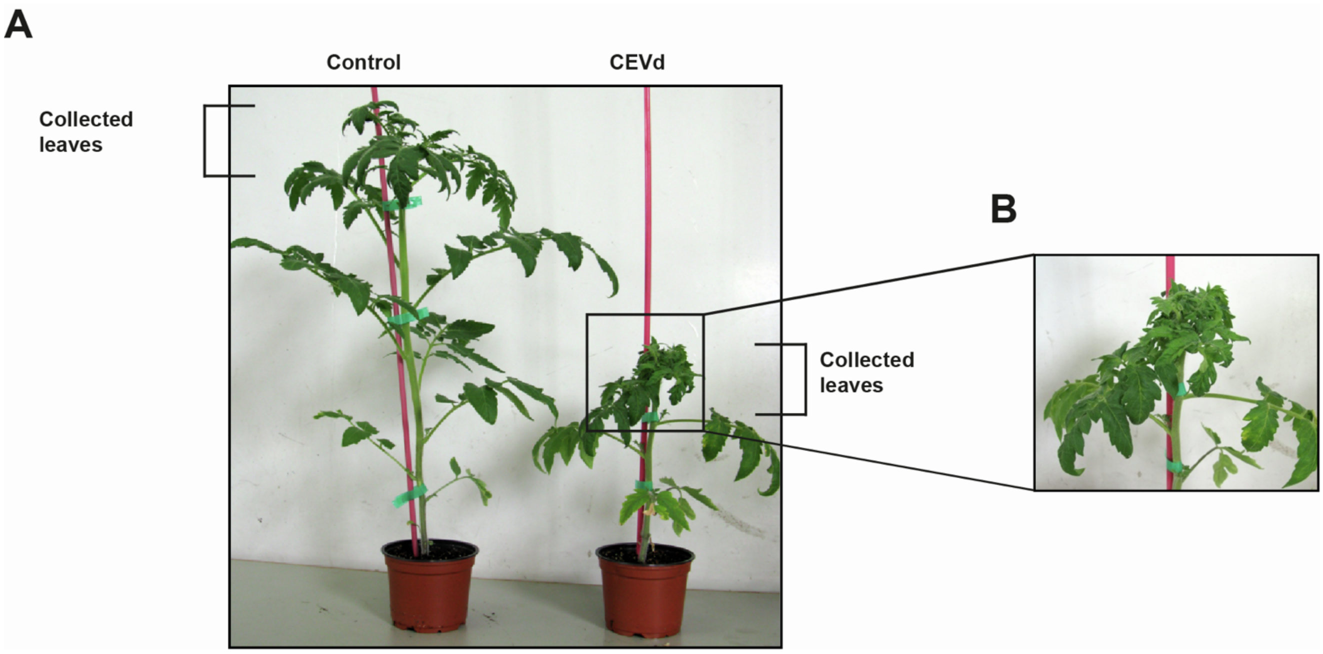

Supplementary Figure S7: Control and stressed plants used for the experiments three weeks post-inoculation (a) To stimulate stress conditions, plants were CEVd (Citrus exocortis viroid) infected by puncturing. (b) Zoom in to the plant's apex that was used to collect leaves for the experiment.

Supplementary Table S1: Cryo-EM data collection, processing and model refinement statistics.

	60S	40S body	40S head	Combined
**Data collection and processing**				
Microscope	Titan Krios	Titan Krios	Titan Krios	Titan Krios
Detector	K2 Summit	K2 Summit	K2 Summit	K2 Summit
Magnification	165,000	165,000	165,000	165,000
Voltage [kV]	300	300	300	300
Total electron exposure [e^{-}/Å^2]	29–32	29–32	29–32	29–32
Defocus range [μm]	−1.0 to −2.8	−1.0 to −2.8	−1.0 to −2.8	−1.0 to −2.8
Pixel size [Å]	0.82–0.83	0.82–0.83	0.82–0.83	0.82–0.83
Symmetry imposed	C_1	C_1	C_1	C_1
Final particle	335,806	335,806	335,806	335,806
Resolution [Å]	2.35	2.53	2.58	2.38
Map-sharpening *B* factor [Å^2]	−44.17	−44.17	−43.47	−47.28
**Refinement**				
Model composition				
Total atoms (non-hydrogen / hydrogen)	127,702 / 89,168	47,741 / 36,377	24,563 / 19587	199,939 / 145,068
Chains (RNA/ protein)	4 / 41	1 / 21	3 / 13	7 / 73
RNA residues (non-modified / modified)	3,252 / 127	1060 / 50	464 / 27	4,776 / 204
Protein residues (non-modified / modified)	6,329 / 2	2,911 / 1	1,730 / 0	10,963 / 3
Metal ions (Mg^{2+}/ K^{+}/ Zn^{2+})	276 / 90 / 4	60 / 19 / 1	28 / 10 / 1	364 / 119 / 6
Ligands (SPD / SPM / PUT/ sugar)	1 / 3 / 0 / 0	0 / 0 / 0 / 0	0 / 0 / 1 / 1	1 / 3 / 1 / 1
Waters	3,939	570	225	4,734
Model to map CC (CC_{mask} / CC_{box} / CC_{peaks} / CC_{volume})	0.83 / 0.74 / 0.69 / 0.80	0.86 / 0.74 / 0.69 / 0.82	0.89 / 0.79 / 0.75 / 0.85	0.83 / 0.78 / 0.75 / 0.80
Resolution [Å] by model-to-map FSC, threshold 0.50 (masked/ unmasked)	2.23 / 2.24	2.38 / 2.38	2.34 / 2.43	2.31 / 2.31
Average *B* factor [Å^2] (RNA / protein / metal ion and ligand / water)	19 / 13 / 8.2 / 4.9	23 / 25 / 13 / 11	25 / 35 / 20 / 15	21 / 18 / 12 / 8.1
R.m.s. deviations				
Bond lengths [Å]	0.002	0.002	0.003	0.002
Bond angles [°]	0.412	0.441	0.489	0.387
**Validation**				
Clash score	1.86	1.94	1.89	2.02
Rotamer outliers [%]	0.07	0.08	0.00	0.06
Ramachandran plot [%] (Favored / allowed/ disallowed)	98.70 / 1.29 / 0.02	99.13 / 0.87 / 0.00	98.47 / 1.53 / 0.00	99.04 / 0.95 / 0.01
CaBLAM outliers [%]	1.06	0.82	1.20	0.96
MolProbity score	0.95	0.96	0.95	0.97
**Accession ID**				
EMDB ID	EMD-14001	EMD-14002	EMD-14003	EMD-14004
PDB ID	7QIW	7QIX	7QIY	7QIZ

Supplementary Table S2: Re-annotations of the rRNAs in 60S, composed of deletions, insertions and point mutations observed according to the map.

rRNA	position	residue/s	re-annotation
**25S**			
H38a	1056-1057	U	deletion
H56	1807-1808	U	deletion
H54	1826-1827	U	deletion
H84	2673-2674	C	deletion
H84	2680-2681	AGAC	deletion
H83	2711-2712	C	deletion
H83	2712-2713	C	deletion
H86	2730-2731	G	deletion
H82	2761-2762	U	deletion
H19	191	C	insertion
H19	201	G	insertion
ES9	743	C	insertion
ES9	750	G	insertion
ES7a	843	G	insertion
H33	912	G	insertion
H33	920	G	insertion
H47	1465	A	insertion
H49	1499	G	insertion
H50	1508	C	insertion
H52	1556	G	insertion
H66	2223	A	insertion
H75	2601	U	insertion
ES39b	3242-3245	UCGU	insertion
ES39a	3201	G → C	point mutation
ES39a	3247	U → G	point mutation
**5S**			
	117	U → C	point mutation
	120	C → U	point mutation

Supplementary Table S3: Re-annotations of the rRNA in 40S, composed of insertions and point mutations observed according to the map.

rRNA	position	residue/s	re-annotation
**18S**			
es3b	236-239	CCGU	insertion
h40	1397-1398	CG	insertion
h30	1463	G	insertion
es10	1506	G	insertion

Supplementary Table S4: Plant specific rRNA modifications in 60S.

rRNA	residue	modification
**25S**		
H11	U35	Pseudouridine
H11	U48	2′-*O*-methylation
H12	U68	Pseudouridine
H15	U144	2′-*O*-methylation
H3	A369	2′-*O*-methylation
H24	U378	Pseudouridine
H3	G399	2′-*O*-methylation
H27	U675	2′-*O*-methylation
H27	U804	2′-*O*-methylation
H32	A946	2′-*O*-methylation
H38a	U1068	2′-*O*-methylation
H39	U1134	Pseudouridine
H42	U1230	Pseudouridine
H43	U1250	Pseudouridine
H43	A1252	2′-*O*-methylation
H43	A1264	2′-*O*-methylation
H44	U1279	2′-*O*-methylation
H44	U1288	Pseudouridine
H45	A1378	2′-*O*-methylation
H47	U1474	Pseudouridine
H47	C1480	2′-*O*-methylation
H47	U1482	Pseudouridine
H51	U1537	2′-*O*-methylation
H49a	C1849	2′-*O*-methylation
H49a	C1852	2′-*O*-methylation
H49a	G1857	2′-*O*-methylation
H63	U2116	2′-*O*-methylation
H64	A2129	2′-*O*-methylation
H68	U2228	Pseudouridine
H68	G2239	2′-*O*-methylation
H71	C2296	2′-*O*-methylation
H75	U2435	Pseudouridine
H76	U2449	Pseudouridine
H76	U2458	2′-*O*-methylation
H78	G2486	2′-*O*-methylation
H76	U2504	Pseudouridine
H79	U2521	Pseudouridine
H84	C2686	2′-*O*-methylation
H83	U2716	Pseudouridine
H86	U2721	2′-*O*-methylation
H86	U2739	2′-*O*-methylation
H89	C2840	2′-*O*-methylation
**25S**		
H90	U2887	2′-*O*-methylation
H91	A2915	2′-*O*-methylation
H73	U2996	Pseudouridine
H100	U3305	2′-*O*-methylation
**5.8S**		
H5	A48	2′-*O*-methylation
H7	U79	Pseudouridine

Supplementary Table S5: Plant specific rRNA modifications in 40S.

rRNA	residue	modification
**18S**		
h7	U103	Pseudouridine
h8	C140	2′-*O*-methylation
es3a	U188	Pseudouridine
es3b	G246	2′-*O*-methylation
h9	U255	Pseudouridine
h9	U258	Pseudouridine
h12	U383	Pseudouridine
h18	U584	Pseudouridine
h3	U603	2′-*O*-methylpseudouridine
h19	U614	2′-*O*-methylation
h21	U753	Pseudouridine
h22	U949	Pseudouridine
h24	U1012	2′-*O*-methylation
h25	U1106	Pseudouridine
h32	U1210	Pseudouridine
h33	U1263	2′-*O*-methylation
h33	U1265	2′-*O*-methylation
h34	U1447	2′-*O*-methylation
h41	U1485	Pseudouridine
h41	U1535	Pseudouridine
h41	U1538	Pseudouridine
h42	A1579	2′-*O*-methylation
h44	A1758	2′-*O*-methylation

Supplementary Table S6: Conserved rRNA modifications in 60S.

rRNA	residue	modification	rRNA	residue	modification
**25S**			**25S**		
H11	U44	2′-*O*-methylation	H64	A2324	2′-*O*-methylation
H25a	A656	N1-methylation	H64	A2329	2′-*O*-methylation
H25a	A660	2′-*O*-methylation	H61	C2340	2′-*O*-methylation
H27	C674	2′-*O*-methylation	H61	U2350	2′-*O*-methylation
H32	G815	2′-*O*-methylation	H61	U2352	Pseudouridine
H32	A817	2′-*O*-methylation	H72	C2368	2′-*O*-methylation
H33	A827	2′-*O*-methylation	H73	G2394	2′-*O*-methylation
H33	U829	Pseudouridine	H73	G2398	2′-*O*-methylation
H35	A886	2′-*O*-methylation	H74	G2412	2′-*O*-methylation
H35	U895	Pseudouridine	H74	U2413	2′-*O*-methylation
H35a	G918	2′-*O*-methylation	H74	U2419	Pseudouridine
H37	U970	Pseudouridine	H75	U2424	2′-*O*-methylation
H38a	U1002	Pseudouridine	H77	C2497	2′-*O*-methylation
H38a	U1016	Pseudouridine	H80	U2617	Pseudouridine
H38a	U1054	Pseudouridine	H80	G2623	2′-*O*-methylation
H38a	U1064	Pseudouridine	H81	A2644	2′-*O*-methylation
H39	U1133	Pseudouridine	H81	U2654	2′-*O*-methylation
H39	U1135	Pseudouridine	H81	G2655	2′-*O*-methylation
H39	A1144	2′-*O*-methylation	H86	U2733	2′-*O*-methylation
H26	C1448	2′-*O*-methylation	H87	U2748	Pseudouridine
H26a	A1460	2′-*O*-methylation	H88	G2795	2′-*O*-methylation
H26a	G1461	2′-*O*-methylation	H88	G2797	2′-*O*-methylation
H49	C1862	2′-*O*-methylation	H74	G2819	2′-*O*-methylation
H61	U1894	2′-*O*-methylation	H89	U2830	Pseudouridine
H61	U1909	Pseudouridine	H89	U2847	Pseudouridine
H64	G2126	2′-*O*-methylation	H89	U2858	Pseudouridine
H64	G2127	2′-*O*-methylation	H89	U2869	Pseudouridine
H65	U2137	Pseudouridine	H90	C2874	C^5-methylation
H65	U2139	Pseudouridine	H90	C2883	2′-*O*-methylation
H67	U2194	Pseudouridine	H90	U2884	Pseudouridine
H68	C2200	2′-*O*-methylation	H91	U2898	Pseudouridine
H68	U2214	Pseudouridine	H92	G2921	2′-*O*-methylation
H68	A2223	2′-*O*-methylation	H92	U2925	2′-*O*-methylation
H69	U2257	Pseudouridine	H92	G2926	2′-*O*-methylation
H69	A2259	2′-*O*-methylation	H92	U2927	Pseudouridine
H69	U2261	Pseudouridine	H90	U2948	Pseudouridine
H69	U2263	Pseudouridine	H90	A2950	2′-*O*-methylation
H69	U2267	Pseudouridine	H90	C2952	2′-*O*-methylation
H69	U2269	Pseudouridine	H90	U2957	N^3-methylation
H70	C2281	C^5-methylation	H93	U2959	Pseudouridine
H70	A2284	2′-*O*-methylation	H93	C2963	2′-*O*-methylation
H71	G2291	2′-*O*-methylation	H93	U2979	Pseudouridine
H67	U2317	Pseudouridine	H97	U3114	Pseudouridine
H67	U2321	Pseudouridine			
**5.8S**					
H3	U23	Pseudouridine			
H7	G80	2′-*O*-methylation			

Supplementary Table S7: Conserved rRNA modifications in 40S.

rRNA	residue	modification	rRNA	residue	modification
**18S**			**18S**		
h4	A28	2′-*O*-methylation	h39	U1383	2′-*O*-methylation
h5	C38	2′-*O*-methylation	h34	G1433	2′-*O*-methylation
h7	U111	Pseudouridine	H42	U1567	Pseudouridine
h7	U121	Pseudouridine	h42	G1581	N^7-methylation
h7	U123	2′-*O*-methylation	h28	U1634	Pseudouridine
h8	A162	2′-*O*-methylation	h44	C1645	2′-*O*-methylation
h9	U208	Pseudouridine	h44	A1771	N^6-methylation
h7	U300	Pseudouridine	h45	C1781	N^4-acetylation
h7	U306	Pseudouridine	h45	A1789	N^6,N^6-dimethylation
h11	U362	Pseudouridine	h45	A1790	N^6,N^6-dimethylation
h13	G392	2′-*O*-methylation			
h14	C418	2′-*O*-methylation			
h15	A440	2′-*O*-methylation			
h15	U451	Pseudouridine			
h15	A468	2′-*O*-methylation			
h5	C473	2′-*O*-methylation			
h17	A544	2′-*O*-methylation			
h18	U581	2′-*O*-methylation			
h4	G598	2′-*O*-methylation			
h3	U605	Pseudouridine			
h19	A622	2′-*O*-methylation			
h20	U635	Pseudouridine			
h21	U762	Pseudouridine			
h21	A800	2′-*O*-methylation			
h21	U809	Pseudouridine			
h23	U912	Pseudouridine			
h22	U950	Pseudouridine			
h20	A977	2′-*O*-methylation			
h24	U1002	Pseudouridine			
h24	U1027	Pseudouridine			
h27	U1120	Pseudouridine			
h30	U1178	Pseudouridine			
h30	U1184	Pseudouridine			
h31	U1190	Pseudouridine			
h31	U1194	1-methyl-3-(3-amino-3-carboxypropyl)pseudouridine			
h32	U1217	Pseudouridine			
h32	C1218	2′-*O*-methylation			
h33	U1234	2′-*O*-methylation			
h34	U1272	2′-*O*-methylation			
h34	G1274	2′-*O*-methylation			
h34	C1283	N^4-acetylation			
h35	U1293	Pseudouridine			
h36	U1304	Pseudouridine			
h37	U1308	Pseudouridine			
h37	U1313	Pseudouridine			
h37	A1329	2′-*O*-methylation			

Supplementary Table S9: Oligonucleotides.

Oligonucleotide	Sequence[a]
Tmt18S-270-chimera	AmCmGmAmUmGmGmCmCmGm(TGCG)AmUmCmCmGmUmCmGm
c18S-385-chimera	GmGmCmUmCmCmCmUmCm(TCCG)GmAmAmUmCmGmAmAmCmCmCm
c18S-580-chimera	AmGmCmUmGmGmAmAm(TTAC)CmGmCmGmGmCmUmGmCmUmGmGm
Tmt18S-831-chimera	CmCmGmAmAmGmGmCmCmAmAmCmGm(TAAT)AmGmGmAmCmCmGmAm
h18S-1210-chimera	CmCmUmGmGmUmGmGmUmGmCmCm(CTTC)CmGmUmCmAmAmUm
Tmt18S-1332-chimera	AmGmCmAmGmGmCmUmGmAm(GGTC)UmCmGmUmUmCmGmUmUmAm
Tmt18S-1597-chimera	AmGmCmUmGmAmUmGmAmCmUmCm(GCGC)UmUmAmCmUmAmGmGmAm
Tmt18S-1679-chimera	CmGmCmCmGmCmGmAmUmCmCmGm(AACA)UmUmUmCmAmCmCmGm
Tmt25S-418-chimera	CmCmCmCmAmUmCmCmGmCmUmUm(CCCT)CmCmCmGmAmCmAmAm
Tmt25S-643-chimera	UmCmAmAmGmAmCmGmGmGmUmCmGm(AATG)GmGmGmAmGmCmCm
Tmt25S-850-chimera	AmGmAmGmUmUmUmCmCmUmCmUm(GGCT)UmCmGmCmCmCmCmGm
h28S-1866-chimera	CmGmCmCmAmGmUmUmCmUmGmCmUmUm(ACCA)AmAmAmGmUmGmGmCmCm
Tmt25S-1521-chimera	AmCmCmCmAmUmGmUmGmCmAm(AGTG)CmCmGmUmUmCmAmCmAmUm
Tmt25S-1859-chimera	UmCmAmCmCmUmUmGmGmAmGm(ACCT)GmAmUmGmCmGmGmUmUm
Tmt25S-2119-chimera	CmCmCmCmUmUmGmUmCm(CGTA)CmCmAmGmUmUmCmUmGmAmGmUm
Tmt25S-2232-chimera	AmCmCmCmGmCmGmCmUmUmGmGm(TTGA)AmUmUmUmCmUmUmCmAm
h28S-3892-chimera	CmUmUmUmCmCmCmCmGmCmUmGm(ATTC)CmGmCmCmAmAmGmCmCm
Tmt25S-2611-chimera	GmCmCmCmCmAmGmCmCmAmAmAmCm(TCCC)CmAmCmCmUmGmAm
Tmt25S-2703-chimera	CmGmAmGmCmUmUmUmUmAmCmCm(CTTC)UmGmUmUmCmCmAmCmAm
Tmt25S-2755-chimera	CmGmUmUmAmGmGmCmCmAmCmGm(CTTT)CmAmCmGmGmUmUm
h28S-4443-chimera	GmCmCmGmAmCmAmUmCmGmAmAmGm(GATC)AmAmAmAmAmGmCmGmAmCmGmUm
h28S-4516-chimera	AmCmCmCmAmGmCmUmCmAmCmGmUmUm(CCCT)AmUmUmAmGmUmGmGmGmUm
p25S-3044-chimera	CmCmUmGmUmCmUmCmAmCm(GACG)GmUmCmUmAmAmAmCmCmCmAm
h28S-4563-chimera	CmAmUmCmAmUmCmAmGmUm(AGGG)UmAmAmAmAmCmUmAmAmCmCmUmGmUmCm
Tmt25S-3143-chimera	GmCmAmUmGmCmGmUmCmGmCm(TTCT)AmGmCmCmCmGmGmAmUm
Tmt18S_F1	(TACCTGGTTGATCCTGCCAG)
Tmt18S_F445	(AATCCTGACACGGGGAGGTA)
Tmt18S_F1196	(CGGGGAAACTTACCAGGTCC)
Tmt18S_R544	(CACCAGACTTGCCCTCCAAT)
Tmt18S_R1170	(GTCAAATTAAGCCGCAGGCT)
Tmt18S_R1488	(GTCGGCCAAGGCTATAAGCT)
Tmt18S_R1764	(ACCTACGGAAACCTTGTTAC)
Tmt25S_F1	(GCGACCCCAGGTCAGGC)
Tmt25S_F261	(TACGAGTCGGGTTGTTTGGG)
Tmt25S_F780	(GAAGGGTTCGAGTGTGAGCA)
Tmt25S_F1109	(ATCAAGAGCTCCAAGTGGGC)
Tmt25S_F1478	(ACTTTGAAGGCCGAAGAGGG)
Tmt25S_F1847	(AACCGCATCAGGTCTCCAAG)
Tmt25S_F2194	(TCTGCCCAGTGCTCTGAATG)
Tmt25S_F2938	(GGGAACGTGAGCTGGGTTTA)
Tmt25S_R780	(TGCTCACACTCGAACCCTTC)
Tmt25S_R1189	(GATCTAGGTTAGCGCGCAGT)
Tmt25S_R2956	(ACCTGTCTCACGACGGTCTA)
Tmt25S_R3367	(ACGAATCGGAGCGTCAAAGG)

a. Nm refers to 2'-O-methyl ribonucleotide. Deoxyribonucleotides are indicated in parentheses.

Oligonucleotide	DNA/RNA	Note
Tmt18S-270-chimera	DNA/RNA	Used for RNase H digestion to produce Fragment H1 or H2.
c18S-385-chimera	DNA/RNA	Used for RNase H digestion to produce Fragment H2 or H3.
c18S-580-chimera	DNA/RNA	Used for RNase H digestion to produce Fragment H3 or H4.
Tmt18S-831-chimera	DNA/RNA	Used for RNase H digestion to produce Fragment H4 or H5.
h18S-1210-chimera	DNA/RNA	Used for RNase H digestion to produce Fragment H5 or H6.
Tmt18S-1332-chimera	DNA/RNA	Used for RNase H digestion to produce Fragment H7 or H8.
Tmt18S-1597-chimera	DNA/RNA	Used for RNase H digestion to produce Fragment H8 or H9.
Tmt18S-1679-chimera	DNA/RNA	Used for RNase H digestion to produce Fragment H9 or H10.
Tmt25S-418-chimera	DNA/RNA	Used for RNase H digestion to produce Fragment H10 or H11.
Tmt25S-643-chimera	DNA/RNA	Used for RNase H digestion to produce Fragment H11, H12 or H14.
Tmt25S-850-chimera	DNA/RNA	Used for RNase H digestion to digest the RNase T1 fragment 28S_1764-1791 into two fragments.
h28S-1866-chimera	DNA/RNA	Used for RNase H digestion to produce Fragment H12 or H13.
Tmt25S-1521-chimera	DNA/RNA	Used for RNase H digestion to produce Fragment H13, H14 or H15.
Tmt25S-1859-chimera	DNA/RNA	Used for RNase H digestion to produce Fragment H15 or H16.
Tmt25S-2119-chimera	DNA/RNA	Used for RNase H digestion to produce Fragment H16 or H17.
Tmt25S-2232-chimera	DNA/RNA	Used for RNase H digestion to produce Fragment H18.
h28S-3892-chimera	DNA/RNA	Used for RNase H digestion to produce Fragment H17.
Tmt25S-2611-chimera	DNA/RNA	Used for RNase H digestion to produce Fragment H18 or H19.
Tmt25S-2703-chimera	DNA/RNA	Used for RNase H digestion to produce Fragment H19 or H20.
Tmt25S-2755-chimera	DNA/RNA	Used for RNase H digestion to produce Fragment H20, H21, H22 or H23.
h28S-4443-chimera	DNA/RNA	Used for RNase H digestion to produce Fragment H23 or H24.
h28S-4516-chimera	DNA/RNA	Used for RNase H digestion to produce Fragment H22, H24, H25 or H29.
p25S-3044-chimera	DNA/RNA	Used for RNase H digestion to produce Fragment H25 or H26.
h28S-4563-chimera	DNA/RNA	Used for RNase H digestion to produce Fragment H26 or H27.
Tmt25S-3143-chimera	DNA/RNA	Used for RNase H digestion to produce Fragment H27 or H28.
Tmt18S_F1	DNA	Used for PCR and sequencing primer.
Tmt18S_F445	DNA	Used for sequencing primer.
Tmt18S_F1196	DNA	Used for sequencing primer.
Tmt18S_R544	DNA	Used for sequencing primer.
Tmt18S_R1170	DNA	Used for sequencing primer.
Tmt18S_R1488	DNA	Used for sequencing primer.
Tmt18S_R1764	DNA	Used for reverse transcription, PCR and sequencing primer.
Tmt25S_F1	DNA	Used for PCR and sequencing primer.
Tmt25S_F261	DNA	Used for sequencing primer.
Tmt25S_F780	DNA	Used for sequencing primer.
Tmt25S_F1109	DNA	Used for sequencing primer.
Tmt25S_F1478	DNA	Used for sequencing primer.
Tmt25S_F1847	DNA	Used for sequencing primer.
Tmt25S_F2194	DNA	Used for sequencing primer.
Tmt25S_F2938	DNA	Used for sequencing primer.
Tmt25S_R780	DNA	Used for sequencing primer.
Tmt25S_R1189	DNA	Used for sequencing primer.
Tmt25S_R2956	DNA	Used for sequencing primer.
Tmt25S_R3367	DNA	Used for reverse transcription, PCR and sequencing primer.
