# Supplementary material for: Cryo-EM structure and rRNA modification sites of a plant ribosome
Source: Plant Commun. 2022 May 27;3(5):100342. doi: 10.1016/j.xplc.2022.100342 (PMC9483110; doi:10.1016/j.xplc.2022.100342)
Supplement: Document S2. Article plus supplemental information [file mmc3.pdf]

Plant Communications
Research Article

CellPress
Partner Journal

Cryo-EM structure and rRNA modification sites of a plant ribosome

Patrick Cottilli[1,5], Yuzuru Itoh[1,5], Yuko Nobe[2,5], Anton S. Petrov[3], Purificación Lisón[4], Masato Taoka[2,*] and Alexey Amunts[1,*]

[1]Science for Life Laboratory, Department of Biochemistry and Biophysics, Stockholm University, 17165 Solna, Sweden

[2]Department of Chemistry, Graduate School of Science, Tokyo Metropolitan University, Minami-osawa 1-1, Hachioji-shi, Tokyo 192-0397, Japan

[3]School of Chemistry and Biochemistry, Georgia Institute of Technology, Atlanta, GA, USA

[4]Instituto de Biología Molecular y Celular de Plantas, Universitat Politècnica de València (UPV) – Consejo Superior de Investigaciones Científicas (CSIC), Ciudad Politécnica de la Innovación (CPI), Valencia 46022, Spain

[5]These authors contributed equally

***Correspondence: Masato Taoka (mango@tmu.ac.jp), Alexey Amunts (amunts@scilifelab.se)**

https://doi.org/10.1016/j.xplc.2022.100342

ABSTRACT

Protein synthesis in crop plants contributes to the balance of food and fuel on our planet, which influences human metabolic activity and lifespan. Protein synthesis can be regulated with respect to changing environmental cues via the deposition of chemical modifications into rRNA. Here, we present the structure of a plant ribosome from tomato and a quantitative mass spectrometry analysis of its rRNAs. The study reveals fine features of the ribosomal proteins and 71 plant-specific rRNA modifications, and it re-annotates 30 rRNA residues in the available sequence. At the protein level, isoAsp is found in position 137 of uS11, and a zinc finger previously believed to be universal is missing from eL34, suggesting a lower effect of zinc deficiency on protein synthesis in plants. At the rRNA level, the plant ribosome differs markedly from its human counterpart with respect to the spatial distribution of modifications. Thus, it represents an additional layer of gene expression regulation, highlighting the molecular signature of a plant ribosome. The results provide a reference model of a plant ribosome for structural studies and an accurate marker for molecular ecology.

Key Words: plant, tomato, ribosome, RNA, structure

Cottilli P., Itoh Y., Nobe Y., Petrov A.S., Lisón P., Taoka M., and Amunts A. (2022). Cryo-EM structure and rRNA modification sites of a plant ribosome. Plant Comm. **3**, 100342.

INTRODUCTION

Ribosomes are fundamental to all forms of life on earth. Their activity is regulated via chemical modifications of the four rRNA species: 25S, 18S, 5.8S, and 5S. The identities and locations of these modifications have been reported for human ribosomes, and their links to disease have been established (Gilles et al., 2020). In plants, numerous small nucleolar RNAs have been characterized, suggesting plant-specific sites of rRNA 2′-*O*-ribose methylation that may contribute to the translational control of gene expression (Barneche et al., 2001). Biochemical studies further identified plant-specific mechanisms involved in ribosome assembly, localization, stress response, and stalling, with implications for antiviral immunity (Palm et al., 2019; Cheong et al., 2021). In addition, stress-triggered ribosome heterogeneity has been reported in rice (Moin et al., 2017), suggesting the functional specialization of ribosomes and the potential importance of their future engineering in crops for food security. Therefore, understanding the structure of a plant ribosome and identifying specific post-transcriptional modifications of its rRNAs may assist in biotechnological studies that aim to develop plants with a higher nutrition dose, improved fruit development, and longer-lasting quality. In particular, the tomato *Solanum lycopersicum* serves as a model for such studies (The Tomato Genome Consortium, 2012). However, despite the central role of ribosomal function, the translation apparatus in the cytosol has not been structurally determined for the Plantae kingdom (*Viridiplantae*), and the data are limited to homology models based on low-resolution reconstructions (Armache et al., 2010), although plastid (Bieri et al., 2017; Boerema et al., 2018) and mitochondrial (Tobiasson et al., 2022; Waltz et al., 2020, 2021) ribosomes have been determined.

Here, we report the structure of a plant cytosolic ribosome and a quantitative mass spectrometry analysis of its rRNAs. The study

Published by the Plant Communications Shanghai Editorial Office in association with Cell Press, an imprint of Elsevier Inc., on behalf of CSPB and CEMPS, CAS.

This is an open access article under the CC BY license (http://creativecommons.org/licenses/by/4.0/).

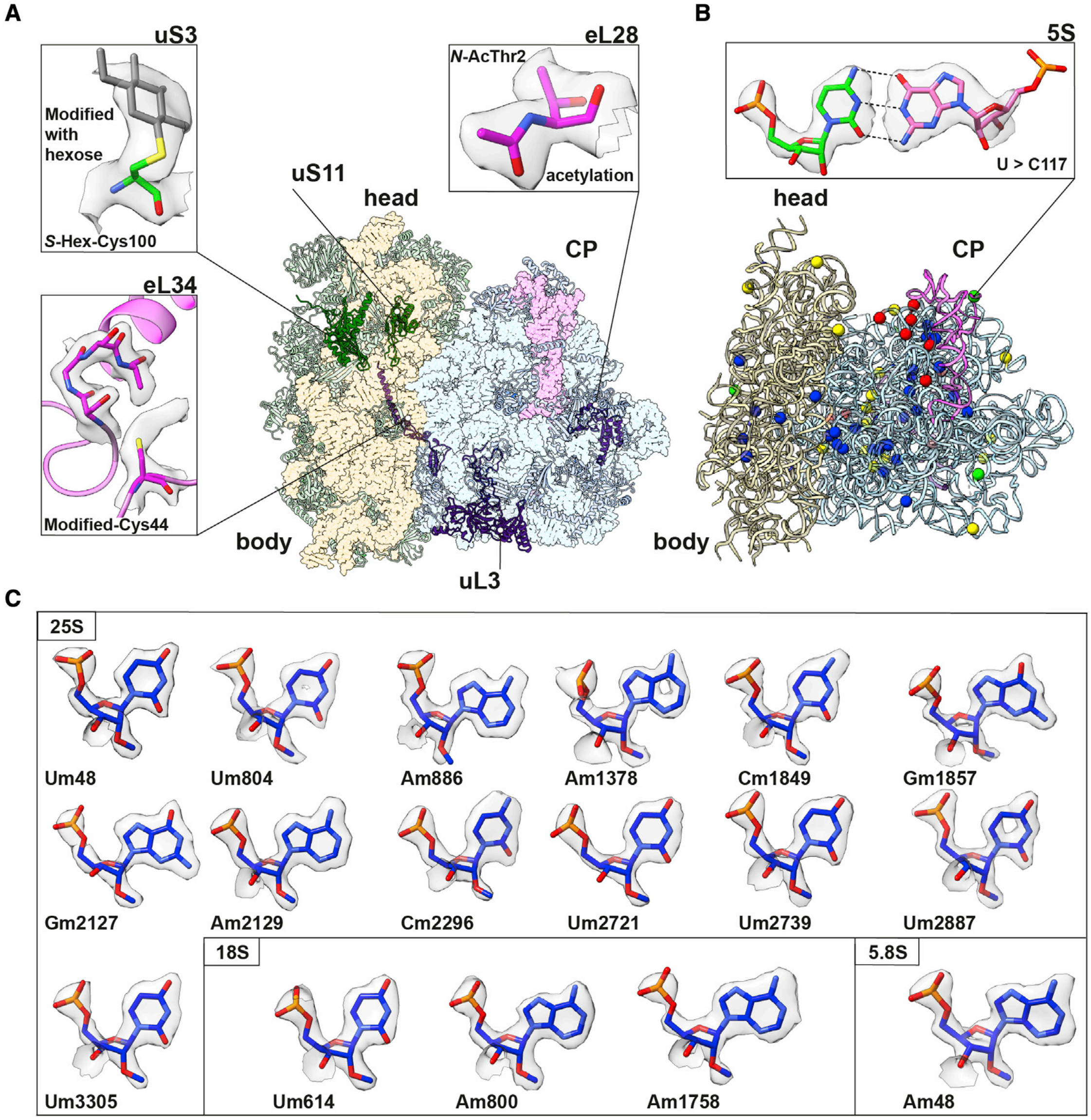

Figure 1. Structure and features of the plant ribosome.
(A) Overall structure, featuring examples of post-translational modifications.
(B) rRNA re-annotations mapped onto the 3D structure: point mutation (green), deletion (red), insertions (yellow), and plant-specific modifications (blue). One example is shown in the zoomed-in panel.
(C) Representative modifications supported by the cryo-EM density. Am, Um, Gm, and Cm are 2′-*O*-methylation modifications of the respective bases.

reveals plant-specific modifications, re-annotates rRNA residues, and provides a reference model for structural studies and molecular ecology.

RESULTS AND DISCUSSION

Structure and features of the plant ribosome

To determine the specific features and methylation sites of a plant ribosome, we investigated *S. lycopersicum* ribosomes by a combination of cryo-electron microscopy (cryo-EM) and mass spectrometry–based quantitative RNA analysis. Using the structural approach, we obtained a 2.38-Å resolution cryo-EM reconstruction (Figure 1A; Supplemental Figure 1) that allowed us to build an accurate atomic model with a minimal clash score of 2.02 (Supplemental Table 1) (Amunts 2022). The model included the elucidation of plant-specific structural features and the re-annotation of 30 rRNA residues (Supplemental Tables 2 and 3), and the correct sequence has now been deposited to GenBank with accession codes OK073662–5. Using the mass spectrometry approach, we identified 71 post-transcriptional modifications (Supplemental Tables 4–7) and mapped them onto the structure (Figure 1B). With these data, we then constructed an accurate structure-based rRNA diagram by extracting the base pairs directly from the model and mapping them onto the corrected nucleotide sequence (Supplemental Figures 2 and 3). The resulting

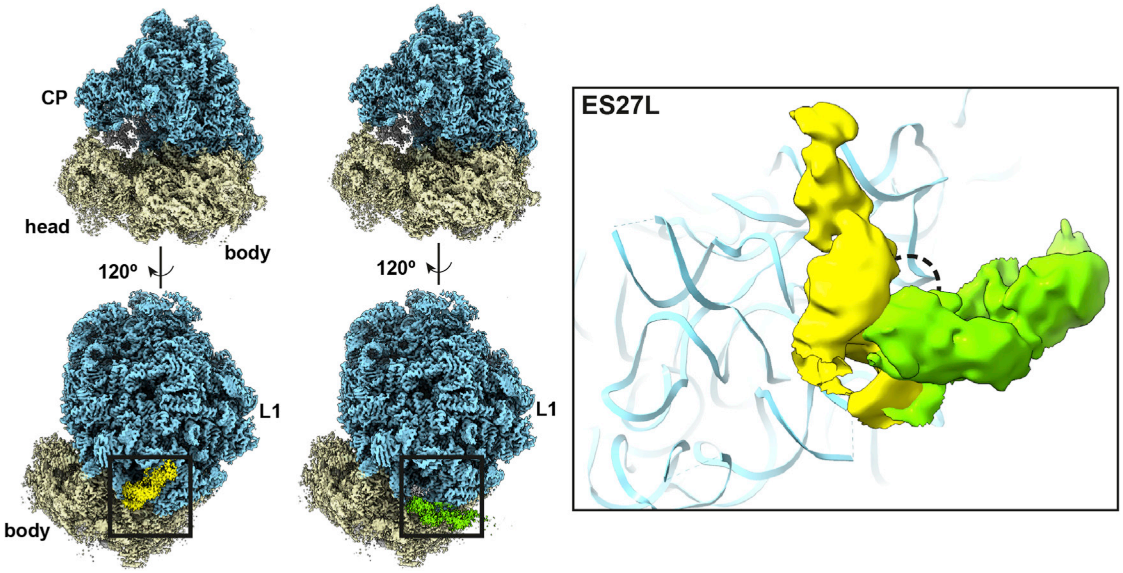

Figure 2. ES27L conformational change.
The tomato ribosome is colored by subunit. Signal subtraction and 3D classification of the peripheral regions resulted in two defined conformations of ES27L (yellow and green). The zoomed-in panel on the right shows that the conformations in different classes are related by approximately 90°.

model-based diagrams map the sequence re-annotations, newly identified non-canonical base pairing, plant expansion segments, and all experimentally detected post-transcriptional modifications.

The overall structure of a plant ribosome is generally conserved relative to its eukaryotic counterparts, including in regions that are responsible for the binding of mRNA and tRNAs. Of the total mass of approximately 3 MDa, 10% represents rRNA expansion segments (ESs) that occur on the surface. We systematically analyzed the ESs by applying signal subtraction and three-dimensional (3D) classification to the peripheral regions. We found that, although most of the ESs are intrinsically flexible, an approximately 138-nucleotide ES27L adopts two defined conformations related by approximately 90° (Figure 2). The yeast counterpart has been shown to act as an RNA scaffold that recruits the N-terminal processing enzyme MetAP, which controls the accuracy of ribosome decoding in translation fidelity (Fujii et al., 2018). Because enzymatic activities adapt to light fluctuations in plants (Martinez-Seidel et al., 2021), the defined conformations of ES27L are likely to serve a regulatory role.

Protein modifications

As the structure of the ribosome is generally conserved, we focused the analysis on high-resolution features detected in the density. On the protein level, for ribosomal protein uS11, we found an extra density corresponding with a methylene group between C_α and the backbone carbonyl group in Asp137, indicating that the γ carboxyl group instead of the α carboxyl group participates in the backbone peptide bond (Watson et al., 2020) (Figure 3A). Hence, the aspartate is converted to isoaspartate (isoAsp) via dehydration and followed by hydrolysis. In the structure, the Ser138 backbone NH hydrogen bonds with the sugar of rRNA C930, and the isoAsp backbone NH hydrogen bonds with the Pro135 backbone carbonyl group, resulting in an approximately 110° turn. A potential role for isoAsp in this position could present during the assembly, as isoAsp residues have previously been proposed to regulate protein activity by a time-dependent molecular switch (Ritz-Timme and Collins, 2002). In *E. coli*, the corresponding deamidation of asparagine in uS11 was reported during the logarithmic growth phase on the basis of its ability to serve as an exceptional methyl-accepting protein in cell extracts (David et al., 1999). This post-translational modification can be involved in spontaneously damaged proteins in plants, affecting seed viability, and because studies in *Arabidopsis* showed increased deamidation in response to stress conditions, the reactivity of residues is an important regulator (Peña et al., 2016). As deamidation occurs rapidly *in vitro*, it is difficult to detect under physiological conditions; therefore, the structural approach is informative.

For zinc finger protein eL34, whose aberrant expression in humans is associated with malignancies (Fan et al., 2017), no density corresponding to the zinc ion is found in the plant ribosome structure, and three of four cysteines that form the binding site are absent (Figure 3B; Supplemental Figure 4). In addition, we found a modification in the remaining cysteine 44 (Figure 1A), thus eliminating the zinc finger motif. A phylogenetic analysis showed that the structure-derived observation is conserved in *Viridiplantae* (Supplemental Figure 4). This suggests a lower effect of zinc deficiency on protein synthesis in plants. Our structural data are consistent with the observation that ribosome content remains unchanged in cultured tobacco plant cells with 0.09 ppm zinc in the medium (Obata and Umebayashi, 1988), whereas zinc content in rich soil is estimated at 10–30 ppm. Therefore, the zinc finger previously believed to be universal is absent from plant ribosomes, contributing to physiological functioning of plant metabolism at lower nutrient concentrations.

rRNA modifications

On the rRNA level, we identified multiple discrepancies between the cryo-EM density map and available sequences (Supplemental

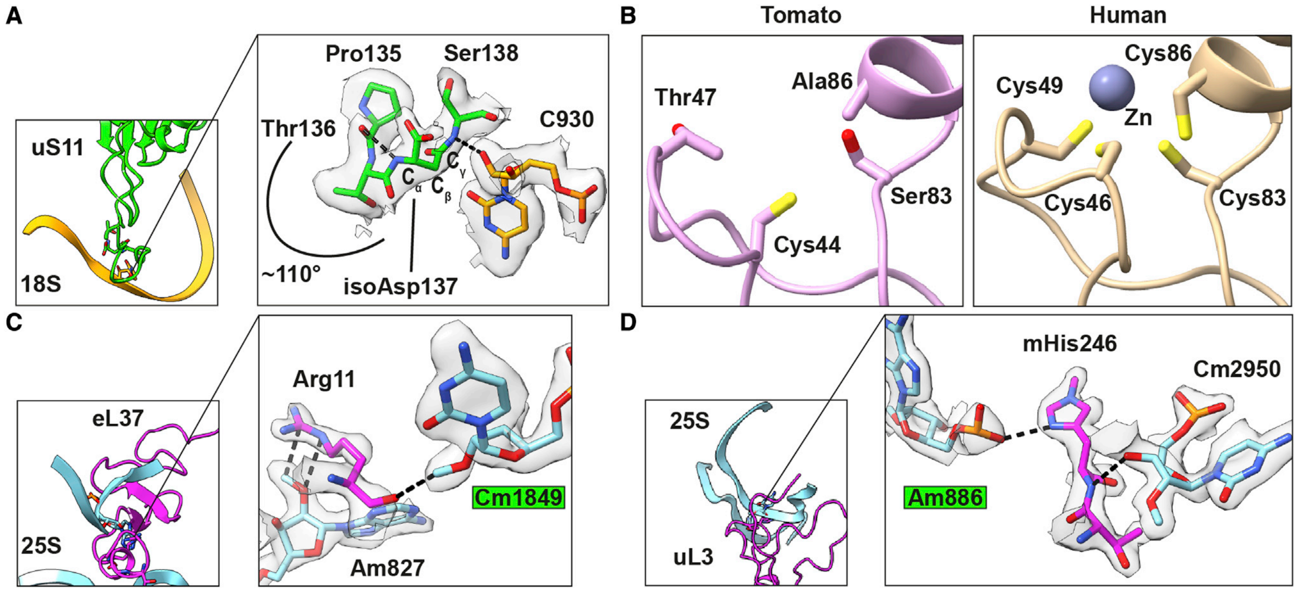

Figure 3. High-resolution features.

(A) View of uS11 shows interactions of isoAsp137, Pro135, and Ser138 with rRNA C930 that stabilize the turn in the protein backbone.

(B) Comparison of the eL34 zinc finger structure between tomato and human.

(C) View of eL37 shows the modified Cm1849 with its methyl group forming a repulsive interaction with the backbone carbonyl group of Arg11, and the modified Gm1857 interacting hydrophobically with Gly6 and Thr6, which stabilizes the helical structure.

(D) View of uL3 shows hydrogen bond interactions between the modified Am886, Cm2952, and His236.

Figure 5). Through a combination of analysis of the density map, sequencing, and alignments, we detected 9 deletions, 18 insertions, and 4 mutations in the *S. lycopersicum* rRNA (Figure 1B; Supplemental Table 2 and 3), which allowed us to correct the sequence for the model organism (Supplemental Figure 6). To confirm that discrepancies are independent of the plant cultivar, we performed multiple sequence analysis, and only position 120 in the 5S rRNA was found to vary. Next, we analyzed rRNA modifications by the quantitative stable isotope-labeled ribonucleic acid as an internal standard (SILNAS) method (Taoka et al., 2015). 2′-*O*-methylations can change in response to upstream signaling pathways (Jansson et al., 2021). Therefore, to confirm that the identified modifications represent a fundamental feature of the plant ribosome and not an intrinsic modulation, we extracted material from young establishment stages and stressed leaves in two separate experiments (Supplemental Figure 7). The consistent stoichiometry between the experiments suggests that the primary and secondary veins exhibit rRNA modifications similar to those of the lamina. Overall, 216 modifications could be assigned, 71 of which are plant specific, meaning that they are not found in other organisms (Figure 1; Supplemental Tables 4–7). Among all the assigned modifications, 89 are strongly supported by the cryo-EM density map. The position and stoichiometry of all modifications are presented in Supplemental Table 8, and a role could be proposed for some of them. For example, 2′-*O*-methylguanosine 1857 and 2′-*O*-methylcytidine 1849, together with the conserved modification 2′-*O*-methyladenosine 827, shape the N-terminal region of eL37 through hydrophobic interactions (Figure 3C). This region is involved in constructing the peptide exit tunnel. Protein eL37 is required for recruiting Nsa2 and Nog2 for 27SB pre-rRNA processing, and its repression would cause null synthesis of 25S relative to 18S rRNA (Gamalinda et al., 2013). In a similar way, 2′-*O*-methyladenosine 886 and conserved 2′-*O*-methylcytosine 2920 directly interact with the modified residue 3-methylhistidine 246 of uL3 through hydrogen bonding (Figure 3D). This modification is crucial for proper pre-rRNA processing, polysome formation, and correct ribosomal function (Malecki et al., 2021).

Because a similar analysis has also been performed for a human ribosome (Taoka et al., 2018), we compared the data (Figure 4, Supplemental Figures 2 and 3, and Supplemental Table 8). The major difference between the ribosomal structures of the two species resides in the length of their rRNA ESs. Because no structure-based 2D rRNA diagrams are available in the literature, we constructed them to expand on the comparison and mapped the modifications (Figure 4B; Supplemental Figures 2 and 3). The plant and human ribosome have a similar number of species-specific rRNA modifications in each of the ribosomal subunits. However, the two subunits differ markedly in the spatial distribution of their species-specific modifications. In the large subunit, most human-specific modifications are concentrated at the bottom, in proximity to the two largest ESs—ES3 and ES6. By contrast, in the small subunit, both plant- and human-specific modifications are scattered all over its surface.

The deposition of chemical modifications into RNA has recently emerged as a source of ribosome heterogeneity and constitutes a mechanism for rapid adaptation to changing environmental cues (Gay et al., 2021). Such adaptation is crucial for cellular homeostasis, and dysregulated RNA modification pathways in humans have been linked to tumorigenesis (Kumari et al., 2021). A recently constructed rRNA 2′O-methylation landscape of primary human breast tumors uncovered the existence of stable and variable modification sites (Marcel et al., 2020). These affect the dynamics of rRNA, resulting in a change in the

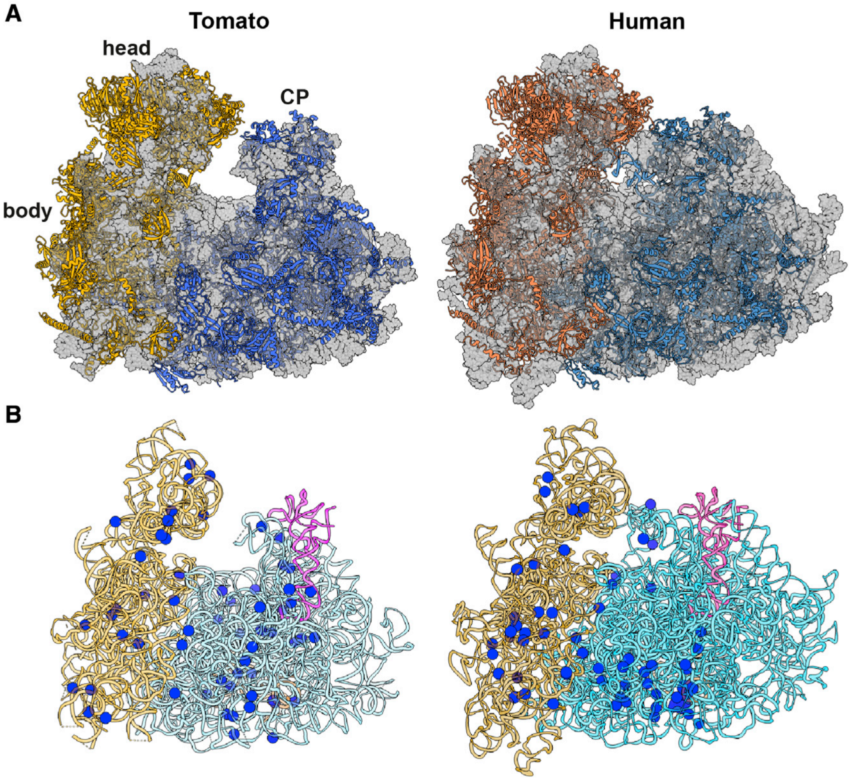

Figure 4. Side-by-side comparison of tomato and human cytosolic ribosomes.
(A) The overall structure of tomato (current work) and human (PDB 6QZP; Natchiar et al., 2017) cytosolic ribosomes.
(B) The 3D mapping specific modifications.

balance between different conformational states of the ribosomes required for translation (Khoshnevis et al., 2022). Thus, chemical modifications of ribosomal RNA represent a level of gene expression regulation. In this respect, our plant ribosome profiling that maps specific 2′O-methylations provides an additional layer of information to capture ribosomal heterogeneity in plants and offers a new molecular signature of a plant ribosome.

In conclusion, analyses of the plant ribosome provide validated information on the revised sequence of rRNA, ribosomal modifications, and high-resolution structural details with implications for protein synthesis, plant genomics, and ribosome evolution. This work also shows that high-resolution cryo-EM in combination with mass spectrometry can be used as a tool for detecting the formation of physiologically induced chemical modifications and for uncovering plant-specific features of translation. Together, these data provide a reference model of a plant ribosome for use in structural studies and an accurate source of information for biodiversity researchers.

MATERIALS AND METHODS

Sample preparation for cryo-EM

Tomato plants (*S. lycopersicum*, variety Rutgers) were cultivated in a growth chamber with 16 h fluorescent light at 30°C and 8 h darkness at 25°C. To stimulate stress conditions, plants were infected with citrus exocortis viroid by puncture (Bellés et al., 1991). Leaf tissue was collected from the apexes and frozen in liquid nitrogen. The material was then milled with a RESTCH Mixer Mill and stored at –80°C. Upon thawing, 2 mL of buffer containing 0.3 M mannitol, 20 mM HEPES-KOH (pH 7.5), 50 mM KCl, 5.0 mM Mg(OAc)$_2$, 1.0 mM DTT, EDTA-free Protease Inhibitor Cocktail (Roche), and RNaseOUT (Invitrogen) was added to 1 g of the material, and the sample was pipetted 20 times to extract the cytoplasmic fraction. After centrifugation at 1000×*g* for 2 min at 4°C, the supernatant was collected and re-centrifuged at 18,000×*g* for 10 min at 4°C to pellet debris and organelles. The supernatant was then carefully loaded onto a sucrose cushion (0.6 M sucrose, 0.50% Triton X-100, 10 mM HEPES-KOH [pH 7.5], 50 mM KOAc, 5.0 mM Mg(OAc)$_2$, 1.0 mM DTT) and centrifuged at 355,040×*g* with a TLA 120.2 rotor (Beckman) for 1 h at 4°C. The pellet was washed with resuspension buffer containing 10 mM HEPES-KOH (pH 7.5), 50 mM KOAc, 5.0 mM Mg(OAc)$_2$, and 1.0 mM DTT before resuspension. An additional centrifugation step at 18,000×*g* for 10 min at 4°C was performed to pellet aggregates and debris. The supernatant was loaded onto a 15%–30% sucrose gradient and centrifuged at 101,104×*g* with a TLS-55 rotor (Beckman) for 2.25 h at 4°C. The gradient was fractionated, the peak of the 80S ribosome was pooled, and the sample was transferred to resuspension buffer and concentrated by ultrafiltration with a Vivaspin MWCO 30,000 (Sartorius). Ribosome concentration was adjusted using A_{260} to approximately 10–20 units for cryo-EM. Grids (Quantifoil R2/1, 300 mesh, gold) were manually coated with a continuous carbon film of 3-nm thickness. Glow discharge was performed for 30 s at 20-mA intensity. Sample vitrification was performed with a Vitrobot MKIV (FEI/Thermo Fisher) with 100% humidity at 4°C with a 30-s wait time and a 3.5-s blotting time. We loaded 3 μL of the sample onto the grids.

Data collection and processing

Data were collected on a Titan Krios microscope (Thermo Fisher Scientific) operated at 300 kV with a Quantum K2 Summit camera (Gatan) using EPU software at a pixel size of 0.82–0.83 Å. Movies with 20 frames were collected with a total dose of 30.2 $e^-/\text{Å}^2$ and an exposure time of 4 s at a defocus range of 1–2.8 μm with a 0.2-μm step. Three datasets were collected, and each was processed separately in RELION 3.0.8 (Zivanov et al., 2018). Motion correction was performed with MotionCor2 (Zheng et al., 2017), and the CTF parameters were estimated by Gctf (Zhang, 2016). The particles were initially picked either by Gaussian-based autopicking in RELION or Gautomatch v0.55 and subjected to two-dimensional (2D) classification. Selected 2D class averages were used as templates for reference-based picking, followed by 2D classification in RELION 3.0.8. After several rounds of optimizations, the last picking was performed with gautomatch v0.55, obtaining 386,768 particles from dataset 1; 298,277 from dataset 2; and 329,530 from dataset 3. The final picked particles were extracted with four-time binning for the 2D classification, which was performed iteratively; the best classes were retained, and the ribosome-looking classes were further 2D classified to obtain the best classes and eventually merge them together. The merged particles were re-extracted with twice binning for 3D auto-refinement by RELION 3.0.8, followed by 3D classification to remove broken particles. We selected 106,303 good particles from dataset 1, 48,545 from dataset 2, and 31,991 from dataset 3. Selected particles were re-extracted without binning, and 3D auto-refinement was performed to create a solvent

mask. We performed 3D auto-refinement using the solvent mask for the final reconstruction of each dataset; namely, 3 Å of resolution from dataset 1, 3.3 Å from dataset 2, and 3.45 Å from dataset 3. At this point, the datasets were merged, and the processing continued in RELION 3.1 (Zivanov et al., 2020). The merged particles were 3D auto-refined using the map that resulted from dataset 1 as a reference; RELION 3.1 therefore resampled the processed particles according to the reference map used (i.e., 0.83 Å/pixel), followed by CTF refinement and Bayesian polishing. Finally, an additional step of CTF refinement was performed, followed by a 3D auto-refine and post-processing, obtaining 2.38-Å resolution with a sharpening *B*-factor of –47.277.

To improve local resolution, local masks covering 60S, 40S-body, and 40S-head were prepared. First, local-masked 3D auto-refine covering 60S was performed for the best map of 60S. Particle subtraction applying a loose mask around the 40S was performed to subtract signals outside of the mask, followed by local-masked 3D auto-refine using the 40S-body mask to obtain the best map of the 40S body. Finally, further particle subtraction applying the 40S-head mask to retain only the signal of the head was performed, followed by local-masked 3D auto-refine covering the head for its best map. To separate tRNA-bound states, an unmasked 3D classification with local-angular search from the overall 3D auto-refine was performed to separate the rotated and non-rotated conformations. A mask covering the tRNA binding sites was prepared. For each rotational state, signal subtraction applying the tRNA mask was performed to subtract outside the mask, followed by a focused 3D classification without alignment using the tRNA mask. Two tRNA-bound elongation states, A/A-P/P-E/E (36,183 particles) and A/P-P/E (66,681 particles), were obtained.

To classify the rRNA ES ES27, particles from the local-masked 60S alignment were further signal subtracted, retaining the signals inside a mask englobing the three different possible conformations found. Then, a focused 3D classification in that region with optimized parameters, τ value 150, E-step 12 Å, and 25 iterations gave three distinct classes. The signal was reverted to obtain the complete ribosome, and a 3D auto-refine was performed to create a proper solvent mask for each class, followed by a final 3D auto-refine using the solvent mask giving the best map of each class. For each of the maps, local resolution filtering using RELION 3.1 was performed, applying the sharpening *B*-factor estimated by post-processing.

Model building and refinement

Model building was performed in Coot (Emsley and Cowtan, 2004). The starting model used as a reference was the *H. sapiens* ribosome (PDB: 6EK0) (Natchiar et al., 2017). For every protein, a BLASTp search was performed at both the NCBI and Sol Genomics Network databases in case any incongruences were found. Then, each protein chain in 6EK0 PDB was used as a template in SWISS-MODEL (Waterhouse et al., 2018) to obtain the homology model for tomato. Protein and RNA models were fitted and manually adjusted to the cryo-EM maps. Ligands, metal ions, waters, and modifications were placed based on the density. Hydrogens were generated to have better clash scores, and stereochemical and *B*-factor refinements were performed using phenix.real_space_refine in the PHENIX suite. The final models were validated using MolProbity (Williams et al., 2018). Refinement statistics are given in Supplemental Table 1.

Total RNA extraction

Leaf tissue was collected, frozen, and milled as mentioned above. Total RNA was purified by adding 5 volumes, relative to the sample volume, of home-made Trizol (38% phenol saturated [pH 4.3], 0.8 M guanidine thiocyanate, 0.8 M ammonium thiocyanate, 0.1 M sodium acetate [pH 5.0], 5% glycerol) and vigorously vortexed for 10–30 s. Incubation for 5 min at room temperature was followed by the addition of 1 volume of chloroform, relative to the original sample volume, and the contents were mixed by inverting the tube 10–20 times. The mixture was then centrifuged at 12,000×*g* for 15 min at 4°C, and the aqueous phase was transferred to a new tube. At this point, 1.1 volumes, relative to the aqueous phase, of cold isopropanol were added and mixed by gently inverting the tube. After incubating for 2 h at −20°C, the RNA was pelleted at 12,000×*g* for 45 min at 4°C. After the supernatant was discarded, 1 volume, relative to the aqueous phase, of cold 70% ethanol was added to gently clean the RNA. After a 10-min incubation at 4°C, the tube was centrifuged at 12,000×*g* for 10 min at 4°C, and the supernatant was discarded. Finally, the pellet was allowed to dry at 4°C and resuspended with 35 μL of RNase-free water.

Multiple sequence alignment, phylogenetic tree, and figures

The multiple sequence alignments were performed with ClustalW and the phylogenetic tree reconstruction with MEGA7 (maximum likelihood method with 1000 bootstrap replications, gap opening penalty of 2) (Kumar et al., 2016). All the sequences were retrieved from the first BLASTp hit at NCBI, except for that of *Picea abies*, which was only possible to find at "congenie.org" (Nystedt et al., 2013). The sequences used for multiple sequence alignment and phylogenetic tree construction are found under their respective figures. All figures were prepared with ChimeraX (Goddard et al., 2018) and Coot (Emsley and Cowtan, 2004).

Sequencing of 18S and 25S rRNAs

Single-stranded cDNA was synthesized from the total RNA using a cDNA synthesis kit (SuperScript III First-Strand Synthesis System, Thermo Fisher Scientific) with the primer Tmt18S_R1764 or Tmt25S_R3367 (Supplemental Table 9). The cDNA was amplified by PCR using the single-stranded cDNA as a template and synthetic oligonucleotides as primers (Supplemental Table 9). The PCR product was directly sequenced by the Sanger method using the sequencing primers (Supplemental Table 9).

rRNA purification

The total RNA was applied to a reversed-phase LC column (PLRP-S 4000 Å, 4.6 × 150 mm, 10 μm, Agilent Technologies), and the rRNAs were eluted with a 120-min linear gradient of 10.8%–13.2% (v/v) acetonitrile in 100 mM TEAA (pH 7.0) and 0.1 mM diammonium phosphate at a flow rate of 200 μL/min at 60°C while measuring the eluate at A260 (Yamauchi et al., 2013). The 5.8S, 18S, and 25S rRNA fractions of the eluate were used directly for the liquid chromatography/tandem mass spectrometry (LC-MS) analysis. The 5S rRNA fraction was further purified by reversed-phase LC using a column (PLRP-S 300 Å, 2.1 × 200 mm, 3 μm, Agilent Technologies) with a 120-min linear gradient of 11.8%–14.2% (v/v) acetonitrile in 100 mM TEAA (pH 7.0) and 0.1 mM diammonium phosphate at a flow rate of 100 μL/min at 60°C.

Sequence-specific RNase H cleavage of rRNA

The purified rRNA (1 pmol) was digested with 5 U RNase H (Takara Bio) at 42°C for 1 h, guided by synthetic RNA/DNA hybrids complementary to the duplex cleavage sites (5 pmol, Supplemental Table 9) in 20 μL of 40 mM Tris–HCl (pH 7.7), 0.25 mM $MgCl_2$, 1 mM DTT, and 4% glycerol. Before adding the enzyme, the sample was denatured at 65°C for 10 min. The reaction was stopped by adding 0.5 μL of 0.1 M EDTA, and the resulting fragments were separated by polyacrylamide gel containing 8 M urea. The gel was stained with SYBR Gold (Invitrogen) for 1 min, and the gel pieces containing RNA bands were excised from the gel and cut into small pieces. The RNA fragment was extracted by soaking the gel pieces in 80 μL of 20 mM triammonium citrate containing 4 M urea for 1 h. The extraction was carried out two times, and the extracts were combined and passed through a centrifugal filter unit equipped with a polyvinylidene fluoride membrane (Ultrafree-MC, Millipore). The RNAs in the eluate were finally purified by reversed-phase LC on a PLRP-S 300 Å column (2.1 × 100 mm, 3 μm, Agilent Technologies) as described previously (Yamauchi et al., 2013; Taoka et al., 2010).

LC-MS, MS/MS, and MS/MS/MS analysis and database search of RNA fragments

The rRNA was digested with RNase T1 (Worthington) or A (Sigma-Aldrich) as described previously (Taoka et al., 2010). The nucleolytic RNA fragments were analyzed with a direct nanoflow LC-MS system as described previously (Nakayama et al., 2015). The LC eluate was sprayed online at −1.3 kV with the aid of a spray-assisting device (Nakayama et al., 2019) to a Q Exactive Plus mass spectrometer (Thermo Fisher Scientific) in negative ion mode. Other settings for MS, MS/MS, and MS/MS/MS were as described previously (Yamauchi et al., 2016; Nakayama et al., 2019). Ariadne was used for database searches and assignment of MS/MS RNA spectra (Nakayama et al., 2009). A composite of *S. lycopersicum* cytosolic (5S, 5.8S, 18S, and 25S) and chloroplastic (4.5S, 5S, 16S, and 23S) rRNA sequences was used as a database. The following default search parameters for Ariadne were used: maximum number of missed cleavages, 2; variable modification parameters, 3 modifications, including monomethylation, dimethylation, acetylation, and methylaminocarboxypropylation per RNA fragment for any residue; RNA mass tolerance, ±5 ppm; and MS/MS tolerance, ±20 ppm.

Internal standard RNAs and SILNAS-based quantitation of the stoichiometry of post-transcriptional modification

The plasmids encoding tomato 18S and 25S rRNAs with the T7 promotor were purchased from Twist Bioscience. To synthesize RNA, 2 μg of the plasmid DNA was linearized with NotI and transcribed using a MEGAscript T7 kit (Thermo Fisher Scientific). When RNA was synthesized, guanosine-$^{13}C_{10}$ 5′-triphosphate (Sigma-Aldrich), cytidine-$^{13}C_9$ 5′-triphosphate, or uridine-$^{13}C_9$ 5′-triphosphate (Santa Cruz Biotechnology) solution was used instead of the respective 5′-triphosphate reagent that contained carbons with a natural isotope distribution. The RNA was precipitated in ethanol, solubilized in nuclease-free water, and purified further by reversed-phase LC as described above. SILNAS-based quantitation was performed as described previously (Taoka et al., 2018; Nakayama et al., 2009). In brief, RNA (approximately 100 fmol) from natural sources with a natural isotope distribution was mixed with an equal amount of synthetic RNA transcribed *in vitro* with ^{13}C-labeled guanosine and digested with RNase T1. For the RNA transcribed with $^{13}C_9$-labeled cytidine and uridine, RNase A was used as the digestion enzyme. The 1:1 RNA mixing was performed based on measurement of the absorbance at 260 nm and ensured later by a correction factor obtained experimentally. After obtaining the LC-MS spectrum of the digested RNA mixture, the stoichiometry of RNA modification at each site was estimated by the Ariadne program designed for SILNAS (Taoka et al., 2018). The results were confirmed by manual inspection of the original MS spectrum to examine whether the estimates were based on uncontaminated MS signals (Supplemental Table 8). The masses of RNA fragments and a-, c-, w-, and y-series ions were calculated with Ariadne (http://ariadne.riken.jp/). The cyanoethylation method was used for pseudouridine identification in the rRNAs.

Generation of structure-derived rRNA 2D diagrams

The secondary structure diagrams for *S. lycopersicum* was initially generated using the template-based approach implemented in the R2DT webserver (Sweeney et al., 2021). The 60S and 40S rRNAs were templated from layouts of *S. cerevisiae* provided by RiboVision (Bernier et al., 2014). The canonical Watson–Crick base pairs were extracted from the experimental 3D structures described in the current study using the DSSR package (Lu et al., 2015). The layouts were manually adjusted, accounting for the 3D derived base pairing, using XRNA-GT https://github.com/LDWLab/XRNA-GT. The secondary structure diagrams for *H. sapiens* were obtained from RiboVision. For both *S. lycopersicum* and *H. sapiens* secondary structure diagrams, the data mapping for the ESs and experimentally detected post-transcriptional modifications was performed in RiboVision. The final adjustments to the layouts (labeling and annotations) were performed in Adobe Illustrator.

DATA AND CODE AVAILABILITY

All data produced or analyzed in this study are included in the main text or the supplementary materials. The cryo-EM density maps and atomic coordinates have been deposited in the Electron Microscopy Data Bank (EMDB) and Protein Data Bank (PDB) under accession numbers EMD-14001, EMD-14002, EMD-14003, EMD-14004, EMD-32404, EMD-14051, and EMD-14052 and 7QIW, 7QIX, 7QIY, and 7QIZ. The rRNA sequence FASTA files have been deposited in GenBank under accession numbers OK073662 for 25S, OK073663 for 18S, OK073664 for 5.8S, and OK073665 for 5S.

SUPPLEMENTAL INFORMATION

Supplemental information is available at *Plant Communications Online*.

FUNDING

This work was supported by the Swedish Foundation for Strategic Research (ARC19:0051), the Knut and Alice Wallenberg Foundation (2018.0080), the EMBO Young Investigator Program, and a NASA award (80NSSC18K1139 to A.S.P.).

AUTHOR CONTRIBUTIONS

P.C. and Y.I. purified the sample, collected cryo-EM data, and built the model under the supervision of A.A.; Y.N. and M.T. performed mass spectrometry and data analysis; A.S.P. constructed RNA diagrams; P.L. supervised the project; P.C., Y.I., and A.A. wrote the manuscript.

ACKNOWLEDGMENTS

Cryo-EM data were collected at the SciLifeLab cryo-EM facility (funded by KAW, EPS, and Kempe Foundations) and the Diamond Light Source (funded by the Wellcome Trust, MRC, and BBSRC) under proposal number BI21643-4. No conflict of interest declared.

Received: January 21, 2022
Revised: April 7, 2022
Accepted: May 25, 2022
Published: May 27, 2022

SUPPORTING CITATIONS

Additional references in Supplemental information: Itoh et al., 2022a; Itoh et al., 2022b.

REFERENCES

Amunts, A. (2022). The revolution evolution. Nat. Plants **8**:14–17.

Armache, J.P., Jarasch, A., Anger, A.M., Villa, E., Becker, T., Bhushan, S., and Beckmann, R. (2010). Cryo-EM structure and rRNA model of a translating eukaryotic 80S ribosome at 5.5-Å resolution. Proc. Natl. Acad. Sci. U S A **107**:19748–19753.

Barneche, F., Gaspin, C., Guyot, R., and Echeverría, M. (2001). Identification of 66 box C/D snoRNAs in Arabidopsis thaliana: extensive gene duplications generated multiple isoforms predicting new ribosomal RNA 2′-O-methylation sites. J. Mol. Biol. **311**:57–73.

Bellés, J.M., Carbonell, J., and Conejero, V. (1991). Polyamines in plants infected by citrus exocortis viroid or treated with silver ions and ethephon. Plant Physiol. **96**:1053–1059.

Bernier, C.R., Petrov, A.S., Waterbury, C.C., Jett, J., Li, F., Freil, L.E., and Williams, L.D. (2014). RiboVision suite for visualization and analysis of ribosomes. Faraday Discuss. **169**:195–207.

Bieri, P., Leibundgut, M., Saurer, M., Boehringer, D., and Ban, N. (2017). The complete structure of the chloroplast 70S ribosome in complex with translation factor pY. EMBO J. **36**:475–486.

Boerema, A.P., Aibara, S., Paul, B., Tobiasson, V., Kimanius, D., Forsberg, B.O., and Amunts, A. (2018). Structure of the chloroplast ribosome with chl-RRF and hibernation-promoting factor. Nature Plants **4**:212–217.

Cheong, B.E., Beine-Golovchuk, O., Gorka, M., Ho, W.W.H., Martinez-Seidel, F., Firmino, A.A.P., and Kopka, J. (2021). Arabidopsis REI-LIKE proteins activate ribosome biogenesis during cold acclimation. Sci. Rep. **11**:1–25.

David, C.L., Keener, J., and Aswad, D.W. (1999). Isoaspartate in ribosomal protein S11 of Escherichia coli. J. Bacteriol. **181**:2872–2877.

Emsley, P., and Cowtan, K. (2004). Coot: model-building tools for molecular graphics. Acta Crystallogr. Sect. D Biol. Crystallogr. **60**:2126–2132.

Fan, H., Li, J., Jia, Y., Wu, J., Yuan, L., Li, M., and Xu, B. (2017). Silencing of ribosomal protein L34 (RPL34) inhibits the proliferation and invasion of esophageal cancer cells. Oncol. Res. **25**:1061.

Fujii, K., Susanto, T.T., Saurabh, S., and Barna, M. (2018). Decoding the function of expansion segments in ribosomes. Mol. Cell **72**:1013–1020.

Gamalinda, M., Jakovljevic, J., Babiano, R., Talkish, J., de la Cruz, J., and Woolford, J.L., Jr. (2013). Yeast polypeptide exit tunnel ribosomal proteins L17, L35 and L37 are necessary to recruit late-assembling factors required for 27SB pre-rRNA processing. Nucleic Acids Res. **41**:1965–1983.

Gay, D.M., Lund, A.H., and Jansson, M.D. (2021). Translational control through ribosome heterogeneity and functional specialization. Trends Biochem. Sci. **47**:66–81.

Gilles, A., Frechin, L., Natchiar, K., Biondani, G., Loeffelholz, O.V., Holvec, S., and Peyron, J.F. (2020). Targeting the human 80S ribosome in cancer: from structure to function and drug design for innovative adjuvant therapeutic strategies. Cells **9**:629.

Goddard, T.D., Huang, C.C., Meng, E.C., Pettersen, E.F., Couch, G.S., Morris, J.H., and Ferrin, T.E. (2018). UCSF ChimeraX: meeting modern challenges in visualization and analysis. Protein Sci. **27**:14–25.

Itoh, Y., Khawaja, A., Singh, V., Naschberger, A., Rorbach, J., and Amunts, A. (2022a). Structural basis of streptomycin off-target binding to human mitoribosome. bioRxiv. https://doi.org/10.1101/2022.02.02.478878.

Itoh, Y., Khawaja, A., Laptev, I., et al. (2022b). Mechanism of mitoribosomal small subunit biogenesis and preinitiation. Nature **606**:603–608.

Jansson, M.D., Häfner, S.J., Altinel, K., Tehler, D., Krogh, N., Jakobsen, E., and Lund, A.H. (2021). Regulation of translation by site-specific ribosomal RNA methylation. Nat. Struct. Mol. Biol. **28**:889–899.

Khoshnevis, S., Dreggors-Walker, R.E., Marchand, V., Motorin, Y., and Ghalei, H. (2022). Ribosomal RNA 2′-O-methylations regulate translation by impacting ribosome dynamics. Proc. Natl. Acad. Sci. U S A **119**. e2117334119.

Kumar, S., Stecher, G., and Tamura, K. (2016). MEGA7: molecular evolutionary genetics analysis version 7.0 for bigger datasets. Mol. Biol. Evol. **33**:1870–1874.

Kumari, K., Groza, P., and Aguilo, F. (2021). Regulatory roles of RNA modifications in breast cancer. NAR Cancer **3**:zcab036.

Lu, X.J., Bussemaker, H.J., and Olson, W.K. (2015). DSSR: an integrated software tool for dissecting the spatial structure of RNA. Nucleic Acids Res. **43**:e142.

Malecki, J.M., Odonohue, M.F., Kim, Y., Jakobsson, M.E., Gessa, L., Pinto, R., and Falnes, P.Ø. (2021). Human METTL18 is a histidine-specific methyltransferase that targets RPL3 and affects ribosome biogenesis and function. Nucleic Acids Res. **49**:3185–3203.

Marcel, V., Kielbassa, J., Marchand, V., Natchiar, K.S., Paraqindes, H., Nguyen Van Long, F., and Diaz, J.J. (2020). Ribosomal RNA 2′ O-methylation as a novel layer of inter-tumour heterogeneity in breast cancer. NAR Cancer **2**:zcaa036.

Martinez-Seidel, F., Suwanchaikasem, P., Nie, S., Leeming, M.G., Firmino, A.A.P., Williamson, N.A., and Boughton, B.A. (2021). Membrane-enriched proteomics link ribosome accumulation and proteome reprogramming with cold acclimation in barley root meristems. Front. Plant Sci. **12**:656683.

Moin, M., Bakshi, A., Madhav, M.S., and Kirti, P.B. (2017). Expression profiling of ribosomal protein gene family in dehydration stress responses and characterization of transgenic rice plants overexp ressing RPL23A for water-use efficiency and tolerance to drought and salt stresses. Front. Chem. **5**:97.

Nakayama, H., Akiyama, M., Taoka, M., Yamauchi, Y., Nobe, Y., Ishikawa, H., and Isobe, T. (2009). Ariadne: a database search engine for identification and chemical analysis of RNA using tandem mass spectrometry data. Nucleic Acids Res. **37**:e47.

Nakayama, H., Yamauchi, Y., Taoka, M., and Isobe, T. (2015). Direct identification of human cellular MicroRNAs by nanoflow liquid chromatography–high-resolution tandem mass spectrometry and database searching. Anal. Chem. **87**:2884–2891.

Nakayama, H., Yamauchi, Y., Nobe, Y., Sato, K., Takahashi, N., Shalev-Benami, M., and Taoka, M. (2019). Method for direct mass-spectrometry-based identification of monomethylated RNA nucleoside positional isomers and its application to the analysis of Leishmania rRNA. Anal. Chem. **91**:15634–15643.

Natchiar, S.K., Myasnikov, A.G., Kratzat, H., Hazemann, I., and Klaholz, B.P. (2017). Visualization of chemical modifications in the human 80S ribosome structure. Nature **551**:472–477.

Nystedt, B., Street, N.R., Wetterbom, A., Zuccolo, A., Lin, Y.C., Scofield, D.G., and Jansson, S. (2013). The Norway spruce genome sequence and conifer genome evolution. Nature **497**:579–584.

Obata, H., and Umebayashi, M. (1988). Effect of zinc deficiency on protein synthesis in cultured tobacco plant cells. Soil Sci. Plant Nutr. **34**:351–357.

Palm, D., Streit, D., Shanmugam, T., Weis, B.L., Ruprecht, M., Simm, S., and Schleiff, E. (2019). Plant-specific ribosome biogenesis factors in Arabidopsis thaliana with essential function in rRNA processing. Nucleic Acids Res. **47**:1880–1895.

Peña, C., Schütz, S., Fischer, U., Chang, Y., and Panse, V.G. (2016). Prefabrication of a ribosomal protein subcomplex essential for eukaryotic ribosome formation. Elife **5**:e21755.

Ritz-Timme, S., and Collins, M.J. (2002). Racemization of aspartic acid in human proteins. Ageing Res. Rev. **1**:43–59.

Sweeney, B.A., Hoksza, D., Nawrocki, E.P., Ribas, C.E., Madeira, F., Cannone, J.J., and Petrov, A.I. (2021). R2DT is a framework for predicting and visualising RNA secondary structure using templates. Nat. Commun. **12**:1–12.

Taoka, M., Ikumi, M., Nakayama, H., Masaki, S., Matsuda, R., Nobe, Y., and Isobe, T. (2010). In-gel digestion for mass spectrometric characterization of RNA from fluorescently stained polyacrylamide gels. Anal. Chem. **82**:7795–7803.

Taoka, M., Nobe, Y., Hori, M., Takeuchi, A., Masaki, S., Yamauchi, Y., and Isobe, T. (2015). A mass spectrometry-based method for comprehensive quantitative determination of post-transcriptional RNA modifications: the complete chemical structure of Schizosa ccharomyces pombe ribosomal RNAs. Nucleic Acids Res. **43**:e115.

Taoka, M., Nobe, Y., Yamaki, Y., Sato, K., Ishikawa, H., Izumikawa, K., and Isobe, T. (2018). Landscape of the complete RNA chemical modifications in the human 80S ribosome. Nucleic Acids Res. **46**:9289–9298.

The Tomato Genome Consortium. (2012). The tomato genome sequence provides insights into fleshy fruit evolution. Nature **485**:635–641. https://doi.org/10.1038/nature11119.

Tobiasson, V., Berzina, I., and Amunts, A. (2022). Structure of a mitochondrial ribosome with fragmented rRNA in complex with membrane-targeting elements. Nature Communictions. In press.

Waltz, F., Soufari, H., Bochler, A., Giegé, P., and Hashem, Y. (2020). Cryo-EM structure of the RNA-rich plant mitochondrial ribosome. Nat. Plants **6**:377–383.

Waltz, F., Salinas-Giegé, T., Englmeier, R., Meichel, H., Soufari, H., Kuhn, L., and Hashem, Y. (2021). How to build a ribosome from RNA fragments in Chlamydomonas mitochondria. Nat. Commun. **12**:1–15.

Waterhouse, A., Bertoni, M., Bienert, S., Studer, G., Tauriello, G., Gumienny, R., and Schwede, T. (2018). SWISS-MODEL: homology modelling of protein structures and complexes. Nucleic Acids Res. **46**:W296–W303.

Watson, Z.L., Ward, F.R., Méheust, R., Ad, O., Schepartz, A., Banfield, J.F., and Cate, J.H. (2020). Structure of the bacterial ribosome at 2 Å resolution. Elife **9**:e60482.

Williams, C.J., Headd, J.J., Moriarty, N.W., Prisant, M.G., Videau, L.L., Deis, L.N., and Richardson, D.C. (2018). MolProbity: more and better reference data for improved all-atom structure validation. Protein Sci. **27**:293–315.

Yamauchi, Y., Taoka, M., Nobe, Y., Izumikawa, K., Takahashi, N., Nakayama, H., and Isobe, T. (2013). Denaturing reversed phase liquid chromatographic separation of non-coding ribonucleic acids on macro-porous polystyrene-divinylbenzene resins. J. Chromatogr. A **1312**:87–92.

Yamauchi, Y., Nobe, Y., Izumikawa, K., Higo, D., Yamagishi, Y., Takahashi, N., and Taoka, M. (2016). A mass spectrometry-based method for direct determination of pseudouridine in RNA. Nucleic Acids Res. **44**:e59.

Zhang, K. (2016). Gctf: real-time CTF determination and correction. J. Struct. Biol. **193**:1–12.

Zheng, S.Q., Palovcak, E., Armache, J.P., Verba, K.A., Cheng, Y., and Agard, D.A. (2017). MotionCor2: anisotropic correction of beam-induced motion for improved cryo-electron microscopy. Nat. Methods **14**:331–332.

Zivanov, J., Nakane, T., Forsberg, B.O., Kimanius, D., Hagen, W.J., Lindahl, E., and Scheres, S.H. (2018). New tools for automated high-resolution cryo-EM structure determination in RELION-3. Elife **7**:e42166.

Zivanov, J., Nakane, T., and Scheres, S.H. (2020). Estimation of high-order aberrations and anisotropic magnification from cryo-EM data sets in RELION-3.1. IUCrJ **7**:253–267.

Plant Communications, Volume *3*

Supplemental information

Cryo-EM structure and rRNA modification sites of a plant ribosome

Patrick Cottilli, Yuzuru Itoh, Yuko Nobe, Anton S. Petrov, Purificación Lisón, Masato Taoka, and Alexey Amunts

Supplemental Information:

Cryo-EM structure and rRNA modification sites of a plant ribosome

Patrick Cottilli[1†], Yuzuru Itoh[1†], Yuko Nobe[2†], Anton S. Petrov[3], Purificación Lisón[4], Masato Taoka[2*], Alexey Amunts[1*]

[1] Science for Life Laboratory, Department of Biochemistry and Biophysics, Stockholm University, 17165 Solna, Sweden.

[2] Department of Chemistry, Graduate School of Science, Tokyo Metropolitan University, Minami-osawa 1-1, Hachioji-shi, Tokyo 192-0397, Japan.

[3] School of Chemistry and Biochemistry, Georgia Institute of Technology, Atlanta, GA.

[4] Instituto de Biología Molecular y Celular de Plantas. Universitat Politècnica de València (UPV) – Consejo Superior de Investigaciones Científicas (CSIC). Ciudad Politécnica de la Innovación (CPI), Valencia 46022, Spain.

† These authors contributed equally to this work.

* To whom correspondence should be addressed: mango@tmu.ac.jp, amunts@scilifelab.se

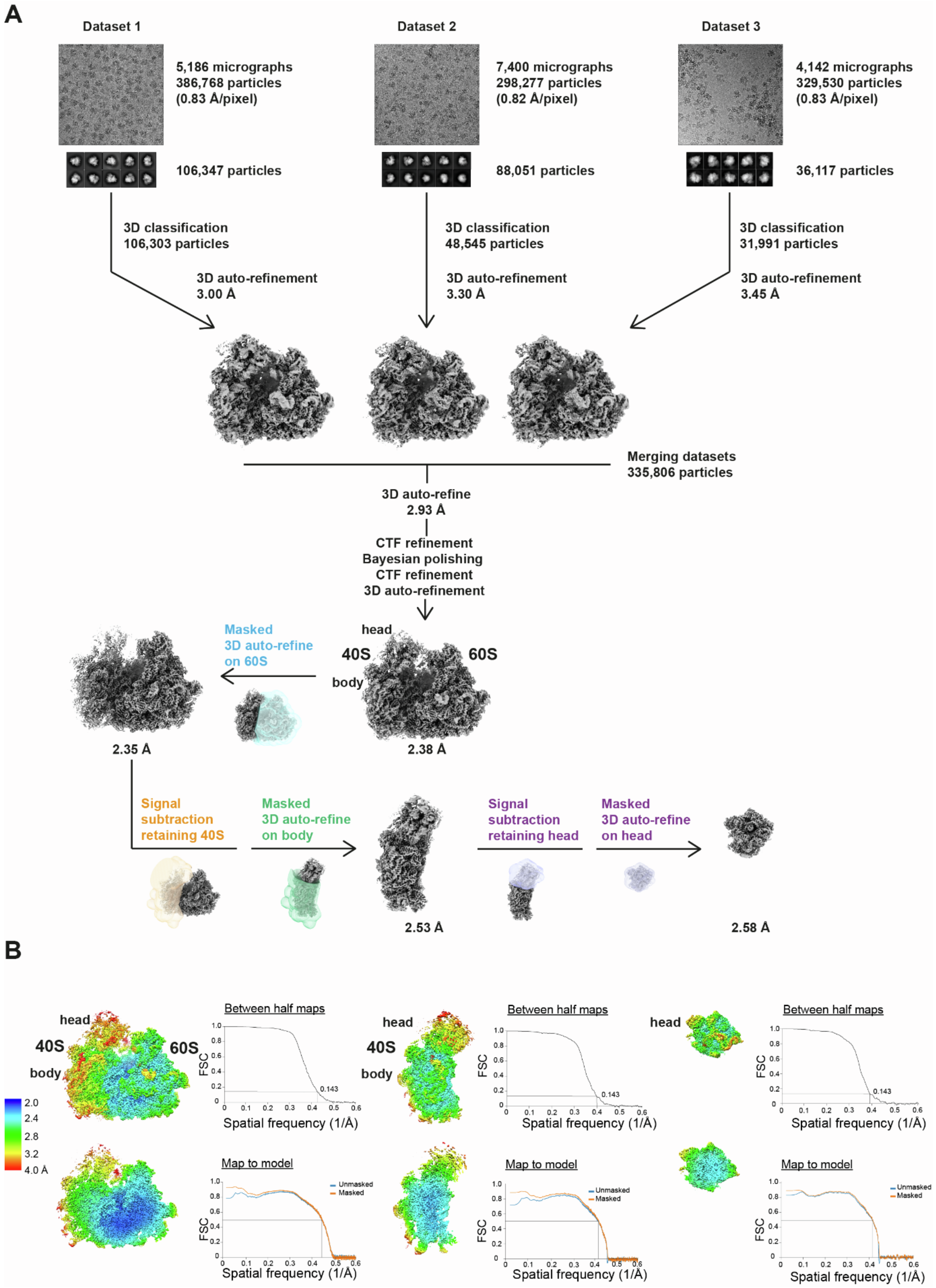

Supplementary Figure S1: Cryo-EM data collection and processing. (a) Processing scheme for the 60S, the 40S body and head. Representative micrographs and 2D class averages are shown. (b) Maps are colored by local resolution, isosurface (upper) and cut through (lower). Fourier shell correlation (FSC) plot between two half maps (threshold 0.143) and that of map-to-model (threshold 0.50) are shown for each map. The representative micrographs reflect prep-to-prep variability.

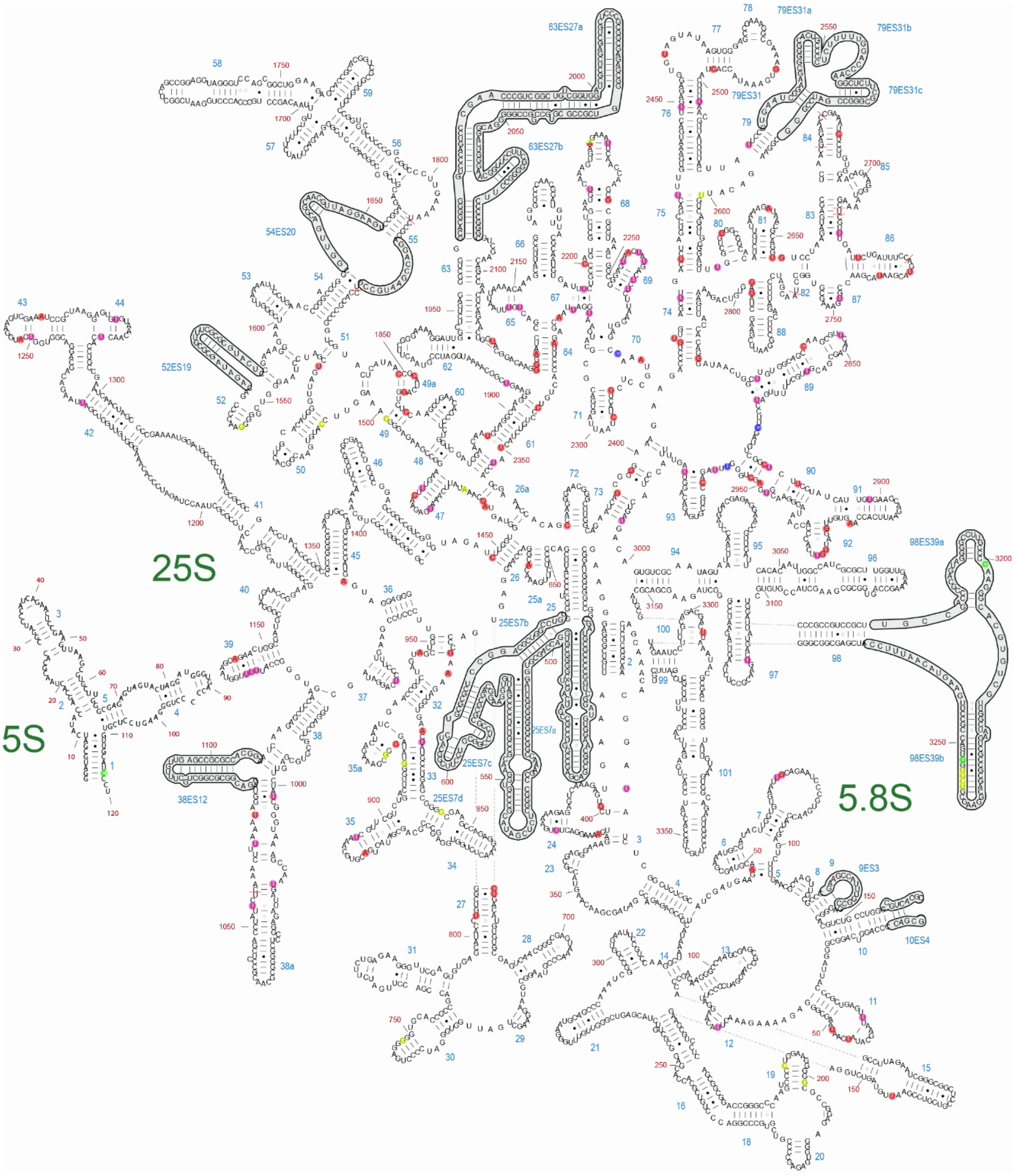
25S
5S
5.8S

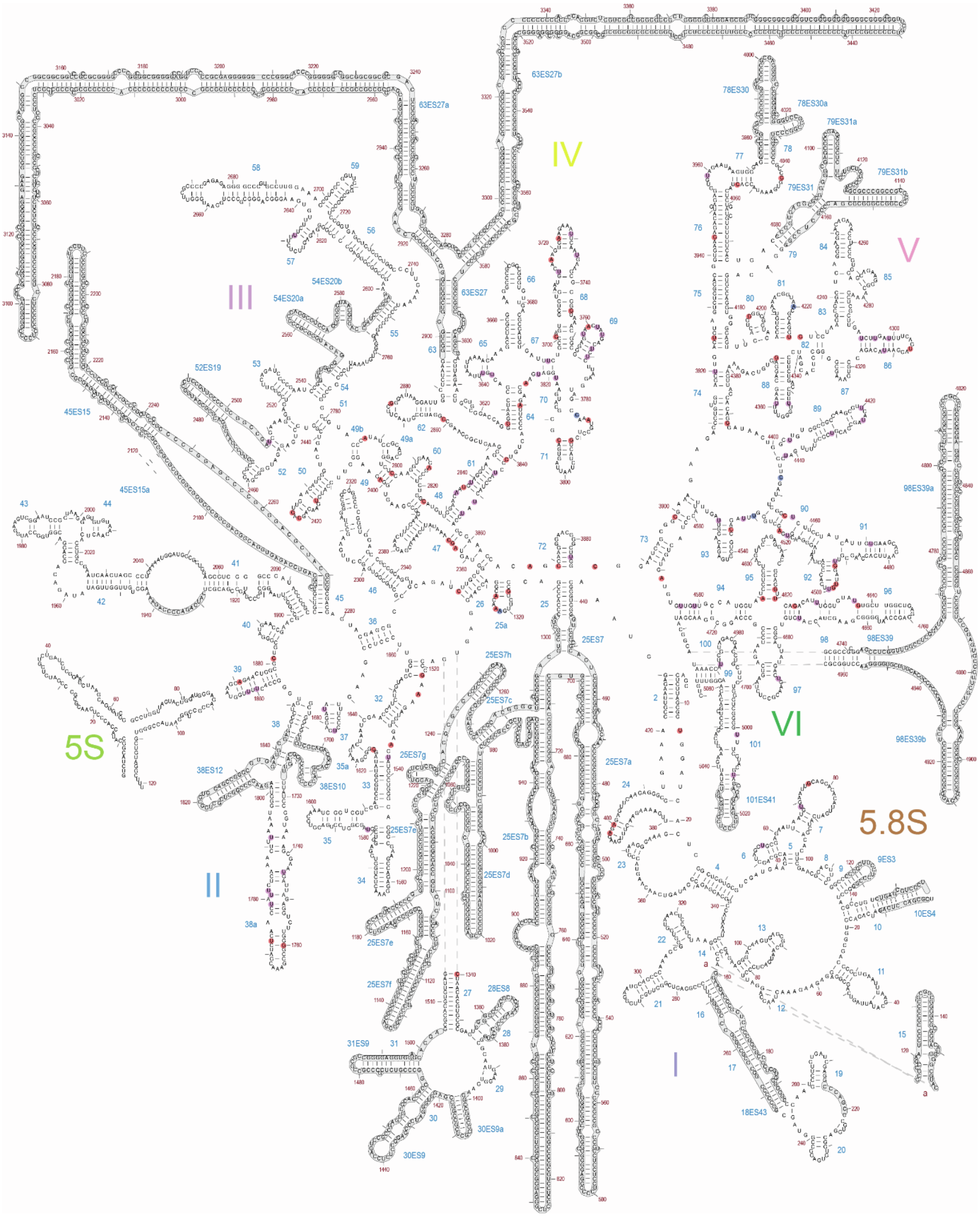

Supplementary Figure S2: *S. lycopersicum* (top) and *H. sapiens* (bottom) structure-derived rRNA 2D diagrams for the 25S rRNA. Base-pair interactions of canonical Watson-Crick pairs, standard G-U pairs and other local non-canonical ones are marked as dashes, black bullets and white bullets, respectively. Expansion segments are encircled and highlighted with grey. 2′-*O*-methylations are red, base modification blue, pseudouridines magenta, insertions yellow, point mutations green, and deletions are red lines.

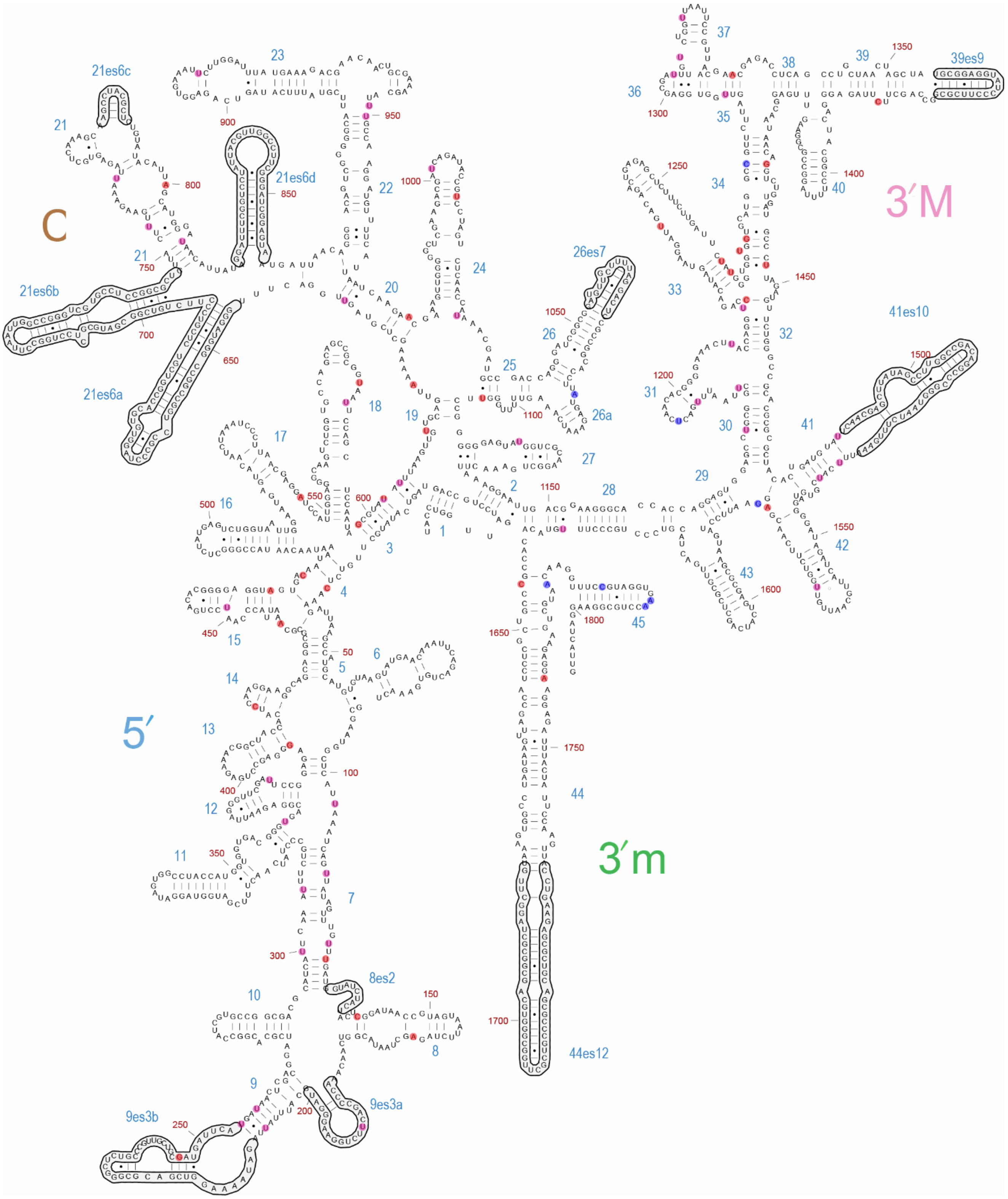

C
5′
3′M
3′m
21es6c
21es6d
21es6b
21es6a
26es7
39es9
41es10
44es12
8es2
9es3a
9es3b

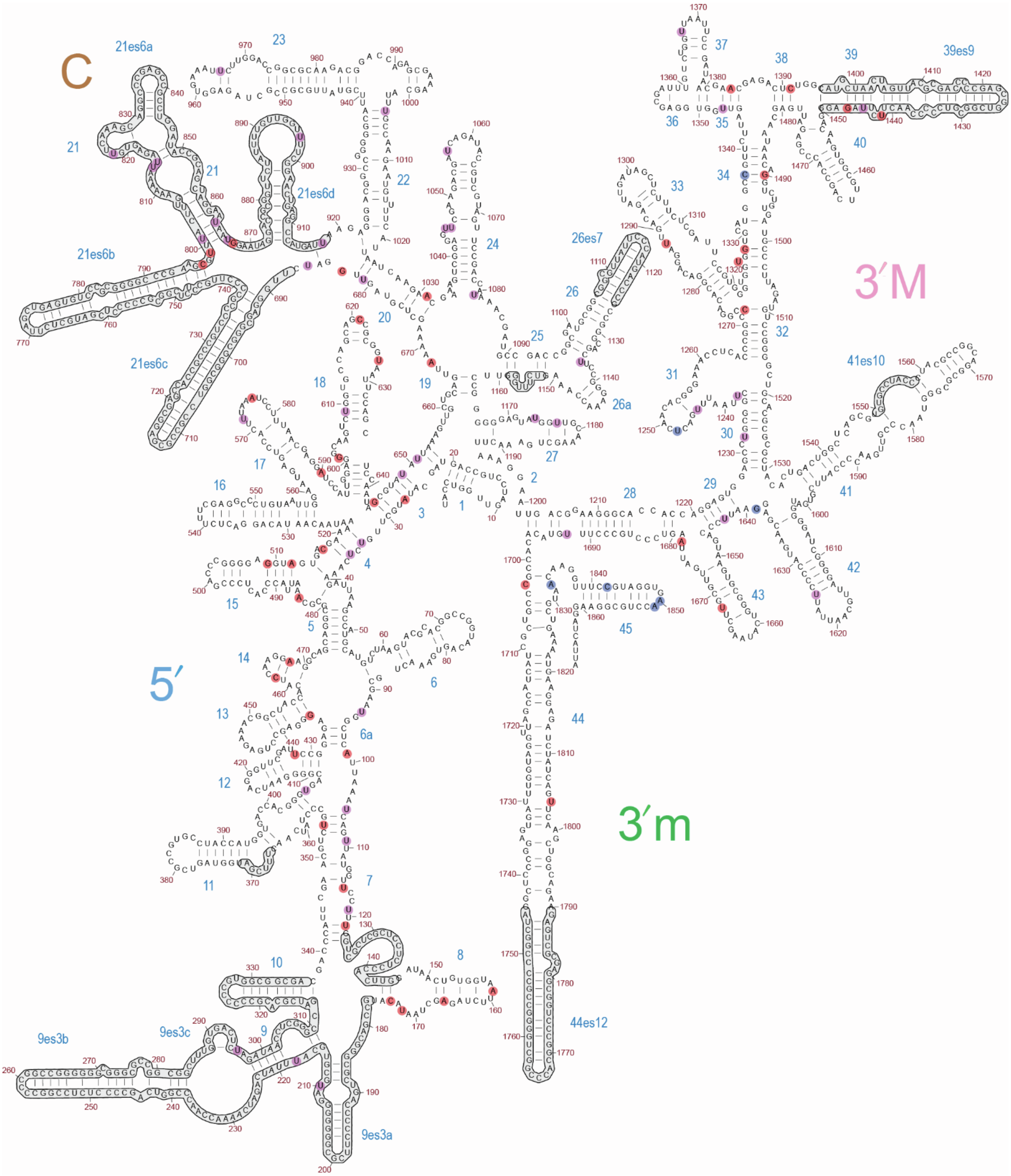

Supplementary Figure S3: ***S. lycopersicum*** **(top) and** ***H. sapiens*** **(bottom) structure-derived rRNA 2D diagrams for the 18S rRNA.** Base-pair interactions of canonical Watson-Crick pairs, standard G-U pairs and other local non-canonical ones are marked as dashes, black bullets and white bullets, respectively. Expansion segments are encircled and highlighted with grey. 2′-*O*-methylations are red, base modification blue, pseudouridines magenta, insertions yellow, point mutations green, and deletions are red line

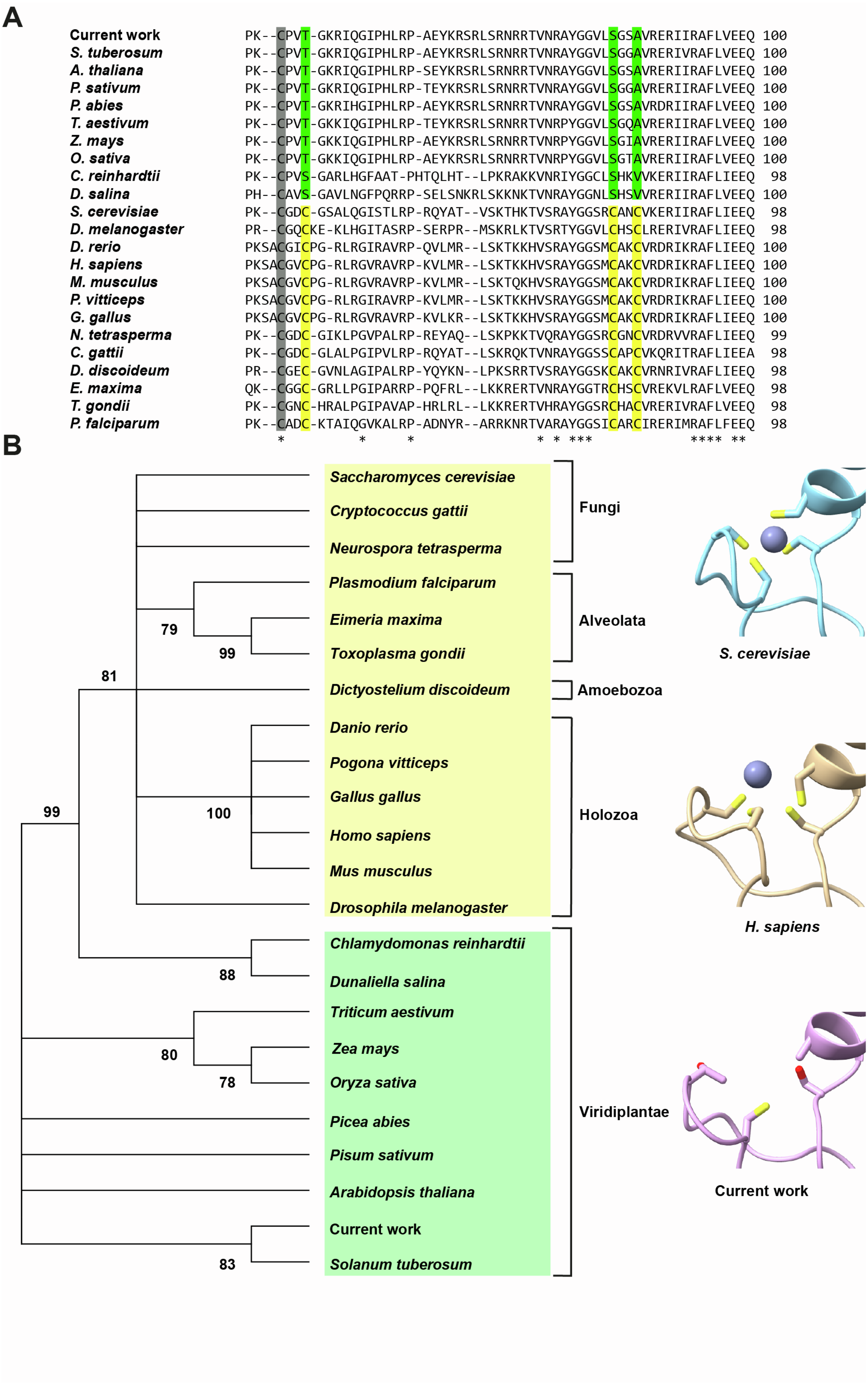
A
Current work PK--CPVT-GKRIQGIPHLRP-AEYKRSRLSRNRRTVNRAYGGVLSGSAVRERIIRAFLVEEQ 100
S. tuberosum PK--CPVT-GKRIQGIPHLRP-AEYKRSRLSRNRRTVNRAYGGVLSGGAVRERIVRAFLVEEQ 100
A. thaliana PK--CPVT-GKRIQGIPHLRP-SEYKRSRLSRNRRTVNRAYGGVLSGSAVRERIIRAFLVEEQ 100
P. sativum PK--CPVT-GKRIQGIPHLRP-TEYKRSRLSRNRRTVNRAYGGVLSGGAVRERIIRAFLVEEQ 100
P. abies PK--CPVT-GKRIHGIPHLRP-AEYKRSRLSRNRRTVNRAYGGVLSGSAVRDRIIRAFLVEEQ 100
T. aestivum PK--CPVT-GKKIQGIPHLRP-TEYKRSRLSRNRRTVNRPYGGVLSGQAVRERIIRAFLVEEQ 100
Z. mays PK--CPVT-GKKIQGIPHLRP-AEYKRSRLSRNRRTVNRPYGGVLSGIAVRERIIRAFLVEEQ 100
O. sativa PK--CPVT-GKKIQGIPHLRP-AEYKRSRLSRNRRTVNRPYGGVLSGTAVRERIIRAFLVEEQ 100
C. reinhardtii PK--CPVS-GARLHGFAAT-PHTQLHT--LPKRAKKVNRIYGGCLSHKVVKERIIRAFLIEEQ 98
D. salina PH--CAVS-GAVLNGFPQRRP-SELSNKRLSKKNKTVNRAYGGNLSHSVVRERIIRAFLVEEQ 100
S. cerevisiae PK--CGDC-GSALQGISTLRP-RQYAT--VSKTHKTVSRAYGGSRCANCVKERIIRAFLIEEQ 98
D. melanogaster PR--CGQCKE-KLHGITASRP-SERPR--MSKRLKTVSRTYGGVLCHSCLRERIVRAFLIEEQ 98
D. rerio PKSACGICPG-RLRGIRAVRP-QVLMR--LSKTKKHVSRAYGGSMCAKCVRDRIKRAFLIEEQ 100
H. sapiens PKSACGVCPG-RLRGVRAVRP-KVLMR--LSKTKKHVSRAYGGSMCAKCVRDRIKRAFLIEEQ 100
M. musculus PKSACGVCPG-RLRGVRAVRP-KVLMR--LSKTQKHVSRAYGGSMCAKCVRDRIKRAFLIEEQ 100
P. vitticeps PKSACGVCPG-RLRGIRAVRP-KVLMR--LSKTKKHVSRAYGGSMCAKCVRDRIKRAFLIEEQ 100
G. gallus PKSACGVCPG-RLRGVRAVRP-KVLKR--LSKTKKHVSRAYGGSMCAKCVRDRIKRAFLIEEQ 100
N. tetrasperma PK--CGDC-GIKLPGVPALRP-REYAQ--LSKPKKTVQRAYGGSRCGNCVRDRVVRAFLIEEQ 99
C. gattii PK--CGDC-GLALPGIPVLRP-RQYAT--LSKRQKTVNRAYGGSSCAPCVKQRITRAFLIEEA 98
D. discoideum PR--CGEC-GVNLAGIPALRP-YQYKN--LPKSRRTVSRAYGGSKCAKCVRNRIVRAFLIEEQ 98
E. maxima QK--CGGC-GRLLPGIPARRP-PQFRL--LKKRERTVNRAYGGTRCHSCVREKVLRAFLVEEQ 98
T. gondii PK--CGNC-HRALPGIPAVAP-HRLRL--LKKRERTVHRAYGGSRCHACVRERIVRAFLVEEQ 98
P. falciparum PK--CADC-KTAIQGVKALRP-ADNYR--ARRKNRTVARAYGGSICARCIRERIMRAFLFEEQ 98
* * * * * *** **** **
B
Saccharomyces cerevisiae
Cryptococcus gattii
Neurospora tetrasperma
Fungi
Plasmodium falciparum
Eimeria maxima
Toxoplasma gondii
Alveolata
79
99
81
Dictyostelium discoideum
Amoebozoa
Danio rerio
Pogona vitticeps
Gallus gallus
Homo sapiens
Mus musculus
Drosophila melanogaster
Holozoa
100
99
Chlamydomonas reinhardtii
Dunaliella salina
88
Triticum aestivum
Zea mays
Oryza sativa
80
78
Picea abies
Pisum sativum
Arabidopsis thaliana
Current work
Solanum tuberosum
83
Viridiplantae
S. cerevisiae
H. sapiens
Current work

Protein eL34 sequences used for the tree reconstruction:

**ORGANISM**	**SEQUENCE**
Solanum tuberosum	XP_006354783.1
Arabidopsis thaliana	NP_174010.1
Pisum sativum	P40590.1
Picea abies	MA_175574g0010
Clhamydomonas reinhardtii	XP_001697940.1
Dunaliella salina	KAF5843135.1
Saccharomyces cerevisiae	NP_010977.2
Drosophila melanogaster	AAL49199.1
Danio rerio	NP_957416.1
Homo sapiens	P49207
Mus musculus	NP_001005859.1
Pogona vitticeps	XP_020652019.1
Gallus gallus	NP_001268429.1
Cryptococcus gattii	XP_003191786.1
Dictyostelium discoideum	XP_637722.1
Eimeria máxima	XP_013335039.1
Toxoplasma gondii	XP_002366393.1
Plasmodium falciparum	XP_001349019.1

Supplementary Figure S4: Phylogenetic analysis of eL34 zinc finger. (a) Multiple sequence of the zinc finger region. The conserved cysteine is highlighted in gray, three complementary cysteines forming the zinc finger in yellow, and the corresponding *Viridiplantae* residues in green. (b) Maximum likelihood tree condensed at 70%. The clades with the conserved zinc finger are highlighted in yellow, *Viridiplantae* clade in green. Representative structures of the zinc finger or the corresponding part are shown on the right.

A

25S

```
database (S. lycopersicum)   UCCC-UGGAA 195
current work                 UCCCCUGGAA 196
A. thaliana                  UUCCCUGGAA
O. sativa                    UCCCCUGGAA
T. aestivum                  --C----GAC
Z. mays                      UCCCCUGGAA
                               *    **
```

```
database (S. lycopersicum)   G-AAAGGUUC-A 1499
current work                 GGAAAGGUUCCA 1509
A. thaliana                  GGAAAGGUUCCA
O. sativa                    AGAAAGGUUCCA
T. aestivum                  AGAAAGGUUCCA
Z. mays                      AGAAAGGUUCCA
                             * ******** *
```

```
database (S. lycopersicum)   ACGAGACGAA 2676
current work                 ACA----GAA 2683
A. thaliana                  ACA----GGA
O. sativa                    ACA----GGA
T. aestivum                  ACA----GGA
Z. mays                      ACA----GGA
                             **     ***
```

```
database (S. lycopersicum)   GGGG-CGCCG 204
current work                 GGGGGCGCCG 206
A. thaliana                  AGGGGCGCCA
O. sativa                    AGGGGCGCCU
T. aestivum                  AGGGGCGCCU
Z. mays                      AGGGACGCCU
                              *** ****
```

```
database (S. lycopersicum)   GGGG-AACCC 1550
current work                 GGGGGAACCC 1561
A. thaliana                  GGGGGAAACC
O. sativa                    GGGGUAACCC
T. aestivum                  GGGGUAACCC
Z. mays                      GGGGGAAACC
                             **** ** **
```

```
database (S. lycopersicum)   AAGCCUCCGU 2710
current work                 AAGC-U-CGU 2715
A. thaliana                  AAGC-U-CGU
O. sativa                    AAGC-U-CGU
T. aestivum                  AAGC-U-CGU
Z. mays                      AAGC-U-CGU
                             **** * ***
```

```
database (S. lycopersicum)   C-UGAGGG-U 747
current work                 CCUGAGGGGU 751
A. thaliana                  CUCGCGGG-U
O. sativa                    CUCACGGGCC
T. aestivum                  CUCACGGGCC
Z. mays                      CUCACGAGCC
                             *     * *
```

```
database (S. lycopersicum)   AAAUUCCGGA 1803
current work                 AAAU-CCGGA 1812
A. thaliana                  AAAU-CCGGA
O. sativa                    AAAU-CCGGA
T. aestivum                  AAAU-CCGGA
Z. mays                      AAAU-CCGGA
                             **** *****
```

```
database (S. lycopersicum)   UUCCGAGUAC 2731
current work                 UUCC-AGUAC 2735
A. thaliana                  UUUC-AGUAC
O. sativa                    UUCC-AGUAC
T. aestivum                  UUCC-AGUAC
Z. mays                      UUCC-AGUAC
                             ** * *****
```

```
database (S. lycopersicum)   CGGG-CGAAG 843
current work                 CGGGGCGAAG 848
A. thaliana                  CGGGGUAAAG
O. sativa                    CGGGGCGAAG
T. aestivum                  CGGGGCGAAG
Z. mays                      CGGGGCGAAG
                             ****    ***
```

```
database (S. lycopersicum)   CCGUUCCACG 1823
current work                 CCGU-CCACG 1831
A. thaliana                  CCGC-UCACG
O. sativa                    CCGU-CCACG
T. aestivum                  CCGU-UCACG
Z. mays                      CCAU-CCACG
                             **    ****
```

```
database (S. lycopersicum)   GCCUUAACGA 2763
current work                 GCCU-AACGA 2766
A. thaliana                  GCCU-AUCGA
O. sativa                    GCCU-AUCGA
T. aestivum                  GCCU-AUCGA
Z. mays                      GCCU-AUCGA
                             **** * ***
```

```
database (S. lycopersicum)   GG-UAUAGGG-C 914
current work                 GGGUAUAGGGGC 921
A. thaliana                  GGGUAUAGGGGC
O. sativa                    GGGUAUAGGGGC
T. aestivum                  GGGUAUAGGGGC
Z. mays                      GGGUAUAGGGGC
                             ** ******* *
```

```
database (S. lycopersicum)   GUGA-GAAAU 2219
current work                 GUGAAGAAAU 2228
A. thaliana                  GUGAAGAAAU
O. sativa                    GUGAAGAAAU
T. aestivum                  GUGAAGAAAU
Z. mays                      GUGAAGAAAU
                             **** *****
```

```
database (S. lycopersicum)   CCCGAAGGGC 3201
current work                 CCCCAAGGGC 3207
A. thaliana                  ----------
O. sativa                    CCCCAAGGGC
T. aestivum                  CCCCAAGGGC
Z. mays                      CCCCAAGGGC
```

```
database (S. lycopersicum)   UUCUUCAAAC 1054
current work                 UUCU-CAAAC 1061
A. thaliana                  UUCU-CAAAC
O. sativa                    UUCU-CAAAC
T. aestivum                  UUCU-CAAAC
Z. mays                      UUCU-CAAAC
                             **** *****
```

```
database (S. lycopersicum)   ACAU-GUCAG 2594
current work                 ACAUUGUCAG 2606
A. thaliana                  ACAUUGUCAG
O. sativa                    ACAUUGUCAG
T. aestivum                  ACAUUGUCAG
Z. mays                      ACAUUGUCAG
                             **** *****
```

```
database (S. lycopersicum)   G----GUC-GA 3240
current work                 GUCGCGGU-GA 3250
A. thaliana                  -----------
O. sativa                    GCCGCGGCCGG
T. aestivum                  GCCGCGGUCGG
Z. mays                      GCCGCGGCGGG
```

```
database (S. lycopersicum)   GCAA-UAUUC 1462
current work                 GCAAAUAUUC 1470
A. thaliana                  GCAAAUAUUC
O. sativa                    GCAAAUAUUC
T. aestivum                  GCAAAUAUUC
Z. mays                      GCAAAUAUUC
                             **** *****
```

```
database (S. lycopersicum)   CAACCGAGAA 2667
current work                 CAAC-GAGAA 2678
A. thaliana                  CAAC-GAGAA
O. sativa                    CAAC-GAGAA
T. aestivum                  CAAC-GAGAA
Z. mays                      CAAC-GAGAA
                             **** *****
```

18S

```
database (S. lycopersicum)   UGC----UGC 238
current work                 UGCCCGUUGC 242
A. thaliana                  UGCCCGUUGC
O. sativa                    CGCCCGCUGA
T. aestivum                  UGCUCGCUGA
Z. mays                      UGCCCGCCGA
                              **    *
```

```
database (S. lycopersicum)   CUA--GCCUU 1397
current work                 CUACGGCCUU 1403
A. thaliana                  CUAUGGCCGU
O. sativa                    CUAUGGCCGU
T. aestivum                  CUAUGGCCGU
Z. mays                      CUAUGGCCGU
                             ***   ***
```

```
database (S. lycopersicum)   GGCC-CACGC 1461
current work                 GGCCGCACGC 1468
A. thaliana                  GGCCGCACGC
O. sativa                    GGCCGCACGC
T. aestivum                  GGCCGCACGC
Z. mays                      GGCCGCACGC
                             **** *****
```

```
database (S. lycopersicum)   CUUG-CCGAC 1503
current work                 CUUGGCCGAC 1511
A. thaliana                  CUUGGCCGAC
O. sativa                    CUUGGCCGAC
T. aestivum                  CUUGGCCGAC
Z. mays                      CUUGGCCGAC
                             **** *****
```

5S

```
database (S. lycopersicum)   UUGCAUUCCC 120
current work                 UUGCAUCCCU 120
A. thaliana                  UUGCAUCCCC
O. sativa                    UUGCAUCCCC
T. aestivum                  UUGCAUCCC-
Z. mays                      UUGCAUUCCC
                             ****** **
```

B

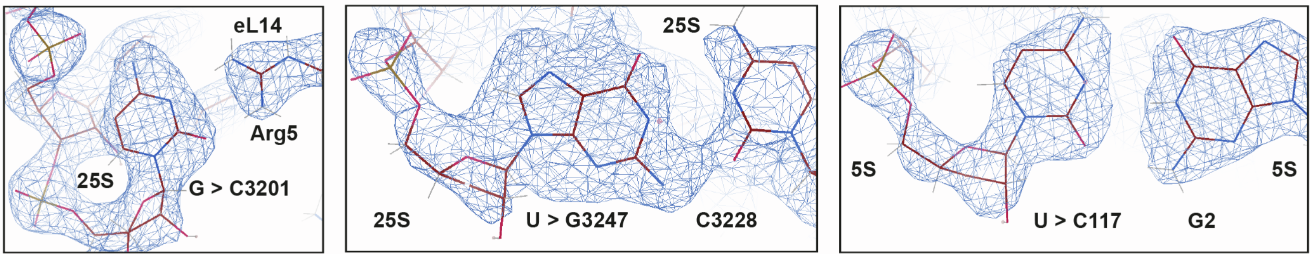

ORGANISM	SEQUENCE			
	**25S**	**18S**	**5.8S**	**5S**
Database (*S. lycopersicum*)	X13557.1	X51576.1	MH573911.1	X06842.1
Arabidopsis thaliana	LR797810.1 (3150-6000)	LR797808.1 (8000-10000)	LR782543.1 (5680-5860)	LR782545.1 (3150170-3150310)
Oryza sativa	AP014965.1 (29700-33200)	LC086814.1 (1-3000)	XR_003238527.1 (890-1070)	AP014967.1 (12099160-12099300)
Triticum aestivum	3J62_a	3J5Z_d	M10469.1	KY439627.1 (298-417)
Zea mays	NR_028022.2	AF168884.1	MF780726.1 (150-320)	DQ351339.1 (186-315)

rRNA sequences used for the multiple sequence alignments:

Supplementary Figure S5: rRNA sequence discrepancies with the available database. (a) Multiple sequence alignment, showing reannotations (green), deletions (red), insertions (yellow). The density and modifications have been assessed in accordance with previous studies from the lab at a similar resolution level (Itoh et al., 2022a; Itoh et al., 2022b). (b) Examples of point mutations identified from the density in 60S.

>25S
GCGACCCCAGGUCAGGCGGGAUUACCCGCUGAGUUUAAGCAUAUCAAUAAGC
GGAGGAAAAGAAACUUACAAGGAUUCCCCUAGUAACGGCGAGCGAACCGGGA
ACAGCCCAGCCUUAGAAUCGGGCGGCUCCGUCGUCCGAAUUGUAGUCUGGAG
AAGCGUCCUCAGCGGCGGACCGGGCCCAAGUCCCCUGGAAGGGGGCGCCGGA
GAGGGUGAGAGCCCCGUCGUGCCCGGACCCUGUCGCACCACGAGGCGCUGUC
UACGAGUCGGGUUGUUUGGGAAUGCAGCCCAAAUCGGGCGGUGAAUUCCGUC
CAAGGCUAAAUACUGGCGAGAGACCGAUAGCGAACAAGUACCGCGAGGGAAA
GAUGAAAAGGACUUUGAAAAGAGAGUCAAAGAGUGCUUGAAAUUGUCGGGAG
GGAAGCGGAUGGGGGCCGGCGAUGCGCCCCGGUCGGAUGUGGAACGGCGACG
AGCCGGUCCGCCGAUCGACUCGGGGCGUGGACCAGCGUGGAUUGGGGGGGCG
GCCAAAGCCCGGGCUCUCGAUACGCCCGUGGAACGCCGUCUCCCCGAUUGUG
GAAGGCAGCGCGCGCCUCCGGCGUGCUUCGGCAUCUGCGCGCUCCGGACGCU
GGCCUGUGGGCUCCCCAUUCGACCCGUCUUGAAACACGGACCAAGGAGUCUG
ACAUGUGUGCGAGUCAACGGGCGAGUAAACCCGUAAGGCGUAAGGAAGCUGA
UUGGUGGGAUCCCCCUGAGGGGUGCACCGCCGACCGACCUUGAUCUUCUGAG
AAGGGUUCGAGUGUGAGCAUACCUGUCGGGACCCGAAAGAUGGUGAACUAUG
CCUGAGCGGGGCGAAGCCAGAGGAAACUCUGGUGGAGGCCCGCAGCGAUACU
GACGUGCAAAUCGUUCGUCUGACUUGGGUAUAGGGGCGAAAGACUAAUCGAA
CCGUCUAGUAGCUGGUUCCCUCCGAAGUUUCCCUCAGGAUAGCUGGAGCUCG
CGUGCGAGUUCUAUCGGGUAAAGCCAAUGAUUAGAGGCCUCGGGGGCGCAAC
GCCCUCGACCUAUUCUCAAACUUUAAAUAGGUAGGACGGCGCGGCUGCUUUG
UUGAGCCGCGCCACGGAAUCAAGAGCUCCAAGUGGGCCAUUUUUGGUAAGCA
GAACUGGCGAUGCGGGAUGAACCGGAAGCCGGGUUACGGUGCCAAACUGCGC
GCUAACCUAGAUCCCACAAAGGGUGUUGGUCGAUUAAGACAGCAGGACGGUG
GUCAUGGAAGUCGAAAUCCGCUAAGGAGUGUGUAACAACUCACCUGCCGAAU
CAACUAGCCCCGAAAAUGGAUGGCGCUUAAGCGCGCGACCUACACCCGGCCG
UCGGGGCAAGUGCCAGGCCCCGAUGAGUAGGAGGGCGCGGCGGUCGCUGCAA
AACCUUGGGCGCGAGCCUGGGCGGAGCGGCCGUCGGUGCAGAUCUUGGUGGU
AGUAGCAAAUAUUCAAAUGAGAACUUUGAAGGCCGAAGAGGGGAAAGGUUCC
AUGUGAACGGCACUUGCACAUGGGUUAGUCGAUCCUAAGGGUCGGGGGAACC
CCGACAGAUAGCGCGUUUCGCGCGUACUCCGAAAGGGAAUCGGGUUAAAAUU
CCUGAACCGGGACGUGGCGGUUGACGGCAACGUUAGGAAGUCCGGAGACGUC
GGCGGGAGCCUCGGGAAGAGUUAUCUUUUCUGUUUAACAGCCUGCCCACCCU
GGAAUCGGCUCAGCCGGAGGUAGGGUCCAGCGGCUGGAAGAGCACCGCACGU
CGCGUGGUGUCCGGUGCGCUCCCGGCGGCCCUUGAAAAUCCGGAGGACCGAA
UGCCGUCCACGCCCGGUCGUACUCAUAACCGCAUCAGGUCUCCAAGGUGAAC
AGCCUCUGGUCGAUGGAACAAUGUAGGCAAGGGAAGUCGGCAAAAUGGAUCC
GUAACUUCGGGAAAAGGAUUGGCUCUGAGGGCUGGGCACGGGGGUCCCAGUC
CCGAACCCGUCGGCUGUCGGUGGACUGCUCGAGCUGCUCCCGCGGCGAGAGC
GGGUCGCCGCGUGCCGGCCGGGGGACGGACUGGGAACGGUUCCUUCGGGGGC
CUUCCCCGGGCGUCGAACAGCCAACUCAGAACUGGUACGGACAAGGGGAAUC
CGACUGUUUAAUUAAAACAAAGCAUUGCGAUGGUCCCAACGGAUGUUUACGC
AAUGUGAUUUCUGCCCAGUGCUCUGAAUGUCAAAGUGAAGAAAUUCAACCAA
GCGCGGGUAAACGGCGGGAGUAACUAUGACUCUCUUAAGGUAGCCAAAUGCC
UCGUCAUCUAAUUAGUGACGCGCAUGAAUGGAUUAACGAGAUUCCCACUGUC
CCUGUCUACUAUCCAGCGAAACCACAGCCAAGGGAACGGGCUUGGCAGAAUC
AGCGGGGAAAGAAGACCCUGUUGAGCUUGACUCUAGUCCGACUUUGUGAAAU
GACUUGAGAGGUGUAGUAUAAGUGGGAGCCGAAAGGCGAAAGUGAAAUACCA
CUACUUUUAACGUUAUUUUACUUAUUCCGUGAAUCGGAAGCGGGGCACUGCC
CCUCUUUUUGGACCCAAGGCUCGCUUCGCGGGCCGAUCCGGGCGGAAGACAU
UGUCAGGUGGGGAGUUUGGCUGGGGCGGCACAUCUGUUAAAAGAUAACGCAG
GUGUCCUAAGAUGAGCUCAACGAGAACAGAAAUCUCGUGUGGAACAGAAGGG
UAAAAGCUCGUUUGAUUCUGAUUUCCAGUACGAAUACGAACCGUGAAAGCGU
GGCCUAACGAUCCUUUAGACCUUCGGAAUUCGAAGCUAGAGGUGUCAGAAAA
GUUACCACAGGGAUAACUGGCUUGUGGCAGCCAAGCGUUCAUAGCGACGUUG
CUUUUUGAUCCUUCGAUGUCGGCUCUUCCUAUCAUUGUGAAGCAGAAUUCAC
CAAGUGUUGGAUUGUUCACCCACCAAUAGGGAACGUGAGCUGGGUUUAGACC
GUCGUGAGACAGGUUAGUUUUACCCUACUGAUGACAGUGUCGCAAUAGUAAU
UCAACCUAGUACGAGAGGAACCGUUGAUUCACACAAUUGGCCAUCGCGCUUG
GUUGAAAAGCCAGUGGCGCGAAGCUACCGUGUGCUGGAUUAUGACUGAACGC
CUCUAAGUCAGAAUCCGGGCUAGAAGCGACGCAUGCGCCCGCCGUCCGCUUG
CCGACCCGCAGUAGGGGCCUUUGGCCCCCAAGGGCACGUGUCGUUGGCUAAG
UCGCCGCGACGGAAGCGUCGCGGUGACCGCCUUGAAGUACAAUUUCCAUCGA
GCGGCGGGUAGAAUCCUUUGCAGACGACUUAAAUACGCGACGGGGUAUUGUA
AGUGGCAGAGUGGCCUUGCUGCCACGAUCCACUGAGAUUCAGCCCUUUGUCG
CUCCGAUUCGU
>18
UACCUGGUUGAUCCUGCCAGUAGUCAUAUGCUUGUCUCAAAGAUUAAGCCAU
GCAUGUGUAAGUAUGAACAAAUUCAGACUGUGAAACUGCGAAUGGCUCAUUA
AAUCAGUUAUAGUUUGUUUGAUGGUAUCUACUACUCGGAUAACCGUAGUAAU
UCUAGAGCUAAUACGUGCAACAAACCCCGACUUCUGGAAGGGAUGCAUUUAU
UAGAUAAAAGGUCGACGCGGGCUCUGCCCGUUGCUGCGAUGAUUCAUGAUAA
CUCGACGGAUCGCACGGCCAUCGUGCCGGCGACGCAUCAUUCAAAUUUCUGC
CCUAUCAACUUUCGAUGGUAGGAUAGUGGCCUACCAUGGUGGUGACGGGUGA
CGGAGAAUUAGGGUUCGAUUCCGGAGAGGGAGCCUGAGAAACGGCUACCACA
UCCAAGGAAGGCAGCAGGCGCGCAAAUUACCCAAUCCUGACACGGGGAGGUA
GUGACAAUAAAUAACAAUACCGGGCUCUAUGAGUCUGGUAAUUGGAAUGAGU
ACAAUCUAAAUCCCUUAACGAGGAUCCAUUGGAGGGCAAGUCUGGUGCCAGC
AGCCGCGGUAAUUCCAGCUCCAAUAGCGUAUAUUUAAGUUGUUGCAGUUAAA
AAGCUCGUAGUUGGACUUUGGGAUGGGCCGGCCGGUCCGCCCUAGGUGUGCA
CCGGUCGUCUCGUCCCUUCUGUCGGCGAUGCGCUCCUGGCCUUAAUUGGCCG
GGUCGUGCCUCCGGCGCUGUUACUUUGAAGAAAUUAGAGUGCUCAAAGCAAG
CCUACGCUCUGUAUACAUUAGCAUGGGAUAACAUUAUAGGAUUUCGGUCCUA
UUACGUUGGCCUUCGGGAUCGGAGUAAUGAUUAACAGGGACAGUCGGGGGCA
UUCGUAUUUCAUAGUCAGAGGUGAAAUUCUUGGAUUUAUGAAAGACGAACAA
CUGCGAAAGCAUUUGCCAAGGAUGUUUUCAUUAAUCAAGAACGAAAGUUGGG
GGCUCGAAGACGAUCAGAUACCGUCCUAGUCUCAACCAUAAACGAUGCCGAC
CAGGGAUCGGCGGAUGUUGCUUUUAGGACUCCGCCGGCACCUUAUGAGAAAU
CAAAGUUUUUGGGUUCCGGGGGGAGUAUGGUCGCAAGGCUGAAACUUAAAGG
AAUUGACGGAAGGGCACCACCAGGAGUGGAGCCUGCGGCUUAAUUUGACUCA
ACACGGGGAAACUUACCAGGUCCAGACAUAGUAAGGAUUGACAGACUGAGAG
CUCUUUCUUGAUUCUAUGGGUGGUGGUGCAUGGCCGUUCUUAGUUGGUGGAG
CGAUUUGUCUGGUUAAUUCCGUUAACGAACGAGACCUCAGCCUGCUAACUAG
CUAUGCGGAGGUAUCCCUUCGCGGCCAGCUUCUUAGAGGGACUACGGCCUUU
UAGGCCGCGGAAGUUUGAGGCAAUAACAGGUCUGUGAUGCCCUUAGAUGUUC
UGGGCCGCACGCGCGCUACACUGAUGUAUUCAACGAGCUUAUAGCCUUGGCC
GACAGGCCCGGGUAAUCUUUGAAAUUUCAUCGUGAUGGGGAUAGAUCAUUGC
AAUUGUUGGUCUUCAACGAGGAAUUCCUAGUAAGCGCGAGUCAUCAGCUCGC
GUUGACUACGUCCCUGCCCUUUGUACACACCGCCCGUCGCUCCUACCGAUUG
AAUGAUCCGGUGAAAUGUUCGGAUCGCGGCGACGUGGGCGGUUCGCUGCCCG
CGACGUCGCGAGAAGUCCAUUGAACCUUAUCAUUUAGAGGAAGGAGAAGUCG
UAACAAGGUUUCCGUAGGUGAACCUGCGGAAGGAUCAUUG
>5.8S
ACAAACGACUCUCGGCAACGGAUAUCUCGGCUCUCGCAUCGAUGAAGAACGU
AGCGAAAUGCGAUACUUGGUGUGAAUUGCAGAAUCCCGUGAACCAUCGAGUC
UUUGAACGCAAGUUGCGCCCGAAGCCAUUUGGCCGAGGGCACGUCUGCCUGG
GCGUCACGC
>5S
GGAUGCGAUCAUACCAGCACUAACGCACCGGAUCCCAUCAGAACUCCGAA
GUUAAGCGUGCUUGGGCGAGAGUAGUACUAGGAUGGGUGACCCCCUGGGA
AGUCCUCGUGUUGCAUCCCU

Supplementary Figure S6: Corrected *S. lycopersicum* rRNA sequences.

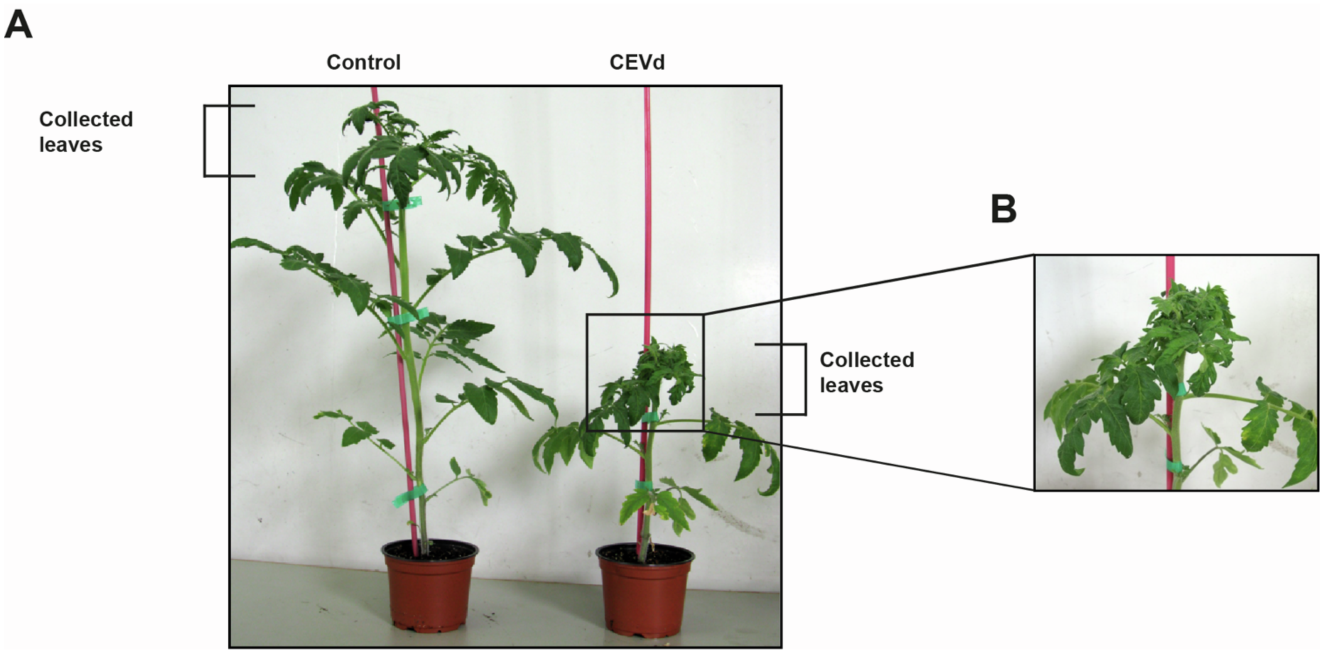

Supplementary Figure S7: Control and stressed plants used for the experiments three weeks post-inoculation (a) To stimulate stress conditions, plants were CEVd (Citrus exocortis viroid) infected by puncturing. (b) Zoom in to the plant's apex that was used to collect leaves for the experiment.

Supplementary Table S1: Cryo-EM data collection, processing and model refinement statistics.

	60S	40S body	40S head	Combined
**Data collection and processing**				
Microscope	Titan Krios	Titan Krios	Titan Krios	Titan Krios
Detector	K2 Summit	K2 Summit	K2 Summit	K2 Summit
Magnification	165,000	165,000	165,000	165,000
Voltage [kV]	300	300	300	300
Total electron exposure [$e^{-}/Å^2$]	29–32	29–32	29–32	29–32
Defocus range [μm]	−1.0 to −2.8	−1.0 to −2.8	−1.0 to −2.8	−1.0 to −2.8
Pixel size [Å]	0.82–0.83	0.82–0.83	0.82–0.83	0.82–0.83
Symmetry imposed	C_1	C_1	C_1	C_1
Final particle	335,806	335,806	335,806	335,806
Resolution [Å]	2.35	2.53	2.58	2.38
Map-sharpening *B* factor [$Å^2$]	−44.17	−44.17	−43.47	−47.28
**Refinement**				
Model composition				
Total atoms (non-hydrogen / hydrogen)	127,702 / 89,168	47,741 / 36,377	24,563 / 19587	199,939 / 145,068
Chains (RNA/ protein)	4 / 41	1 / 21	3 / 13	7 / 73
RNA residues (non-modified / modified)	3,252 / 127	1060 / 50	464 / 27	4,776 / 204
Protein residues (non-modified / modified)	6,329 / 2	2,911 / 1	1,730 / 0	10,963 / 3
Metal ions (Mg^{2+}/ K^{+}/ Zn^{2+})	276 / 90 / 4	60 / 19 / 1	28 / 10 / 1	364 / 119 / 6
Ligands (SPD / SPM / PUT/ sugar)	1 / 3 / 0 / 0	0 / 0 / 0 / 0	0 / 0 / 1 / 1	1 / 3 / 1 / 1
Waters	3,939	570	225	4,734
Model to map CC (CC_{mask} / CC_{box} / CC_{peaks} / CC_{volume})	0.83 / 0.74 / 0.69 / 0.80	0.86 / 0.74 / 0.69 / 0.82	0.89 / 0.79 / 0.75 / 0.85	0.83 / 0.78 / 0.75 / 0.80
Resolution [Å] by model-to-map FSC, threshold 0.50 (masked/ unmasked)	2.23 / 2.24	2.38 / 2.38	2.34 / 2.43	2.31 / 2.31
Average *B* factor [$Å^2$] (RNA / protein / metal ion and ligand / water)	19 / 13 / 8.2 / 4.9	23 / 25 / 13 / 11	25 / 35 / 20 / 15	21 / 18 / 12 / 8.1
R.m.s. deviations				
Bond lengths [Å]	0.002	0.002	0.003	0.002
Bond angles [°]	0.412	0.441	0.489	0.387
**Validation**				
Clash score	1.86	1.94	1.89	2.02
Rotamer outliers [%]	0.07	0.08	0.00	0.06
Ramachandran plot [%] (Favored / allowed/ disallowed)	98.70 / 1.29 / 0.02	99.13 / 0.87 / 0.00	98.47 / 1.53 / 0.00	99.04 / 0.95 / 0.01
CaBLAM outliers [%]	1.06	0.82	1.20	0.96
MolProbity score	0.95	0.96	0.95	0.97
**Accession ID**				
EMDB ID	EMD-14001	EMD-14002	EMD-14003	EMD-14004
PDB ID	7QIW	7QIX	7QIY	7QIZ

Supplementary Table S2: Re-annotations of the rRNAs in 60S, composed of deletions, insertions and point mutations observed according to the map.

rRNA	position	residue/s	re-annotation
**25S**			
H38a	1056-1057	U	deletion
H56	1807-1808	U	deletion
H54	1826-1827	U	deletion
H84	2673-2674	C	deletion
H84	2680-2681	AGAC	deletion
H83	2711-2712	C	deletion
H83	2712-2713	C	deletion
H86	2730-2731	G	deletion
H82	2761-2762	U	deletion
H19	191	C	insertion
H19	201	G	insertion
ES9	743	C	insertion
ES9	750	G	insertion
ES7a	843	G	insertion
H33	912	G	insertion
H33	920	G	insertion
H47	1465	A	insertion
H49	1499	G	insertion
H50	1508	C	insertion
H52	1556	G	insertion
H66	2223	A	insertion
H75	2601	U	insertion
ES39b	3242-3245	UCGU	insertion
ES39a	3201	G → C	point mutation
ES39a	3247	U → G	point mutation
**5S**			
	117	U → C	point mutation
	120	C → U	point mutation

Supplementary Table S3: Re-annotations of the rRNA in 40S, composed of insertions and point mutations observed according to the map.

rRNA	position	residue/s	re-annotation
**18S**			
es3b	236-239	CCGU	insertion
h40	1397-1398	CG	insertion
h30	1463	G	insertion
es10	1506	G	insertion

Supplementary Table S4: Plant specific rRNA modifications in 60S.

rRNA	residue	modification	rRNA	residue	modification
**25S**			**25S**		
H11	U35	Pseudouridine	H90	U2887	2′-*O*-methylation
H11	U48	2′-*O*-methylation	H91	A2915	2′-*O*-methylation
H12	U68	Pseudouridine	H73	U2996	Pseudouridine
H15	U144	2′-*O*-methylation	H100	U3305	2′-*O*-methylation
H3	A369	2′-*O*-methylation			
H24	U378	Pseudouridine			
H3	G399	2′-*O*-methylation			
H27	U675	2′-*O*-methylation			
H27	U804	2′-*O*-methylation			
H32	A946	2′-*O*-methylation			
H38a	U1068	2′-*O*-methylation			
H39	U1134	Pseudouridine			
H42	U1230	Pseudouridine			
H43	U1250	Pseudouridine			
H43	A1252	2′-*O*-methylation			
H43	A1264	2′-*O*-methylation			
H44	U1279	2′-*O*-methylation			
H44	U1288	Pseudouridine			
H45	A1378	2′-*O*-methylation			
H47	U1474	Pseudouridine			
H47	C1480	2′-*O*-methylation			
H47	U1482	Pseudouridine			
H51	U1537	2′-*O*-methylation			
H49a	C1849	2′-*O*-methylation			
H49a	C1852	2′-*O*-methylation			
H49a	G1857	2′-*O*-methylation			
H63	U2116	2′-*O*-methylation			
H64	A2129	2′-*O*-methylation			
H68	U2228	Pseudouridine			
H68	G2239	2′-*O*-methylation			
H71	C2296	2′-*O*-methylation			
H75	U2435	Pseudouridine			
H76	U2449	Pseudouridine			
H76	U2458	2′-*O*-methylation			
H78	G2486	2′-*O*-methylation			
H76	U2504	Pseudouridine			
H79	U2521	Pseudouridine			
H84	C2686	2′-*O*-methylation			
H83	U2716	Pseudouridine			
H86	U2721	2′-*O*-methylation			
H86	U2739	2′-*O*-methylation			
H89	C2840	2′-*O*-methylation			
**5.8S**					
H5	A48	2′-*O*-methylation			
H7	U79	Pseudouridine			

Supplementary Table S5: Plant specific rRNA modifications in 40S.

**rRNA**	**residue**	**modification**
**18S**		
h7	U103	Pseudouridine
h8	C140	2′-*O*-methylation
es3a	U188	Pseudouridine
es3b	G246	2′-*O*-methylation
h9	U255	Pseudouridine
h9	U258	Pseudouridine
h12	U383	Pseudouridine
h18	U584	Pseudouridine
h3	U603	2′-*O*-methylpseudouridine
h19	U614	2′-*O*-methylation
h21	U753	Pseudouridine
h22	U949	Pseudouridine
h24	U1012	2′-*O*-methylation
h25	U1106	Pseudouridine
h32	U1210	Pseudouridine
h33	U1263	2′-*O*-methylation
h33	U1265	2′-*O*-methylation
h34	U1447	2′-*O*-methylation
h41	U1485	Pseudouridine
h41	U1535	Pseudouridine
h41	U1538	Pseudouridine
h42	A1579	2′-*O*-methylation
h44	A1758	2′-*O*-methylation

Supplementary Table S6: Conserved rRNA modifications in 60S.

rRNA	residue	modification	rRNA	residue	modification
**25S**			**25S**		
H11	U44	2′-*O*-methylation	H64	A2324	2′-*O*-methylation
H25a	A656	N1-methylation	H64	A2329	2′-*O*-methylation
H25a	A660	2′-*O*-methylation	H61	C2340	2′-*O*-methylation
H27	C674	2′-*O*-methylation	H61	U2350	2′-*O*-methylation
H32	G815	2′-*O*-methylation	H61	U2352	Pseudouridine
H32	A817	2′-*O*-methylation	H72	C2368	2′-*O*-methylation
H33	A827	2′-*O*-methylation	H73	G2394	2′-*O*-methylation
H33	U829	Pseudouridine	H73	G2398	2′-*O*-methylation
H35	A886	2′-*O*-methylation	H74	G2412	2′-*O*-methylation
H35	U895	Pseudouridine	H74	U2413	2′-*O*-methylation
H35a	G918	2′-*O*-methylation	H74	U2419	Pseudouridine
H37	U970	Pseudouridine	H75	U2424	2′-*O*-methylation
H38a	U1002	Pseudouridine	H77	C2497	2′-*O*-methylation
H38a	U1016	Pseudouridine	H80	U2617	Pseudouridine
H38a	U1054	Pseudouridine	H80	G2623	2′-*O*-methylation
H38a	U1064	Pseudouridine	H81	A2644	2′-*O*-methylation
H39	U1133	Pseudouridine	H81	U2654	2′-*O*-methylation
H39	U1135	Pseudouridine	H81	G2655	2′-*O*-methylation
H39	A1144	2′-*O*-methylation	H86	U2733	2′-*O*-methylation
H26	C1448	2′-*O*-methylation	H87	U2748	Pseudouridine
H26a	A1460	2′-*O*-methylation	H88	G2795	2′-*O*-methylation
H26a	G1461	2′-*O*-methylation	H88	G2797	2′-*O*-methylation
H49	C1862	2′-*O*-methylation	H74	G2819	2′-*O*-methylation
H61	U1894	2′-*O*-methylation	H89	U2830	Pseudouridine
H61	U1909	Pseudouridine	H89	U2847	Pseudouridine
H64	G2126	2′-*O*-methylation	H89	U2858	Pseudouridine
H64	G2127	2′-*O*-methylation	H89	U2869	Pseudouridine
H65	U2137	Pseudouridine	H90	C2874	C^5-methylation
H65	U2139	Pseudouridine	H90	C2883	2′-*O*-methylation
H67	U2194	Pseudouridine	H90	U2884	Pseudouridine
H68	C2200	2′-*O*-methylation	H91	U2898	Pseudouridine
H68	U2214	Pseudouridine	H92	G2921	2′-*O*-methylation
H68	A2223	2′-*O*-methylation	H92	U2925	2′-*O*-methylation
H69	U2257	Pseudouridine	H92	G2926	2′-*O*-methylation
H69	A2259	2′-*O*-methylation	H92	U2927	Pseudouridine
H69	U2261	Pseudouridine	H90	U2948	Pseudouridine
H69	U2263	Pseudouridine	H90	A2950	2′-*O*-methylation
H69	U2267	Pseudouridine	H90	C2952	2′-*O*-methylation
H69	U2269	Pseudouridine	H90	U2957	N^3-methylation
H70	C2281	C^5-methylation	H93	U2959	Pseudouridine
H70	A2284	2′-*O*-methylation	H93	C2963	2′-*O*-methylation
H71	G2291	2′-*O*-methylation	H93	U2979	Pseudouridine
H67	U2317	Pseudouridine	H97	U3114	Pseudouridine
H67	U2321	Pseudouridine			
**5.8S**					
H3	U23	Pseudouridine			
H7	G80	2′-*O*-methylation			

Supplementary Table S7: Conserved rRNA modifications in 40S.

rRNA	residue	modification
**18S**		
h4	A28	2′-*O*-methylation
h5	C38	2′-*O*-methylation
h7	U111	Pseudouridine
h7	U121	Pseudouridine
h7	U123	2′-*O*-methylation
h8	A162	2′-*O*-methylation
h9	U208	Pseudouridine
h7	U300	Pseudouridine
h7	U306	Pseudouridine
h11	U362	Pseudouridine
h13	G392	2′-*O*-methylation
h14	C418	2′-*O*-methylation
h15	A440	2′-*O*-methylation
h15	U451	Pseudouridine
h15	A468	2′-*O*-methylation
h5	C473	2′-*O*-methylation
h17	A544	2′-*O*-methylation
h18	U581	2′-*O*-methylation
h4	G598	2′-*O*-methylation
h3	U605	Pseudouridine
h19	A622	2′-*O*-methylation
h20	U635	Pseudouridine
h21	U762	Pseudouridine
h21	A800	2′-*O*-methylation
h21	U809	Pseudouridine
h23	U912	Pseudouridine
h22	U950	Pseudouridine
h20	A977	2′-*O*-methylation
h24	U1002	Pseudouridine
h24	U1027	Pseudouridine
h27	U1120	Pseudouridine
h30	U1178	Pseudouridine
h30	U1184	Pseudouridine
h31	U1190	Pseudouridine
h31	U1194	1-methyl-3-(3-amino-3-carboxypropyl)pseudouridine
h32	U1217	Pseudouridine
h32	C1218	2′-*O*-methylation
h33	U1234	2′-*O*-methylation
h34	U1272	2′-*O*-methylation
h34	G1274	2′-*O*-methylation
h34	C1283	N^4-acetylation
h35	U1293	Pseudouridine
h36	U1304	Pseudouridine
h37	U1308	Pseudouridine
h37	U1313	Pseudouridine
h37	A1329	2′-*O*-methylation
**18S**		
h39	U1383	2′-*O*-methylation
h34	G1433	2′-*O*-methylation
H42	U1567	Pseudouridine
h42	G1581	N^7-methylation
h28	U1634	Pseudouridine
h44	C1645	2′-*O*-methylation
h44	A1771	N^6-methylation
h45	C1781	N^4-acetylation
h45	A1789	N^6,N^6-dimethylation
h45	A1790	N^6,N^6-dimethylation

Supplementary Table S9: Oligonucleotides.

Oligonucleotide	Sequence[a]
Tmt18S-270-chimera	AmCmGmAmUmGmGmCmCmGm(TGCG)AmUmCmCmGmUmCmGm
c18S-385-chimera	GmGmCmUmCmCmCmUmCm(TCCG)GmAmAmUmCmGmAmAmCmCmCm
c18S-580-chimera	AmGmCmUmGmGmAmAm(TTAC)CmGmCmGmGmCmUmGmCmUmGmGm
Tmt18S-831-chimera	CmCmGmAmAmGmGmCmCmAmAmCmGm(TAAT)AmGmGmAmCmCmGmAm
h18S-1210-chimera	CmCmUmGmGmUmGmGmUmGmCmCm(CTTC)CmGmUmCmAmAmUm
Tmt18S-1332-chimera	AmGmCmAmGmGmCmUmGmAm(GGTC)UmCmGmUmUmCmGmUmUmAm
Tmt18S-1597-chimera	AmGmCmUmGmAmUmGmAmCmUmCm(GCGC)UmUmAmCmUmAmGmGmAm
Tmt18S-1679-chimera	CmGmCmCmGmCmGmAmUmCmCmGm(AACA)UmUmUmCmAmCmCmGm
Tmt25S-418-chimera	CmCmCmCmAmUmCmCmGmCmUmUm(CCCT)CmCmCmGmAmCmAmAm
Tmt25S-643-chimera	UmCmAmAmGmAmCmGmGmGmUmCmGm(AATG)GmGmGmAmGmCmCm
Tmt25S-850-chimera	AmGmAmGmUmUmUmCmCmUmCmUm(GGCT)UmCmGmCmCmCmCmGm
h28S-1866-chimera	CmGmCmCmAmGmUmUmCmUmGmCmUmUm(ACCA)AmAmAmGmUmGmGmCmCm
Tmt25S-1521-chimera	AmCmCmCmAmUmGmUmGmCmAm(AGTG)CmCmGmUmUmCmAmCmAmUm
Tmt25S-1859-chimera	UmCmAmCmCmUmUmGmGmAmGm(ACCT)GmAmUmGmCmGmGmUmUm
Tmt25S-2119-chimera	CmCmCmCmUmUmGmUmCm(CGTA)CmCmAmGmUmUmCmUmGmAmGmUm
Tmt25S-2232-chimera	AmCmCmCmGmCmGmCmUmUmGmGm(TTGA)AmUmUmUmCmUmUmCmAm
h28S-3892-chimera	CmUmUmUmCmCmCmCmGmCmUmGm(ATTC)CmGmCmCmAmAmGmCmCm
Tmt25S-2611-chimera	GmCmCmCmCmAmGmCmCmAmAmAmCm(TCCC)CmAmCmCmUmGmAm
Tmt25S-2703-chimera	CmGmAmGmCmUmUmUmUmAmCmCm(CTTC)UmGmUmUmCmCmAmCmAm
Tmt25S-2755-chimera	CmGmUmUmAmGmGmCmCmAmCmGm(CTTT)CmAmCmGmGmUmUm
h28S-4443-chimera	GmCmCmGmAmCmAmUmCmGmAmAmGm(GATC)AmAmAmAmAmGmCmGmAmCmGmUm
h28S-4516-chimera	AmCmCmCmAmGmCmUmCmAmCmGmUmUm(CCCT)AmUmUmAmGmUmGmGmGmUm
p25S-3044-chimera	CmCmUmGmUmCmUmCmAmCm(GACG)GmUmCmUmAmAmAmCmCmCmAm
h28S-4563-chimera	CmAmUmCmAmUmCmAmGmUm(AGGG)UmAmAmAmAmCmUmAmAmCmCmUmGmUmCm
Tmt25S-3143-chimera	GmCmAmUmGmCmGmUmCmGmCm(TTCT)AmGmCmCmCmGmGmAmUm
Tmt18S_F1	(TACCTGGTTGATCCTGCCAG)
Tmt18S_F445	(AATCCTGACACGGGGAGGTA)
Tmt18S_F1196	(CGGGGAAACTTACCAGGTCC)
Tmt18S_R544	(CACCAGACTTGCCCTCCAAT)
Tmt18S_R1170	(GTCAAATTAAGCCGCAGGCT)
Tmt18S_R1488	(GTCGGCCAAGGCTATAAGCT)
Tmt18S_R1764	(ACCTACGGAAACCTTGTTAC)
Tmt25S_F1	(GCGACCCCAGGTCAGGC)
Tmt25S_F261	(TACGAGTCGGGTTGTTTGGG)
Tmt25S_F780	(GAAGGGTTCGAGTGTGAGCA)
Tmt25S_F1109	(ATCAAGAGCTCCAAGTGGGC)
Tmt25S_F1478	(ACTTTGAAGGCCGAAGAGGG)
Tmt25S_F1847	(AACCGCATCAGGTCTCCAAG)
Tmt25S_F2194	(TCTGCCCAGTGCTCTGAATG)
Tmt25S_F2938	(GGGAACGTGAGCTGGGTTTA)
Tmt25S_R780	(TGCTCACACTCGAACCCTTC)
Tmt25S_R1189	(GATCTAGGTTAGCGCGCAGT)
Tmt25S_R2956	(ACCTGTCTCACGACGGTCTA)
Tmt25S_R3367	(ACGAATCGGAGCGTCAAAGG)

a. Nm refers to 2'-O-methyl ribonucleotide. Deoxyribonucleotides are indicated in parentheses.

Oligonucleotide	DNA/RNA	Note
Tmt18S-270-chimera	DNA/RNA	Used for RNase H digestion to produce Fragment H1 or H2.
c18S-385-chimera	DNA/RNA	Used for RNase H digestion to produce Fragment H2 or H3.
c18S-580-chimera	DNA/RNA	Used for RNase H digestion to produce Fragment H3 or H4.
Tmt18S-831-chimera	DNA/RNA	Used for RNase H digestion to produce Fragment H4 or H5.
h18S-1210-chimera	DNA/RNA	Used for RNase H digestion to produce Fragment H5 or H6.
Tmt18S-1332-chimera	DNA/RNA	Used for RNase H digestion to produce Fragment H7 or H8.
Tmt18S-1597-chimera	DNA/RNA	Used for RNase H digestion to produce Fragment H8 or H9.
Tmt18S-1679-chimera	DNA/RNA	Used for RNase H digestion to produce Fragment H9 or H10.
Tmt25S-418-chimera	DNA/RNA	Used for RNase H digestion to produce Fragment H10 or H11.
Tmt25S-643-chimera	DNA/RNA	Used for RNase H digestion to produce Fragment H11, H12 or H14.
Tmt25S-850-chimera	DNA/RNA	Used for RNase H digestion to digest the RNase T1 fragment 28S_1764-1791 into two fragments.
h28S-1866-chimera	DNA/RNA	Used for RNase H digestion to produce Fragment H12 or H13.
Tmt25S-1521-chimera	DNA/RNA	Used for RNase H digestion to produce Fragment H13, H14 or H15.
Tmt25S-1859-chimera	DNA/RNA	Used for RNase H digestion to produce Fragment H15 or H16.
Tmt25S-2119-chimera	DNA/RNA	Used for RNase H digestion to produce Fragment H16 or H17.
Tmt25S-2232-chimera	DNA/RNA	Used for RNase H digestion to produce Fragment H18.
h28S-3892-chimera	DNA/RNA	Used for RNase H digestion to produce Fragment H17.
Tmt25S-2611-chimera	DNA/RNA	Used for RNase H digestion to produce Fragment H18 or H19.
Tmt25S-2703-chimera	DNA/RNA	Used for RNase H digestion to produce Fragment H19 or H20.
Tmt25S-2755-chimera	DNA/RNA	Used for RNase H digestion to produce Fragment H20, H21, H22 or H23.
h28S-4443-chimera	DNA/RNA	Used for RNase H digestion to produce Fragment H23 or H24.
h28S-4516-chimera	DNA/RNA	Used for RNase H digestion to produce Fragment H22, H24, H25 or H29.
p25S-3044-chimera	DNA/RNA	Used for RNase H digestion to produce Fragment H25 or H26.
h28S-4563-chimera	DNA/RNA	Used for RNase H digestion to produce Fragment H26 or H27.
Tmt25S-3143-chimera	DNA/RNA	Used for RNase H digestion to produce Fragment H27 or H28.
Tmt18S_F1	DNA	Used for PCR and sequencing primer.
Tmt18S_F445	DNA	Used for sequencing primer.
Tmt18S_F1196	DNA	Used for sequencing primer.
Tmt18S_R544	DNA	Used for sequencing primer.
Tmt18S_R1170	DNA	Used for sequencing primer.
Tmt18S_R1488	DNA	Used for sequencing primer.
Tmt18S_R1764	DNA	Used for reverse transcription, PCR and sequencing primer.
Tmt25S_F1	DNA	Used for PCR and sequencing primer.
Tmt25S_F261	DNA	Used for sequencing primer.
Tmt25S_F780	DNA	Used for sequencing primer.
Tmt25S_F1109	DNA	Used for sequencing primer.
Tmt25S_F1478	DNA	Used for sequencing primer.
Tmt25S_F1847	DNA	Used for sequencing primer.
Tmt25S_F2194	DNA	Used for sequencing primer.
Tmt25S_F2938	DNA	Used for sequencing primer.
Tmt25S_R780	DNA	Used for sequencing primer.
Tmt25S_R1189	DNA	Used for sequencing primer.
Tmt25S_R2956	DNA	Used for sequencing primer.
Tmt25S_R3367	DNA	Used for reverse transcription, PCR and sequencing primer.
